# Integration of single‐cell and bulk RNA‐sequencing to analyze the heterogeneity of hepatocellular carcinoma and establish a prognostic model

**DOI:** 10.1002/cnr2.1935

**Published:** 2023-11-22

**Authors:** Yaping Mu, Ding Zheng, Qinghua Peng, Xiaodong Wang, Yurong Zhang, Yue Yin, Encheng Wang, Fei Ye, Jing Wang

**Affiliations:** ^1^ The School of Integrated Traditional Chinese and Western Medicine Southwest Medical University Luzhou Sichuan China; ^2^ Department of Hepatobiliary The Affiliated Traditional Chinese Medicine Hospital of Southwest Medical University Luzhou Sichuan China; ^3^ School of Traditional Chinese Medicine Beijing University of Traditional Chinese Medicine Beijing China

**Keywords:** bulk RNA‐sequencing, hepatocellular carcinoma, heterogeneity, prognostic model, single‐cell RNA‐sequencing

## Abstract

**Background:**

The highly heterogeneous nature of hepatocellular carcinoma (HCC) results in different responses and prognoses to the same treatment in patients with similar clinical stages.

**Aims:**

Thus, it is imperative to investigate the association between HCC tumor heterogeneity and treatment response and prognosis.

**Methods and Results:**

At first, we downloaded scRNA‐seq, bulk RNA‐seq, and clinical data from TCGA and GEO databases. We conducted quality control, normalization using SCTransform, dimensionality reduction using PCA, batch effect removal using Harmony, dimensionality reduction using UMAP, and cell annotation‐based marker genes on the scRNA‐seq data. We recognized tumor cells, identified tumor‐related genes (TRGs), and performed cell communication analysis. Next, we developed a prognostic model using univariable Cox, LASSO, and multivariate Cox analyses. The signature was evaluated using survival analysis, ROC curves, C‐index, and nomogram. Last, we studied the predictability of the signature in terms of prognosis and immunotherapeutic response for HCC, assessed a variety of drugs for clinical treatment, and used the qRT‐PCR analysis to validate the mRNA expression levels of prognostic TRGs.

**Conclusion:**

To conclude, this study expounded upon the influence of tumor cell heterogeneity on the prediction of treatment outcomes and prognosis in HCC. This, in turn, enhances the predictive ability of the TNM staging system and furnishes novel perspectives on the prognostic assessment and therapy of HCC.

## 1 INTRODUCTION

Primary liver cancer is a prevalent malignancy worldwide, with hepatocellular carcinoma (HCC) being the most frequently occurring pathological subtype.^1^ HCC etiology is predominantly attributed to exposure to various risk factors, including hepatitis virus infection, aflatoxin B1 contamination, and alcohol misuse. Several treatment modalities, including but not limited to surgery, radiofrequency or microwave ablation, embolization, and sorafenib, have been developed to manage HCC.^2^ Despite significant advancements in treatments, the overall prognosis of HCC patients remains bleak.^3^ Notably, the survival outcome can significantly vary for patients with similar clinical stages, highlighting the heterogeneity of the tumor. Therefore, it is imperative to establish biomarkers capable of predicting HCC patient prognosis and facilitating the customization of the most appropriate treatment tactics.^4^ To this end, additional research is indispensable to augment our understanding of the molecular underpinnings of HCC, identify clinically significant biomarkers for the disease, and engineer innovative targeted therapies. Additionally, an improved prediction system utilizing not only clinical information but also molecular and genetic data could facilitate personalized treatment and ultimately lead to better outcomes for HCC patients.

Single‐cell RNA sequencing (scRNA‐seq) has become a robust tool to analyze gene expression in individual cells, providing an unprecedented level of resolution in the assessment of cellular heterogeneity within a tissue or tumor.^5^ With its ability to isolate individual cells and capture the entire transcriptome, scRNA‐seq has become a valuable tool for identifying novel cell subpopulations, characterizing cell states, and elucidating molecular mechanisms underlying complex biological processes. Furthermore, the combination of scRNA‐seq and bulk RNA sequencing has been proposed as an effective approach to overcome the limitations of each method and provide a more comprehensive analysis of tissue or tumor heterogeneity.^6^ Researchers can extrapolate information from rare cell subpopulations identified in scRNA‐seq data to the overall cell populations represented in bulk RNA‐seq.^7^, ^8^, ^9^, ^10^ Thus, this method has the potential to improve the accuracy of prognostic models and better guide individualized treatment decisions. A key challenge in implementing this approach is the robust construction and validation of relevant models to predict patient prognosis and treatment response. The lack of rigorous validation and experimental verification has limited the full potential of this approach to improve clinical outcomes in cancer patients. Further efforts are needed to establish robust models and validate their efficacy for predicting clinical outcomes in large cohorts of patients, which will require close collaboration between bioinformaticians, oncologists, and other experts in this field.

The use of scRNA‐seq data to systematically identify tumor‐related genes (TRGs) has become an attractive method for improving the accuracy of predicting prognosis and developing personalized treatment regimens for HCC patients. These TRGs can then be utilized to construct prognostic risk models that quantify patient survival based on gene expression patterns. The authors utilized scRNA‐seq data to identify TRGs associated with HCC progression, which were subsequently employed to develop a prognostic risk model. The model was constructed using data from TCGA and validated with data from the GEO. To enhance the accuracy of the model, the authors validated the expression of the TRGs in the model using qPCR. The findings from the study potentially offer valuable insights into the development of prognostic risk models that can accurately predict the survival of HCC patients, leading to the customization of optimal treatment regimens for different subgroups of HCC patients.

## 2 MATERIALS AND METHODS

### 2.1 Preparation of data

In the area of cancer genomics, scRNA‐seq is a powerful and increasingly popular approach for analyzing transcriptomic heterogeneity at the single‐cell level. The scRNA‐seq data were acquired from the GSE149614 dataset in the publicly available GEO database. The dataset contains a large number of cells from multiple HCC samples, providing a comprehensive view of the cellular heterogeneity within this disease.^11^ This set consisted of 8 nontumorous liver tissues and 10 tumor liver tissues. Furthermore, bulk RNA‐seq and relevant clinical data were extracted from two different sources, namely the GEO‐GSE76427 and TCGA‐HCC datasets.^12^ The first dataset, GSE76427, consisted of 52 adjacent non‐tumor and 115 tumor liver tissues, whereas the second dataset, TCGA‐HCC, consisted of 50 nontumor and 374 tumor liver tissues. To minimize any potential biases that may arise from missing or incomplete data, we eliminated data with missing survival information. By doing so, we were able to ensure the reliability and accuracy of our study results, as the exclusion of these observations can help to reduce any potential confounding effects or skewness within the dataset. Overall, the rigorous and systematic approach adopted in this study highlights the importance of carefully controlling for potential biases in order to obtain valid and meaningful conclusions.

### 2.2 Processing of scRNA‐seq data

The R package “Seurat” (v4.2.0) was utilized for data processing and visualization.^13^ Cells with gene numbers <60 or >6000, UMI > 60 000, hemoglobin percentage >1%, and mitochondrial gene percentage >10% were excluded, and 56 025 cells were finally obtained. We then performed normalization on this object using SCTransform, dimensionality reduction (1:30) using principal component analysis (PCA), batch effect removal using Harmony, dimensionality reduction using UMAP (resolution = 0.5), and cell annotation‐based marker genes.^14^, ^15^

The CopyKAT is able to infer the chromosomal ploidy of cells and thus whether they are normal (diploid) or tumor cells (aneuploid). The “Copykat” R package (v1.1.0) was used for the identification of tumor cells and genes that are highly expressed in tumor cells were identified using the function FindMarkers between normal and tumor cells (logFC >0.25 and adjusted *p*‐value < .05).^16^ These TRGs that are highly expressed in the tumor will be used for subsequent bulk RNA‐seq analysis. Monocle could be applied to restore single‐cell gene expression kinetics in a wide range of cellular processes, such as oncogenic transformation.^17^ Monocle 2 (v2.24.1) was employed to build normal cell‐tumor cell trajectories for both normal hepatocytes and cancer cells. We examined the expression changes of genes in the model during cell differentiation trajectories.^18^ Communication between different cell types is important for them to decide their own direction; however, this communication is very complex. To explore cell–cell communications in HCC, the “CellChat” (v1.5.0) was implemented, and we only retained the results that contained the following genes: FGFR4, EGFR, VEGFR, and MET.^19^

### 2.3 Construction and validation of risk signature

The study was to identify prognostic TRGs and develop a risk model for forecasting the survival outcomes of HCC. To achieve this objective, we performed statistical analyses and validation methods. First, we performed univariate Cox analysis to identify prognostic TRGs with statistical significance (*p* < .05). Next, we utilized LASSO analysis to identify candidate TRGs and establish a risk model by performing multivariate Cox analysis. To test and validate the effectiveness of the developed model, we calculated the risk score for each HCC patient using a specially designed formula: $\sum_{i=1^{\text{βiSi}}}^{k}$. Kaplan–Meier analysis was employed to compare the survival outcomes between these different risk groups. An external validation dataset (GEO‐GSE76427) was used to validate the prognostic model we constructed.

Furthermore, we evaluated the predicted reliability of survival by utilizing ROC curves and AUC. We assessed the survival disparities between different risk score groups within each subgroup to evaluate the prognostic model's applicability to patients with distinct clinical characteristics. In addition, we employed univariate and multivariate Cox analyses to validate the model as an independent predictor of prognosis and calculated the C‐index to evaluate the efficacy of the signature compared to clinical features. We developed a nomogram that integrates the signature and clinical characteristics to predict the survival rates of HCC patients.

### 2.4 GO and KEGG analyses

To obtain insights into the molecular mechanisms, we utilized differential gene expression analysis to identify significant DEGs (|logFC >1| and FDR < 0.05) between these groups. We performed GO and KEGG analyses on the identified DEGs to uncover the underlying biological processes and pathways that characterize each group (*p* < .05).^20^ The integration of these methods allowed for a comprehensive understanding of the functional implications of DEGs between different groups.

### 2.5 Evaluation of the immune landscape

We performed mutation analysis to identify the gene mutations between different risk groups. We also calculated TIDE and TMB scores to predict the response to immunotherapy in different groups.^21^, ^22^ Furthermore, we investigated the survival differences between different TMB groups and between different risk groups. To explore the differences in immune cell subpopulations between different risk groups, we leveraged various algorithms.^23^, ^24^, ^25^, ^26^, ^27^, ^28^, ^29^ We then used a ssGSEA to evaluate the differences in immune function between different risk groups. Additionally, we analyzed the expression levels of various immune checkpoint genes (ICGs) to investigate the potential of immunotherapy for different risk groups. These results provide valuable clinical insight for developing novel therapeutic strategies to combat HCC progression.

### 2.6 Identification of antitumor drugs

We aimed to evaluate the efficacy of commonly used anti‐tumor drugs for the clinical treatment of HCC. To this end, we employed the “pRRophetic” (v:0.5) to calculate the IC50 of drugs commonly used for HCC treatment.^30^ We then compared the IC50 values between different groups. This analysis helped determine which drugs may be most effective for different subgroups of HCC patients. The IC50 values of the drugs could also provide information on the appropriate dosages of the drugs for successful treatment.

### 2.7 Correlation between TRG signature and malignant features

We integrated feature gene expressions and model pathway activity using the *z*‐score technique.^31^, ^32^ We analyzed gene sets related to angiogenesis, cell cycle, EMT, and prognostic TRGs using the GSVA method.^33^, ^34^ To compute the GSVA scores, we first identified gene sets that were related to the above pathways. These gene sets were then used to compute pathway activity scores for each sample. Specifically, the gene expression data of each sample was converted to a z‐score, which was used as the input for GSVA analysis. The resulting scores were expressed as *z*‐scores for each pathway, which allowed for comparison across different groups and the identification of pathways that were activated or inhibited in different subgroups of HCC patients. Using this approach, we were able to model pathway activity in key processes such as angiogenesis, EMT, and cell cycle, which are considered to be dysregulated in HCC. We also incorporated prognostic TRGs into this analysis to evaluate their functional significance and impact on pathway activity.

### 2.8 Validation by Human Protein Atlas

Specifically, we investigated the protein levels of the identified prognostic TRGs in both normal and tumor tissues. This approach allowed us to confirm the expression patterns of these TRGs at the protein level and to understand their potential biological functions in HCC tumorigenesis. The HPA database provides a comprehensive resource containing immunohistochemical images and protein expression data for multiple tissue types and cancer subtypes. By analyzing the protein expression profiles of TRGs in normal tissues and HCC tumor tissues, we were able to evaluate the association of these genes with HCC pathogenesis.

### 2.9 Experimental material preparation

#### 2.9.1 Experimental cells

LO2, Hepg2, Huh7, and SMMC‐7721 cells were purchased from Procell Life Science & Technology Co.

#### 2.9.2 Reagent

Chloroform‐free RNA extraction kit (centrifuge column type) was from Bioteke (RP55011). DMEM (high sugar) was purchased from Thermo Fisher Biochemicals (Beijing) Co. RPMI 1640 Medium was purchased from Cytiva. FBS Premium was purchased from PAN. The primers were designed and synthesized by Shanghai Bioengineering Co. (Table 1).

**TABLE 1:** Primer sequences of the genes.

Gene	–	Sequences
S100A9	Forward	GCACCCAGACACCCTGAACCA
	Reverse	TGTGTCCAGGTCCTCCATGATG
PPP1R16A	Forward	CAATGCCTGTGACAGTGAGTGC
	Reverse	GGTGTTGACCGCCAGGAGATTG
PRDX6	Forward	CAGCTACCACTGGCAGGAACTT
	Reverse	GGAAGGACCATCACACTATCCC
GAGE2A	Forward	GGAACCAGCAACTCAACGTCAG
	Reverse	GGACCATCTTCACACTCACACC
LDHA	Forward	GGATCTCCAACATGGCAGCCTT
	Reverse	AGACGGCTTTCTCCCTCTTGCT
TMEM106C	Forward	CCGCATTCAGTCCTTGTGGATG
	Reverse	CACCGTGTAGAAGTTGGAGTTCC
RTN3	Forward	CTTACCTCATCCTGGCTCTTCTC
	Reverse	GACAGAGTAATGTCTACGTCCAG
DNAJB4	Forward	TTAAAGAGGTCGCAGAAGCTTATG
	Reverse	GATCGCCATGAAAGGTGTACCG
β‐Actin	Forward	CCTGGCACCCAGCACAAT
	Reverse	GCCGATCCACACGGAGTA

#### 2.9.3 Main instruments and equipment

Real‐time PCR was from Roche Diagnostics GmbH (LightCycler 480 II). The enzyme marker was from Burton, USA (Synergy 2). The Veriti gradient PCR instrument was from ABI, USA. Clean bench was from Suzhou Antai Airtech (SJ‐2J‐2F). The electrothermal constant temperature incubator was from Jiaxing Zhongxin Medical Instrument (DNP‐9052).

### 2.10 Cell culture, RNA extraction, and qRT‐PCR

Hepatocyte LO2 and HCC cell lines (HepG2, Huh7, and SMMC‐7721) were cultured in a medium containing fetal bovine serum and incubated at 37°C and 5% CO_2_. Total RNA was extracted from each group of cells separately using a chloroform‐free RNA kit. The RNA purity was assessed by an enzyme marker. Reverse transcription was performed by Veriti gradient PCR instrument, and finally, the relative expression of each group of genes was measured by PCR instrument. The signals were normalized to β‐actin and the relative expression of mRNA in each sample was calculated by the 2 − ΔΔ*Ct* method. All PCR assays were performed in three replicates. Statistical analysis was performed in SPSS 17.0 using Student's *t*‐test.

### 2.11 Statistical analyses

The statistical analyses for the research were conducted using R software version 4.2.0, which is a commonly used open‐source statistical analysis tool. The Wilcoxon test was utilized to compare and evaluate the expressions across the different groups. The significance level for determining statistical significance was set at *p* < .05.

## 3 RESULTS

### 3.1 Quality control and dimensionality reduction

**FIGURE 1:**
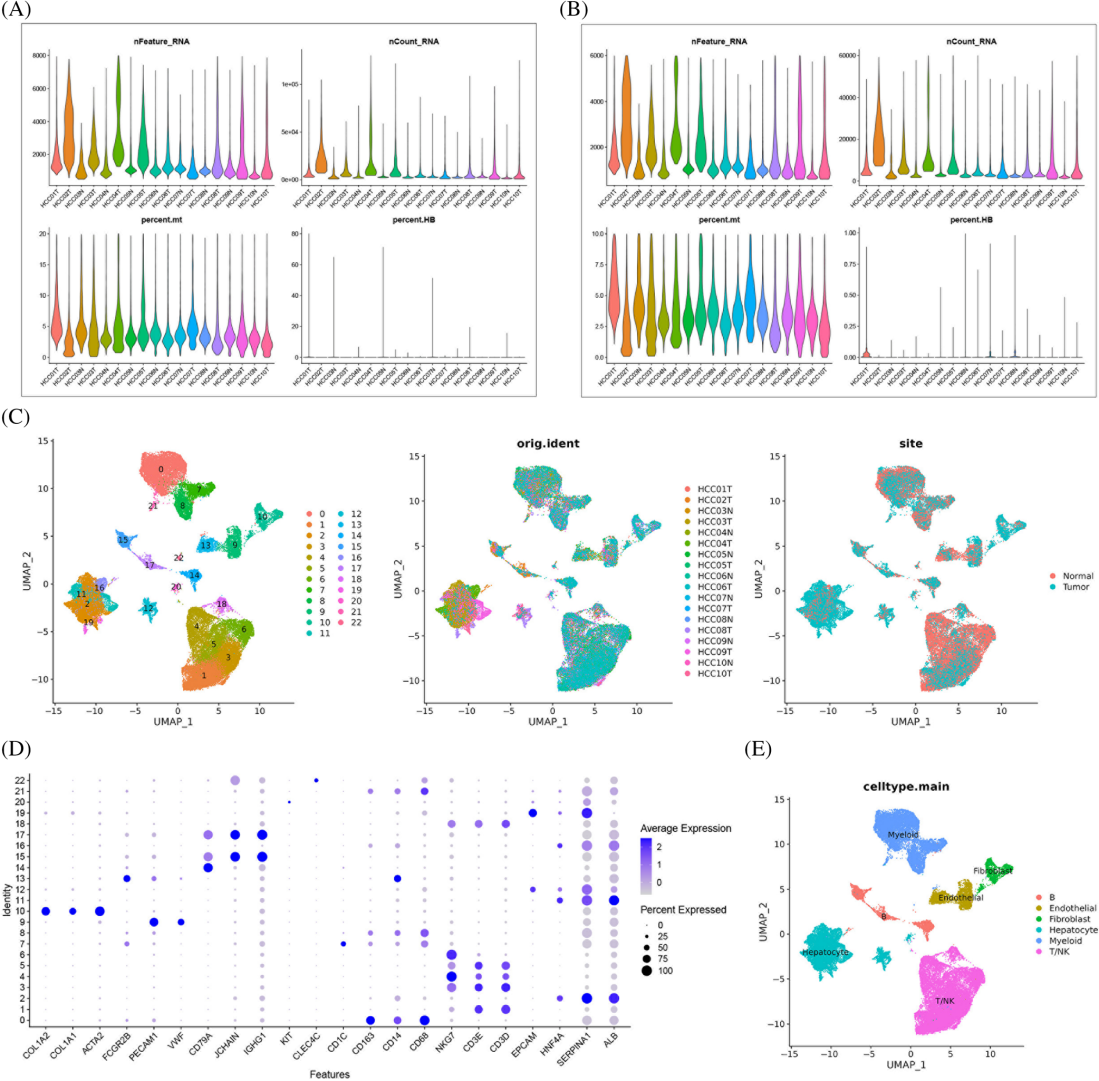
(A, B) Eight nontumor liver tissues, 10 tumor liver tissues, and 56 025 cells from the GSE149614 dataset after quality control. (C) Normalization using SCTransform, dimensionality reduction (1:30) using PCA, batch effect removal using Harmony, and dimensionality reduction using UMAP (resolution = 0.5). (D) The expression of marker genes in each cluster. (E) Six major cell types in HCC.

**FIGURE 2:**
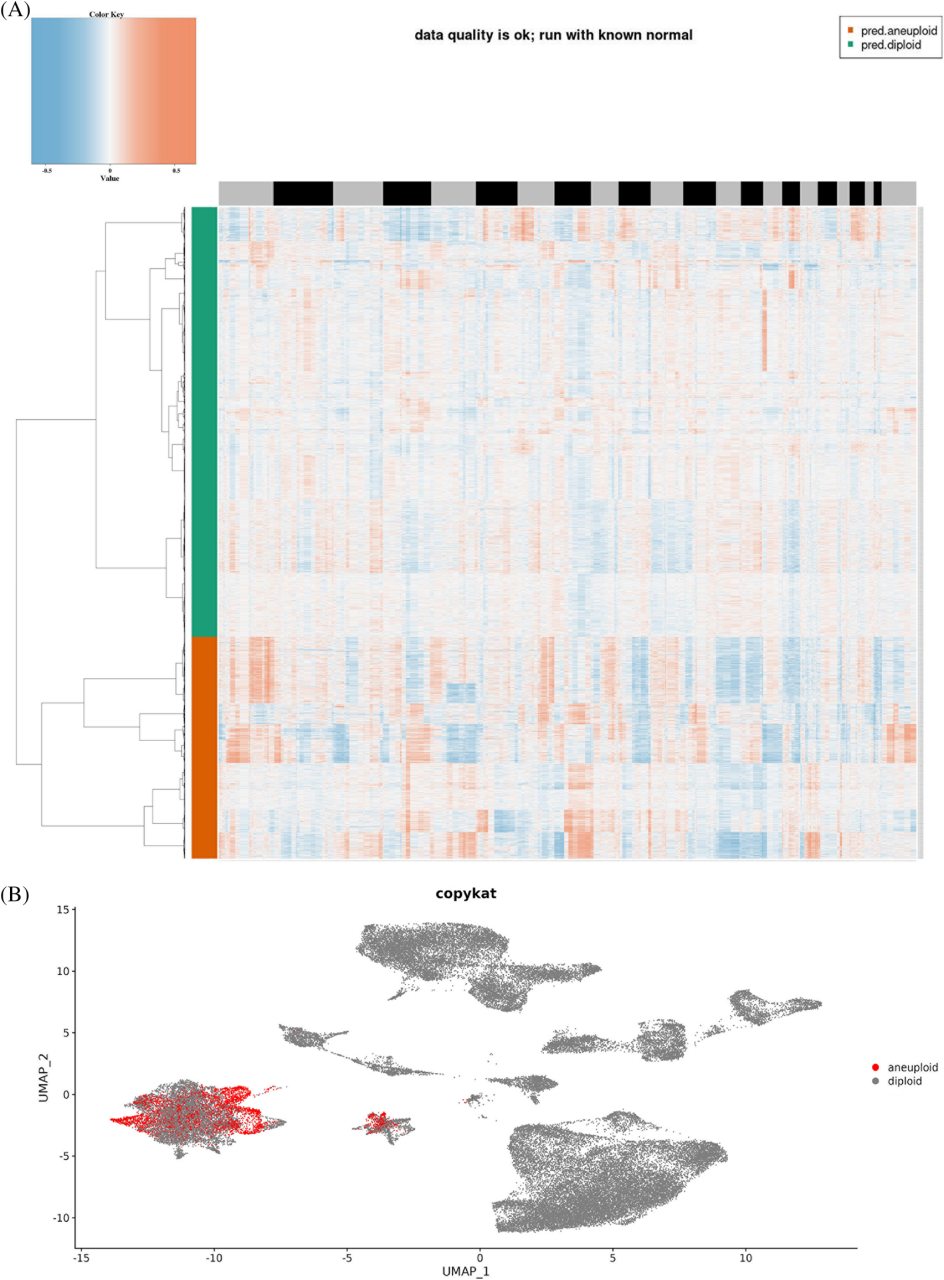
(A) Clustering heat map of single cell copy number profiles evaluated by R package “Copykat.” Orange represents aneuploid, and green represents diploid. (B) Distribution of aneuploids and diploids in UMAP.

In the research, we obtained 8 non‐tumor liver tissues, 10 tumor liver tissues, and 56 025 cells from the GSE149614 dataset after quality control (Figure 1A,B). We then performed normalization on this object using SCTransform, dimensionality reduction (1:30) using PCA, batch effect removal using Harmony, and dimensionality reduction using UMAP (resolution = 0.5) (Figure 1C). Next, we annotated the clustered cell clusters, and the marker genes used can be found in (Figure 1D,E and Table S1). As there was no apparently highly expressed marker gene in cluster 22, we removed it. We used CopyKAT to identify aneuploids as tumor cells and diploids as normal cells and displayed them in UMAP (Figure 2A,B). TRGs were then identified by the function FindMarkers (only genes highly expressed in tumor cells were retained) (Table S2). Overall, our study provides a comprehensive analysis of liver tissue and cell types, and the identification of TRGs may have significant implications for understanding and potentially treating liver cancer.

### 3.2 Pseudotime analysis and cell–cell communication analysis

**FIGURE 3:**
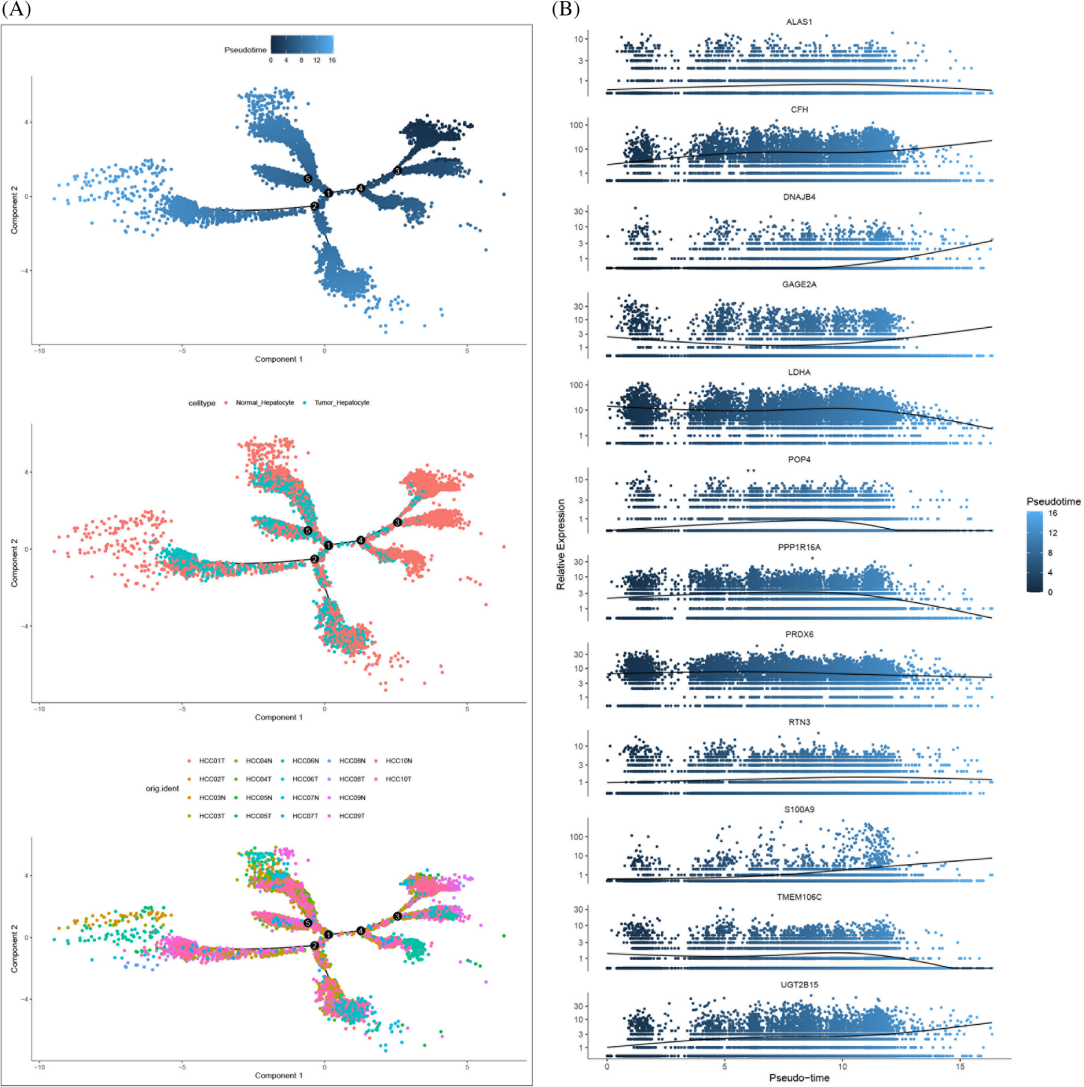
(A) Pseudotime and trajectory analysis on different classifications such as pseudotime, cell type, and different samples. (B) The expression of 12 prognostic TRGs in the pseudotime and trajectory analysis.

**FIGURE 4:**
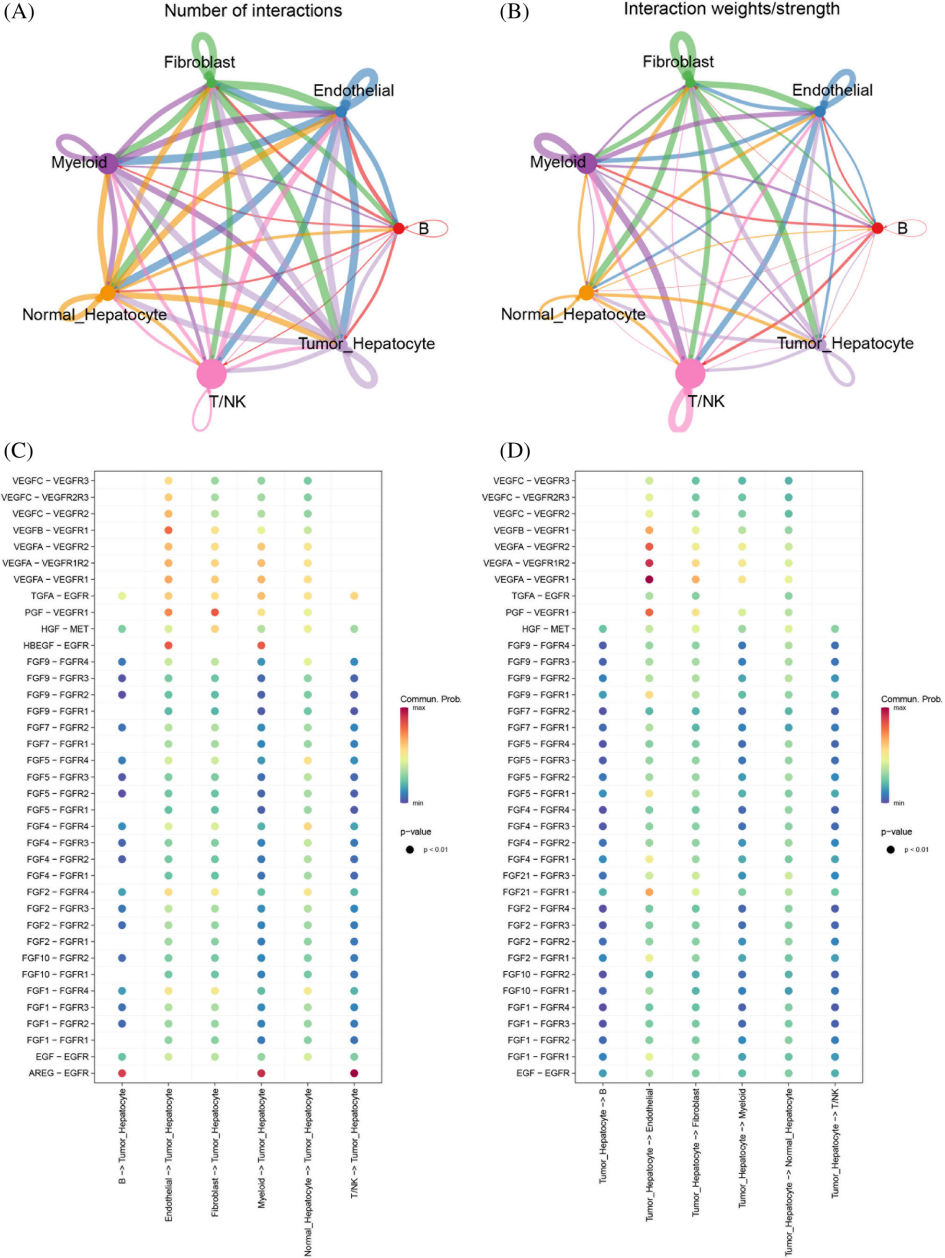
(A, B) The circle diagram shows the number and intensity of interactions between the major cell types in HCC. (C) The specific intercommunication networks between tumor cells and other cell types.

We constructed cell differentiation trajectories of normal hepatocytes and cancer cells using Monocle 2, which were shown according to different pseudotimes, cell types, and different samples (Figure 3A). Subsequently, we also measured the variations in the expression of genes in the models constructed by the following analysis and found that most of the genes increased with the increase of the pseudotime (Figure 3B). These findings suggest a dynamic regulatory process associated with the progression of cell differentiation. To investigate the interactions between tumor cells and other cell types in HCC, we constructed a cell‐to‐cell communication network using “CellChat.” Figure 4A,B shows the quantity and quality of communication between different cells, respectively. Figure 4C,D shows the specific intercommunication networks between HCC cells and other cell types, and the results indicated that communication between HCC and other cell types was abundant. Remarkably, our results revealed a substantial abundance of communication between HCC and various non‐tumor cell types.

### 3.3 Construction and validation of risk assessment signature

**FIGURE 5:**
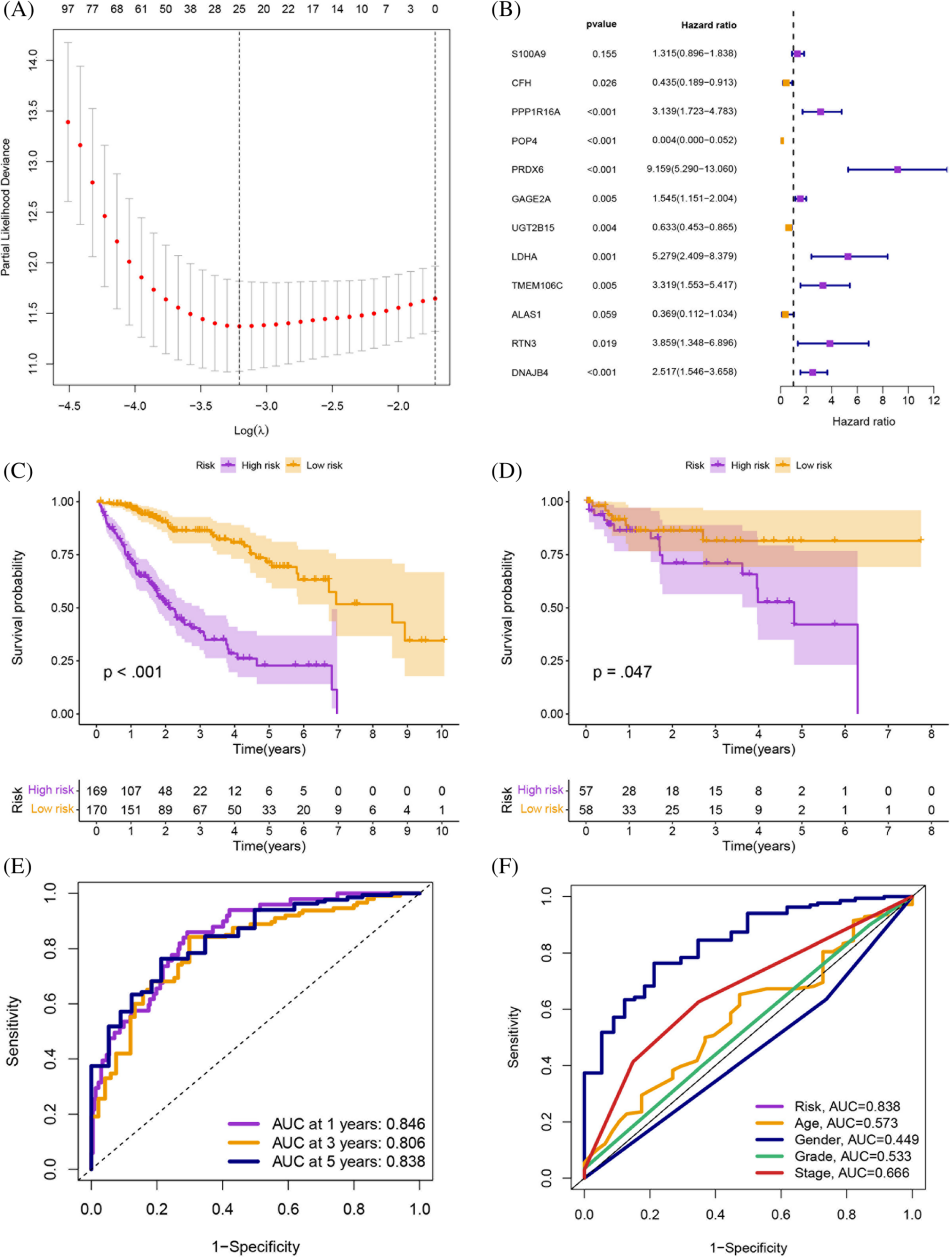
(A) 25 prognostic TRGs by LASSO analysis. (B) 13 prognostic TRGs by multivariate Cox analysis. (C, D) The survival analysis from TCGA‐HCC and GSE76427. (E) The AUC values were all greater than 0.8. (F) The AUC of the model was also higher than other clinical features.

**FIGURE 6:**
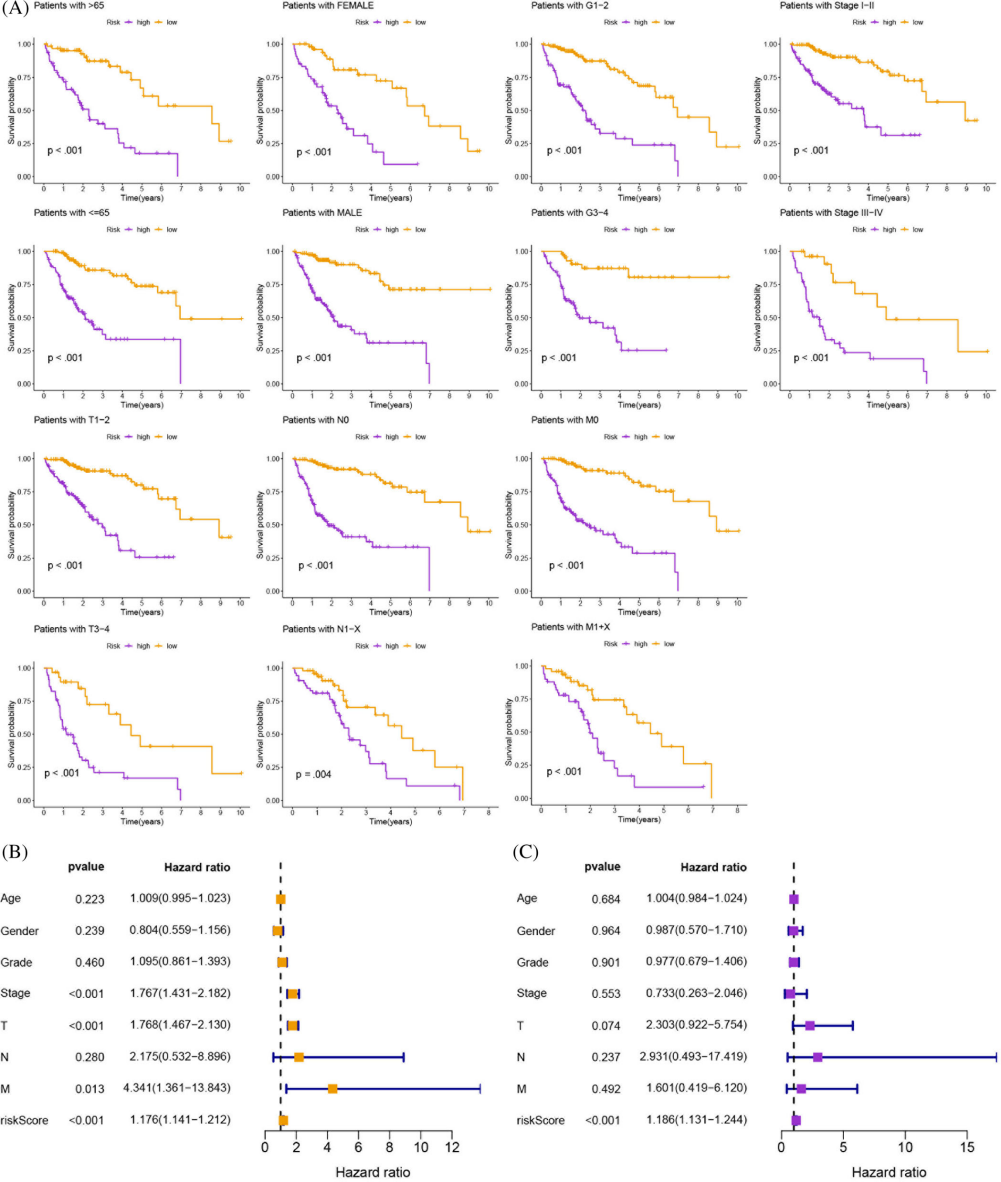
(A) Based on the different clinical subgroups, patients in the low‐risk group still had longer survival times, indicating that the model is applicable to patients with various clinical features. (B, C) The risk score was an independent prognostic factor.

**FIGURE 7:**
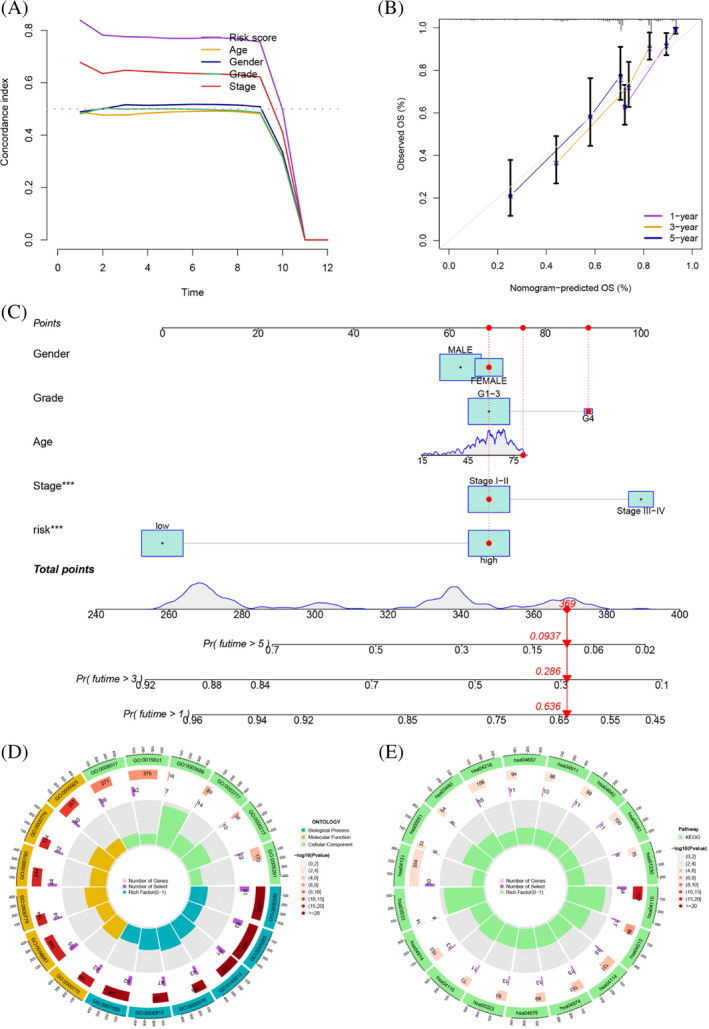
(A) The *C*‐index suggested that the model was a better predictor of HCC prognosis than conventional clinical features. (B) The observed 1‐, 3‐, and 5‐year survival rates showed a strong agreement with the expected rates in the correlation plot. (C) A nomogram containing the signature and clinical features. (D) The BP terms of these DEGs were related to nuclear division, organelle fission, and mitotic nuclear division. The CC terms were related to chromosome, centromeric region, chromosomal region, and condensed chromosome, centromeric region. On the other hand, the MF terms were predominantly related to microtubule binding, tubulin binding, and DNA replication origin binding. (E) KEGG pathway analysis presented various pathways that were enriched in DEGs, including cell cycle, ECM‐receptor interaction, oocyte meiosis, protein digestion and absorption, bile secretion, and rheumatoid arthritis.

Univariate Cox analysis was conducted to identify prognostic TRGs from the TCGA‐HCC (*p* < .05; Table S3), LASSO analysis was performed to identify these prognostic TRGs (Figure 5A), and multivariate Cox analysis was applied to identify these remaining prognostic TRGs to establish a prognostic signature (Figure 5B). The survival analysis showed a higher survival time in the low‐risk group (*p* < .001; Figure 5C), and the results were the same in the validation set from GSE76427 (*p* = .047; Figure 5D). This prognostic risk assessment signature was adopted to predict the 1‐, 3‐, and 5‐year survival rates of HCC patients, and the AUC values were all greater than 0.8 (Figure 5E). The AUC of the model was higher than other clinical features, including age, gender, grade, and stage, suggesting that it is more reliable (Figure 5F). Based on the different clinical subgroups, patients in the low‐risk group still had longer survival times, indicating that the model is applicable to patients with various clinical features (Figure 6A). Univariate and multivariate Cox analyses indicated that the risk score was an independent prognostic factor for HCC patients (*p* < .001; Figure 6B,C). The *C*‐index suggested that the model was a better predictor of HCC prognosis than conventional clinical features (Figure 7A). The observed 1‐, 3‐, and 5‐year survival rates showed strong agreement with the expected rates in the correlation plot (Figure 7B). We constructed a nomogram containing the signature and clinical features that could be used to accurately predict the survival of HCC patients (Figure 7C). Collectively, the above findings demonstrate that the prognostic model we have developed exhibits a high degree of accuracy and stability.

### 3.4 Assessment of immunological landscape

**FIGURE 8:**
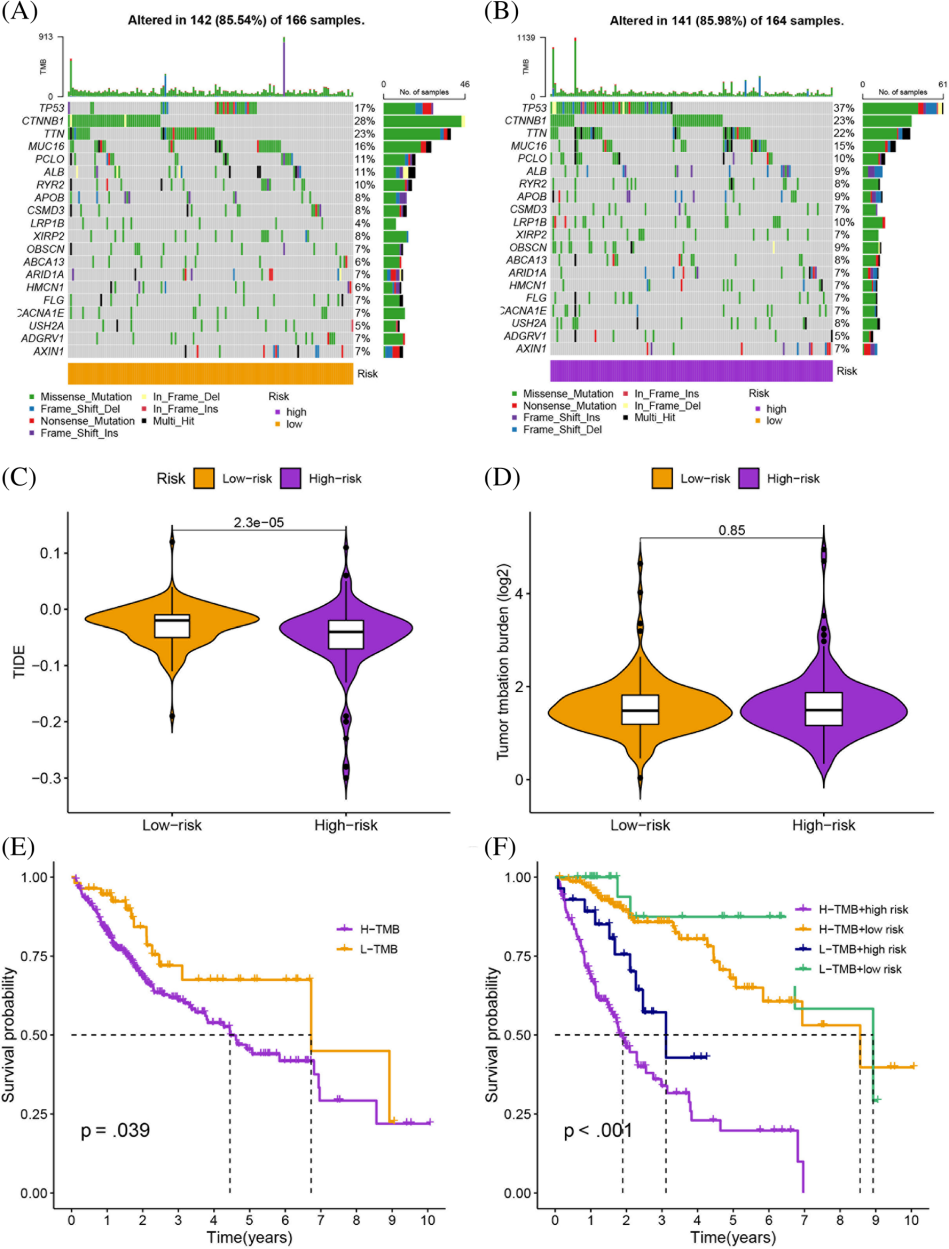
(A, B) The specific mutated genes varied greatly in different groups. (C) The high‐risk group had lower TIDE scores. (D) There are no differences in TMB scores between different risk groups. (E, F) Combined TMB and risk scores were significantly different for combined survival.

**FIGURE 9:**
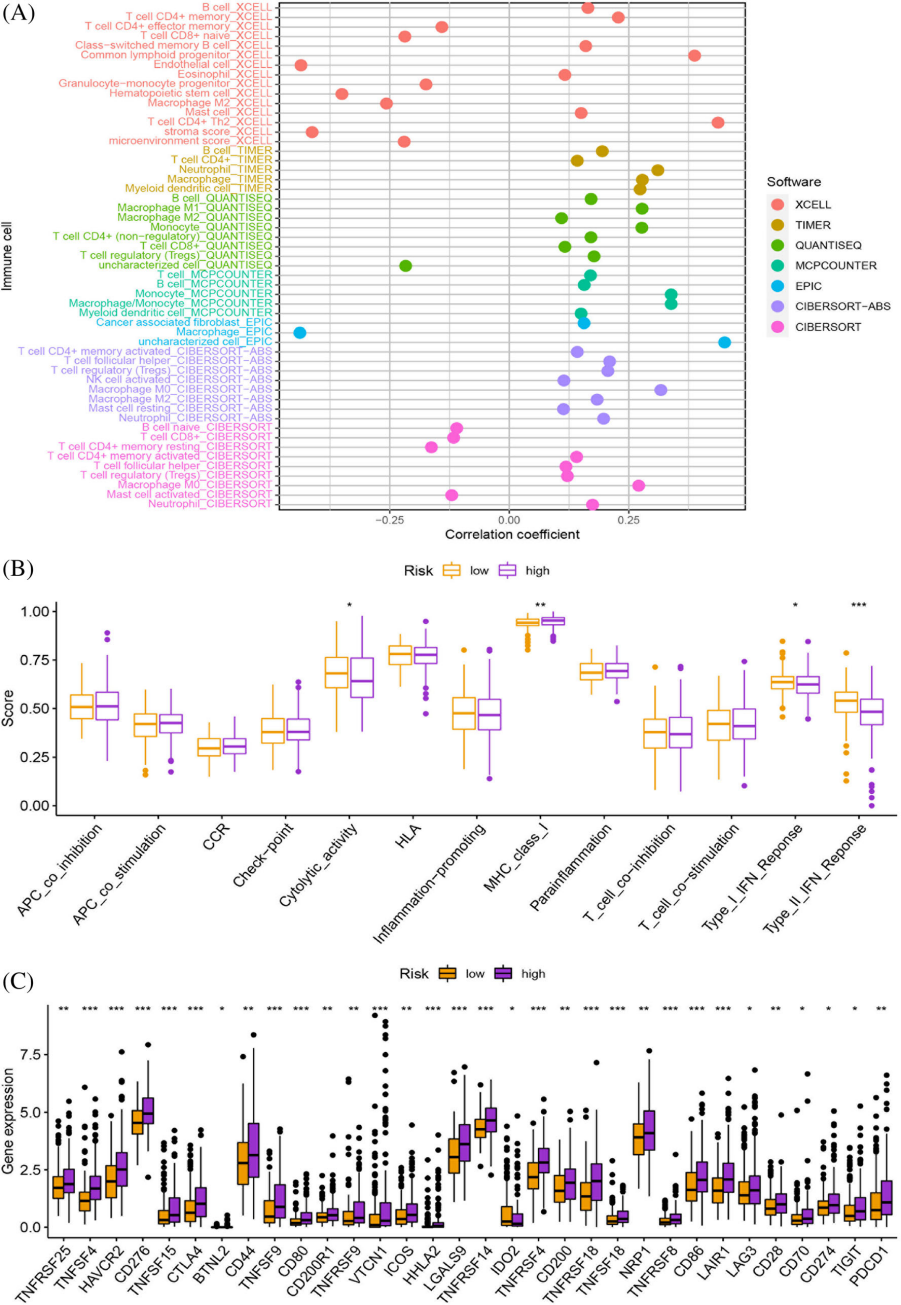
(A) The enrichment of B cells, CD4+ T cells, regulatory T cells, NK cells, neutrophils, myeloid dendritic cells, monocytes, and M1 macrophages was positively correlated with the risk score. (B) Some immune functions, including cytolytic activity, MHC class I, type I IFN response, and type II IFN response, were statistically different between the different risk groups. (C) The expression of ICGs was also statistically different between different risk groups.

To assess the different molecular mechanisms between different groups, we identified 986 DEGs between different groups. The GO analysis revealed that the BP terms of these DEGs were related to nuclear division, organelle fission, and mitotic nuclear division. The CC terms were related to chromosome, centromeric region, chromosomal region, and condensed chromosome, centromeric region. On the other hand, the MF terms were predominantly related to microtubule binding, tubulin binding, and DNA replication origin binding (Figure 7D and Table S4). KEGG pathway analysis presented various pathways that were enriched in DEGs, including cell cycle, ECM‐receptor interaction, oocyte meiosis, protein digestion and absorption, bile secretion, and rheumatoid arthritis (Figure 7E and Table S5). The gene mutation analysis showed that although the frequency of gene mutations was similar in the high‐ and low‐risk groups, the specific mutated genes varied greatly (Figure 8A,B). The high‐risk group had lower TIDE scores, suggesting that the high‐risk group was likely to be more responsive to immunotherapy (*p* < 0.001; Figure 8C). Although there were no differences in TMB scores between different risk groups, survival analysis showed that different TMB and risk groups had statistically different survival rates, which suggested that the combination of TMB scores could better predict the prognosis of HCC patients (Figure 8D–F). The enrichment of B cells, CD4+ T cells, regulatory T cells, NK cells, neutrophils, myeloid dendritic cells, monocytes, and M1 macrophages was positively correlated with the risk score (Figure 9A; Tabls S6). Some immune functions, including cytolytic activity, MHC class I, type I IFN response, and type II IFN response, were statistically different between the different risk groups (Figure 9B). The expression of ICGs, including CTLA‐4 (*p* < .001), PDCD1 (*p* < .01), LAG3 (*p* < .05), TIGIT (*p* < .05), and CD274 (*p* < .05) among others, was also statistically different between different risk groups (Figure 9C). Overall, our results suggest that patients in the high‐risk group may benefit more from immunotherapy and provide insights into potential targeted therapeutic strategies for HCC patients.

### 3.5 Selection of antitumor drugs

**FIGURE 10:**
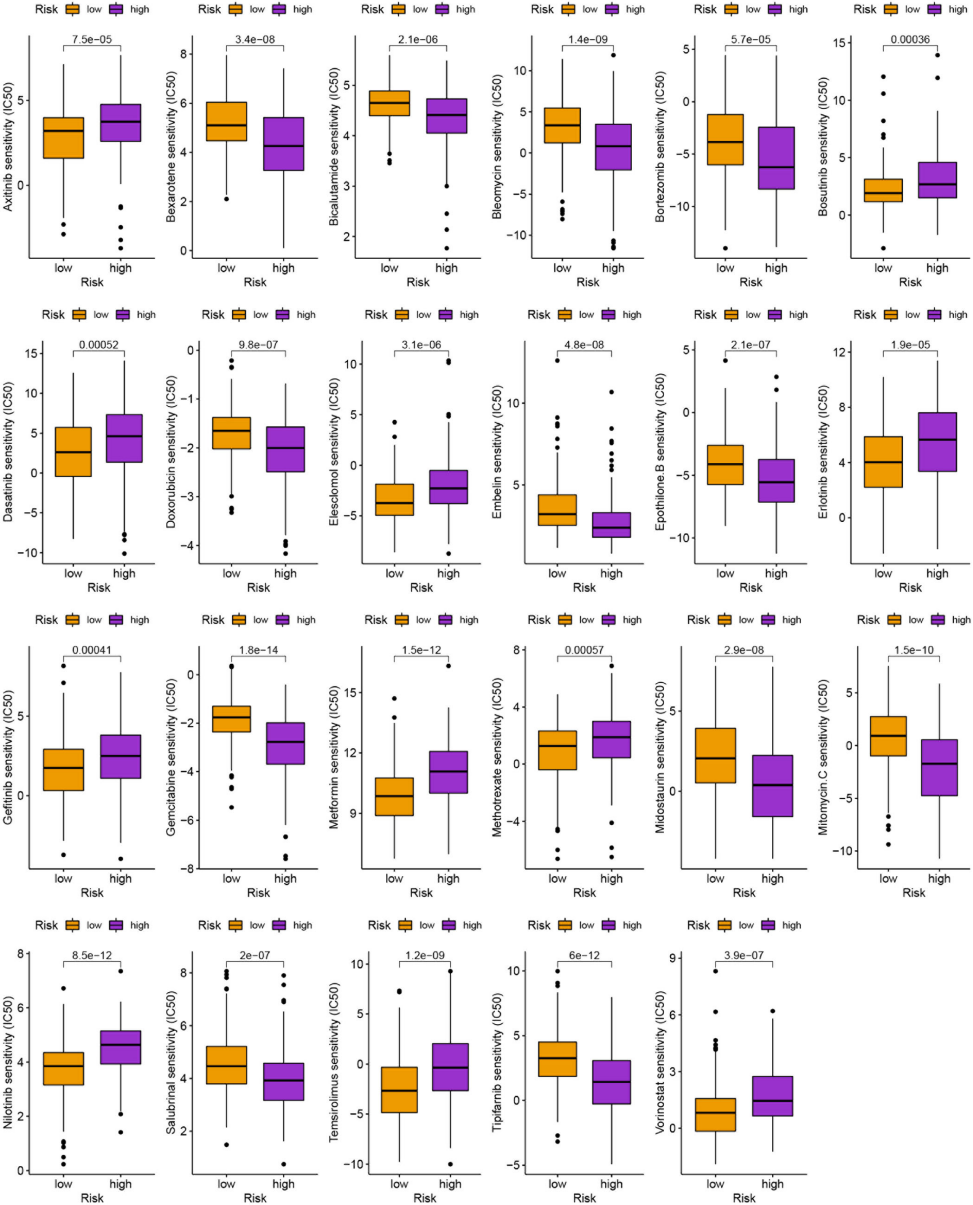
Identification of traditional chemotherapeutic agents.

**FIGURE 11:**
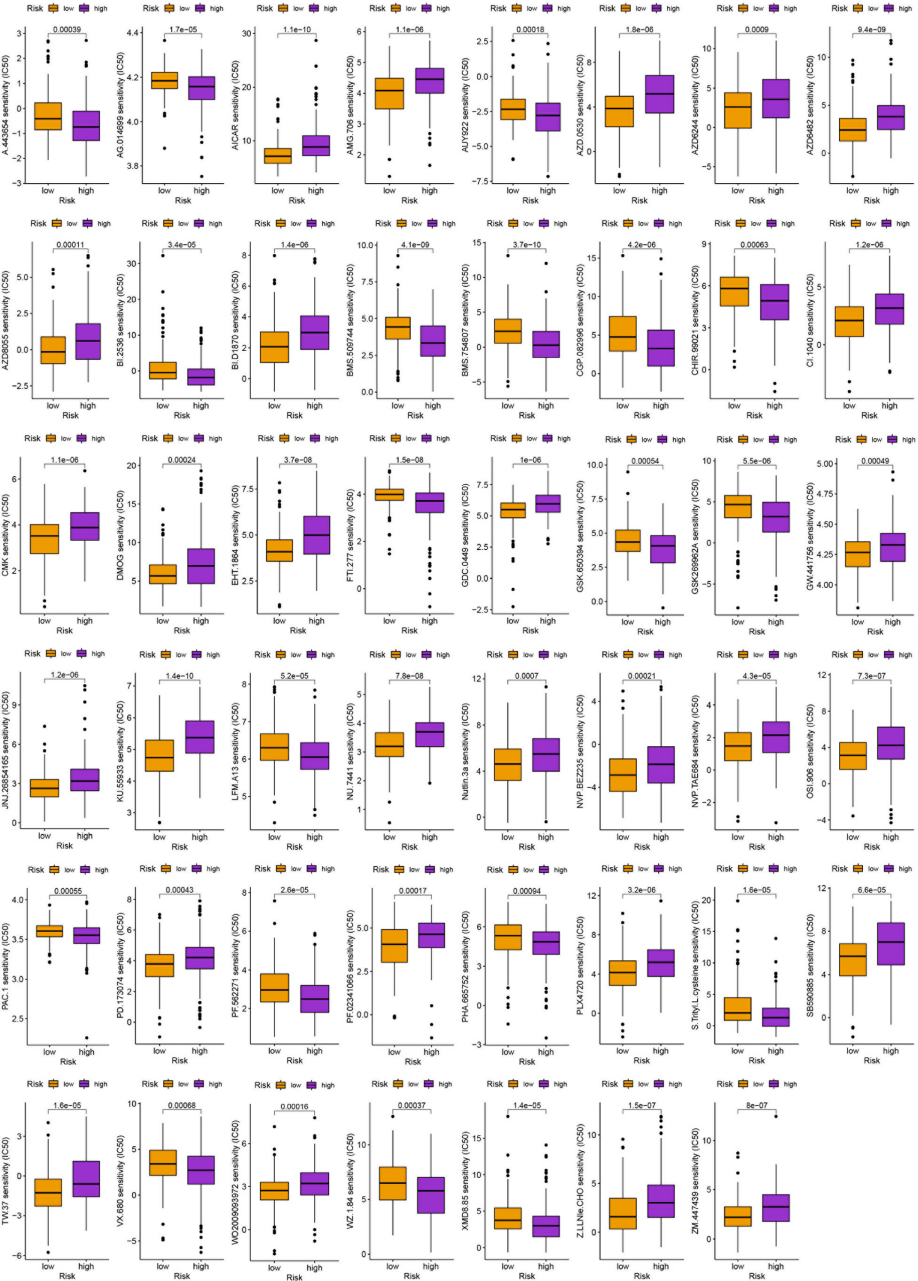
Identification of novel potential compounds.

Besides immunotherapy, we are interested in finding different chemotherapeutic drugs and novel targeted drugs for patients in different risk groups. Finally, we searched for different chemotherapeutic agents and novel targeted agents for patients in different groups, which helped to develop individualized treatment regimens for different individuals (*p* < .001; Figures 10 and 11). This extensive search proved instrumental in devising individualized treatment regimens tailored specifically to the unique characteristics and requirements of different individuals.

### 3.6 Correlation between TRG signature and malignant features

**FIGURE 12:**
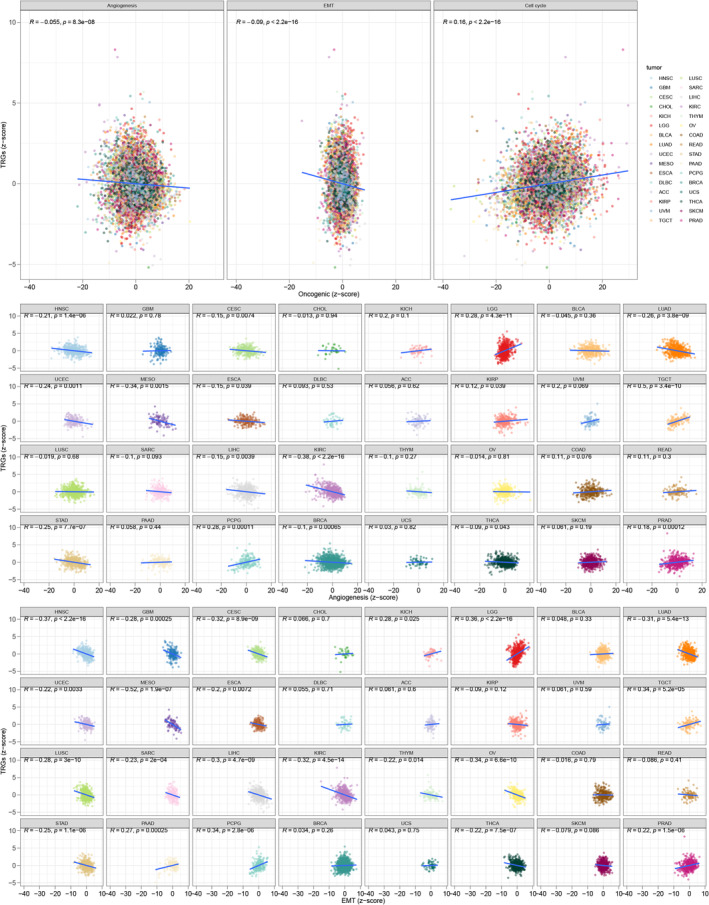
Significant correlations between the TRG z‐score and the angiogenesis *z*‐score (*R* = −0.055, *p* < .001), the EMT *z*‐score (*R* = −0.09, *p* < .001), and the cell cycle *z*‐score (*R* = 0.16, *p* < .001) among the TCGA pan‐cancer patient cohort.

Compared to normal cells, tumor cells possess a unique set of characteristics that enable them to proliferate rapidly, invade surrounding tissues, metastasize to distant organs, activate EMT, and drive angiogenesis.^31^ These malignant traits ultimately culminate in the development of cancer. The role of TRG in inducing cell death is well‐established, and its association with cancer progression and drug resistance has been extensively studied. To examine the relationship between TRGs and the malignant features of tumors, we employed the *z*‐score algorithm to calculate the TRG, angiogenesis, EMT, and cell cycle scores. Our results revealed significant correlations between the TRG *z*‐score and the angiogenesis *z*‐score (*R* = −0.055, *p* < .001), the EMT *z*‐score (*R* = −0.09, *p* < .001), and the cell cycle *z*‐score (*R* = 0.16, *p* < .001) across the TCGA pan‐cancer patient cohort (Figure 12). These findings highlight the potential importance of TRGs as targets for cancer therapy, particularly given their involvement in various malignant processes.

### 3.7 Validation of TRGs

**FIGURE 13:**
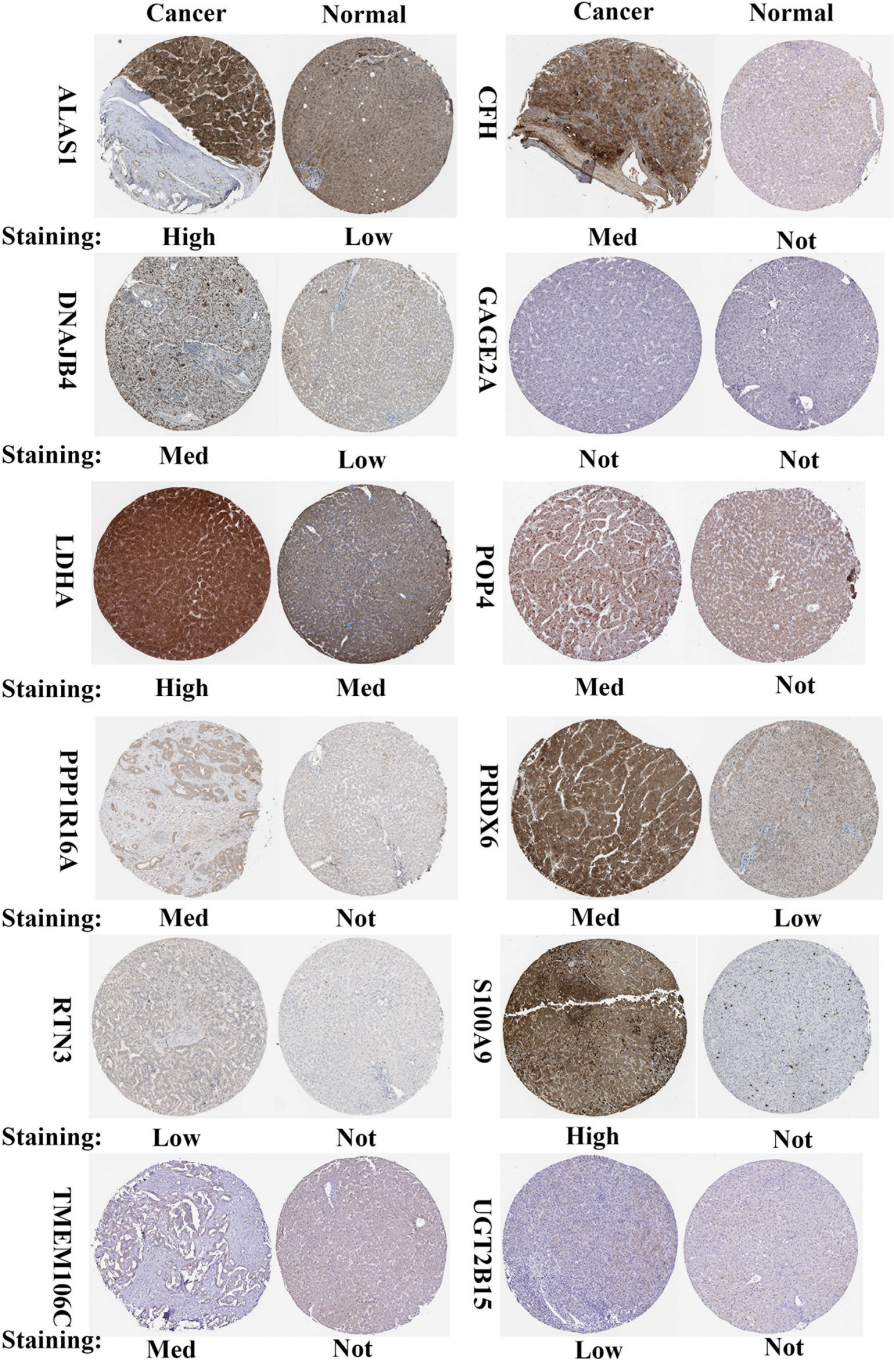
S100A9, CFH, PPP1R16A, POP4, PRDX6, UGT2B15, LDHA, TMEM106C, ALAS1, RTN3, and DNAJB4 were significantly upregulated in HCC tumor tissues compared to normal tissues.

**FIGURE 14:**
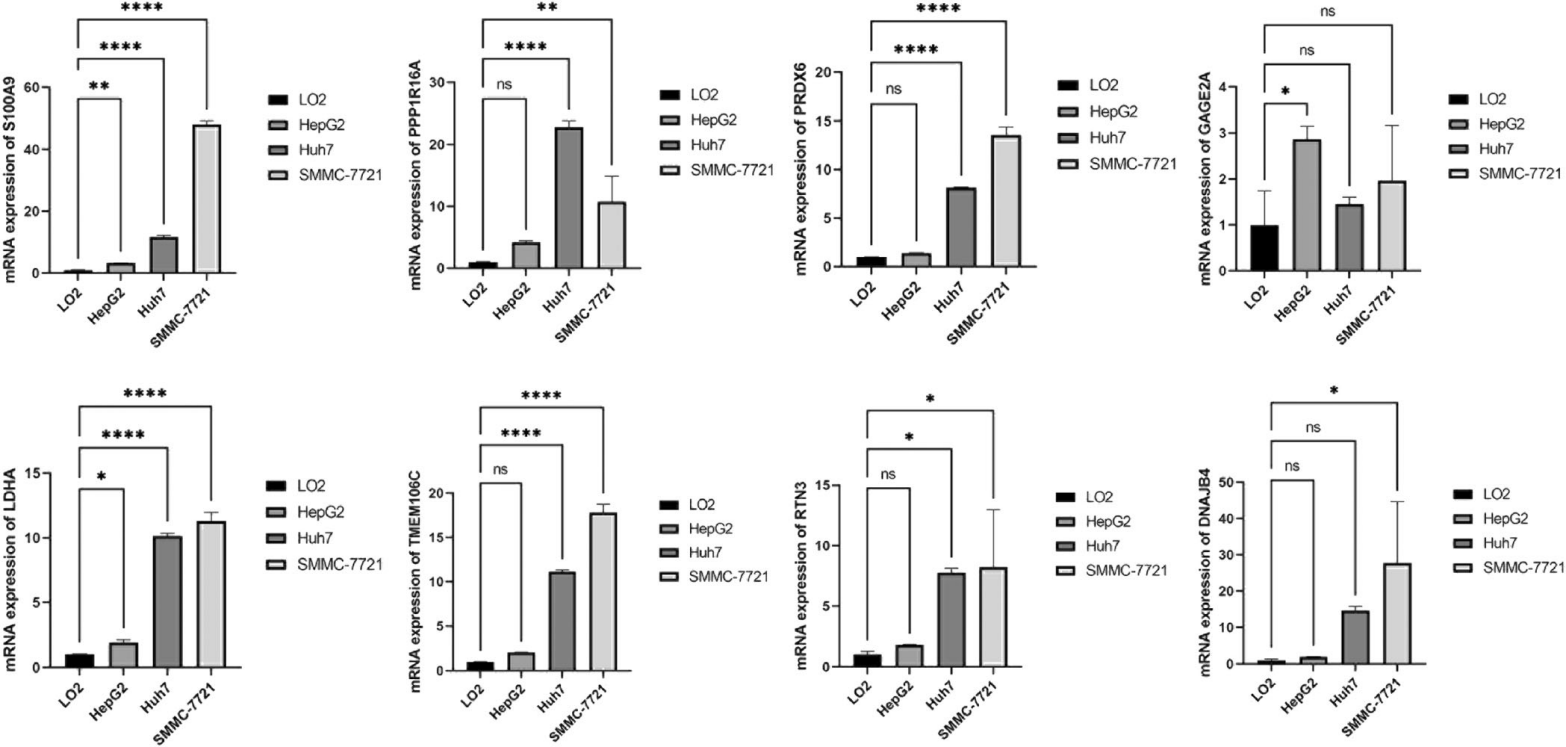
The mRNA expression levels of 8 prognostic TRGs were upregulated in HCC cells compared to normal cells. *p < .05; **p < .01; ***p < .001; and ****p < .0001.

In addition, the study examined protein expression levels in HCC tumor tissues and normal tissues using the HPA database. Interestingly, we found that S100A9, CFH, PPP1R16A, POP4, PRDX6, UGT2B15, LDHA, TMEM106C, ALAS1, RTN3, and DNAJB4 were significantly upregulated in HCC tumor tissues compared to normal tissues (Figure 13). However, the expression levels of GAGE2A did not differ significantly between the two tissue types. To further validate our results, we used qRT‐PCR analysis to verify the mRNA expression levels of eight prognostic TRGs (S100A9, PPP1R16A, PRDX6, GAGE2A, LDHA, TMEM106C, RTN3, and DNAJB4). The results showed that the mRNA expression levels of eight prognostic TRGs were upregulated in cancer cells compared to normal cells (Figure 14). The results of this study have demonstrated a substantial increase in both mRNA and protein expression levels of the genes included in the model within tumor tissues. This observation aligns closely with our previously reported findings and provides further evidence supporting the involvement of these genes in tumor development.

## 4 DISCUSSION

The heterogeneity of HCC poses a significant challenge in predicting treatment responses and prognosis, as patients with similar clinical stages may exhibit different outcomes. Therefore, investigating the relationship between HCC tumor heterogeneity, treatment efficacy, and prognosis is crucial. We employed scRNA‐seq data to identify 594 TRGs, from which we constructed a 12‐TRG prognostic model using TCGA data. We further validated this model using GEO data and confirmed the gene expression using qRT‐PCR. Our findings demonstrate the utility of this prognostic model in accurately forecasting the prognosis of HCC, which can inform the development of tailored treatment regimens based on individual patient factors.

Our analysis of cell communication patterns revealed significant upregulation of FGF, EGFR, VEGFR, and MET‐related ligand–receptor expression in HCC cells compared to other cells. A growing body of evidence has demonstrated that inhibitors targeting FGF/FGFR, including multikinase inhibitors, specialized FGFR4 inhibitors, and FGF ligand traps, exhibit antitumor activity in preclinical or early stages of HCC.^35^ In addition, VEGFR and VEGF inhibitors, including ramucirumab and bevacizumab, have been endorsed as first‐ or second‐line treatments for HCC.^36^ Thus, based on the expression profiles of these ligand receptors, we can develop tailored therapeutic regimens for individual patients to enhance treatment efficacy.

During the construction of the model, we found that most of the genes were related to the development of cancer. High S100A9+ cell density indicated poor prognosis after radical resection in patients with HCC.^37^ S100A9 promotes the growth and invasion of HCC cells via the RAGE‐mediated ERK1/2 and p38 MAPK pathways.^38^ CFH contributes to tumorigenesis and metastasis by inhibiting complement‐dependent cytotoxicity of HCC.^39^ The phospholipase A2 activity of PRDX6 promotes tumor necrosis factor alpha‐induced cancer cell death in HCC.^40^ Increased LDHA expression is associated with poor survival in HCC and can mediate the antitumor effects of gemcitabine.^41^ TMEM106C was overexpressed and inhibition of TMEM106C remarkably inhibited HCC proliferation and metastasis by targeting CENPM and DLC‐1.^42^ RTN3 expression was downregulated in HCC, which is different from our findings.^43^ The remaining genes are also closely associated with the progression of other tumors.^44^, ^45^, ^46^, ^47^, ^48^, ^49^ The above‐mentioned studies are generally consistent with the results of our study, indicating the reliability and replicability of our results. Another study based on single‐nucleus RNA sequencing identified a previously identified and uncharacterized type of disease‐associated hepatocyte that is becoming more prevalent with the progression of chronic liver disease.^50^ These identified DEGs were highly correlated with the list of TRGs in this study.

Although the study has yielded promising results, it is important to acknowledge some drawbacks and limitations. One potential limitation is that the majority of samples used in the study were non‐metastatic, which may result in some bias in the analysis. Additionally, while the expression levels of genes have been validated, there is still a lack of specific functional experiments on these genes. Lastly, although the TCGA and GEO databases provide a relatively well‐documented profile of clinical features, there are some specific clinical features, such as hepatitis B virus infection and alcohol abuse, that are not recorded. To address these limitations, future studies should aim to increase the number of clinical samples and incorporate more complete and specific clinical records to further validate the findings of this research.

## 5 CONCLUSION

Overall, the study sheds light on the crucial role of heterogeneity in tumor cells in shaping the prognosis and therapeutic management of HCC. The findings of this study have the potential to augment the predictive accuracy of the existing TNM staging system and offer new avenues for prognostic evaluation and treatment optimization for HCC patients. Accordingly, these findings may inform the development of more effective precision medicine strategies that account for the unique complexities of HCC and improve patient outcomes.

## AUTHOR CONTRIBUTIONS

All authors had full access to the data in the study and took responsibility for the integrity of the data and the accuracy of the data analysis. Conceptualization, Yaping Mu and Jing Wang; Methodology, Xiaodong Wang and Yurong Zhang; Investigation, Ding Zheng and Qinghua Peng; Formal Analysis, Fei Ye and Jing Wang; Resources, Yue Yin, and Encheng Wang; Writing – Original Draft, Yaping Mu; Writing – Review & Editing, Jing Wang; Supervision, Jing Wang; Funding Acquisition, Jing Wang and Ding Zheng.

## FUNDING INFORMATION

This study was supported by Luzhou “Jiucheng Talents·Scientific and Technological Innovation Team” (Number: [2021] No. 162), TCM talent growth platform construction project of Sichuan Provincial Administration of Traditional Chinese Medicine, National Traditional Chinese Medicine Clinical Research Base Construction Unit Research Project of the Affiliated Traditional Chinese Medicine Hospital, Southwest Medical University (Number: [2020] No. 33), and Joint project of Southwest Medical University‐Affiliated Traditional Chinese Medicine Hospital of Southwest Medical University (Number: [2020] No. 6, 2020XYLH‐037), Basic Clinical Research on the Prevention and Treatment of Primary Liver Cancer Based on the Academic Thought of Professor Sun Tongjiao, a National Famous Traditional Chinese Medicine (Number: [2022] 2022YFS0619).

## CONFLICT OF INTEREST STATEMENT

The authors declare that there is no conflict of interest regarding the publication of this article.

## ETHICS STATEMENT

All information used in this study came from a publicly available database or a published source that was properly cited. It is therefore not subject to ethical approval and informed consent is not required.

## Supporting information


**Table S1.** The marker genes.
**Table S2.** The lists of TRGs.
**Table S3.** The 289 prognostic TRGs by univariate Cox analysis.
**Table S4.** The details of GO enrichment analysis.
**Table S5.** The details of KEGG enrichment analysis.
**Table S6.** The details of immune cell landscape.

## Data Availability

The data used to support the findings of this study are included in the Supporting Information files.
