# Supplementary material for: Integration of single‐cell and bulk RNA‐sequencing to analyze the heterogeneity of hepatocellular carcinoma and establish a prognostic model
Source: Cancer Rep (Hoboken). 2023 Nov 22;7(1):e1935. doi: 10.1002/cnr2.1935 (PMC10809200; doi:10.1002/cnr2.1935)
Supplement: Supplementary file 1 — Table S1. The marker genes. Table S2. The lists of TRGs. Table S3. The 289 prognostic TRGs by univariate Cox analysis. Table S4. The details of GO enrichment analysis. Table S5. The details of KEGG enrichment analysis. Table S6. The details of immune cell landscape. [file CNR2-7-e1935-s001.docx]

Tab S1. The marker genes.

| Celltype | Markergene |
| --- | --- |
| Fibroblast | COL1A2 |
|  | COL1A1 |
|  | ACTA2 |
| Endothelial | FCGR2B |
|  | PECAM1 |
|  | VWF |
| B | CD79A |
|  | JCHAIN |
|  | IGHG1 |
| Myeloid | KIT |
|  | CLEC4C |
|  | CD1C |
|  | CD163 |
|  | CD14 |
|  | CD68 |
| T/NK | NKG7 |
|  | CD3E |
|  | CD3D |
| Hepatocyte | EPCAM |
|  | HNF4A |
|  | SERPINA1 |
|  | ALB |

Tab S2. The lists of TRGs.

| gene | p_val | avg_log2FC | pct.1 | pct.2 | p_val_adj |
| --- | --- | --- | --- | --- | --- |
| ARMC1 | 0 | 0.250029007 | 0.577 | 0.2 | 0 |
| TTPA | 1.31E-243 | 0.250293626 | 0.479 | 0.176 | 3.33E-239 |
| HNRNPA3 | 1.02E-127 | 0.250491066 | 0.914 | 0.657 | 2.60E-123 |
| C8orf33 | 4.85E-244 | 0.250977948 | 0.699 | 0.328 | 1.24E-239 |
| OAZ1 | 2.41E-73 | 0.251624451 | 0.967 | 0.901 | 6.15E-69 |
| SNAPIN | 1.92E-261 | 0.252189678 | 0.687 | 0.319 | 4.89E-257 |
| TNIP1 | 3.05E-201 | 0.252268264 | 0.633 | 0.313 | 7.78E-197 |
| BTF3 | 6.78E-96 | 0.25230116 | 0.997 | 0.907 | 1.73E-91 |
| RPS9 | 3.15E-158 | 0.252397872 | 0.999 | 0.98 | 8.03E-154 |
| SNX17 | 4.75E-198 | 0.252778533 | 0.742 | 0.398 | 1.21E-193 |
| GLMP | 9.49E-179 | 0.252960882 | 0.795 | 0.467 | 2.42E-174 |
| SPARCL1 | 1.53E-299 | 0.253379247 | 0.284 | 0.043 | 3.90E-295 |
| PSMC2 | 6.19E-197 | 0.253502782 | 0.787 | 0.449 | 1.58E-192 |
| SLC25A39 | 5.07E-115 | 0.253658962 | 0.871 | 0.603 | 1.29E-110 |
| RSL1D1 | 8.60E-254 | 0.253785053 | 0.807 | 0.408 | 2.19E-249 |
| CD163 | 4.89E-230 | 0.253930963 | 0.265 | 0.055 | 1.25E-225 |
| AMT | 0 | 0.254024157 | 0.461 | 0.088 | 0 |
| RPL27A | 1.78E-188 | 0.254089633 | 1 | 0.982 | 4.54E-184 |
| H1F0 | 5.54E-139 | 0.254408805 | 0.735 | 0.422 | 1.41E-134 |
| MEAF6 | 1.65E-212 | 0.254766361 | 0.843 | 0.478 | 4.21E-208 |
| EEF2 | 5.11E-159 | 0.255056167 | 0.987 | 0.802 | 1.30E-154 |
| DNAJC7 | 3.38E-250 | 0.255095937 | 0.799 | 0.405 | 8.60E-246 |
| CCDC47 | 3.38E-199 | 0.25571581 | 0.817 | 0.459 | 8.61E-195 |
| PCNA | 3.49E-178 | 0.255729756 | 0.619 | 0.303 | 8.90E-174 |
| H3F3A | 2.67E-53 | 0.255893861 | 0.997 | 0.962 | 6.80E-49 |
| SNRPC | 3.78E-184 | 0.255938787 | 0.947 | 0.637 | 9.63E-180 |
| MAGOH | 5.14E-205 | 0.256250031 | 0.862 | 0.511 | 1.31E-200 |
| COA7 | 0 | 0.256354952 | 0.472 | 0.101 | 0 |
| COMMD9 | 0 | 0.256855064 | 0.592 | 0.195 | 0 |
| TPD52L1 | 1.75E-299 | 0.257356255 | 0.605 | 0.236 | 4.47E-295 |
| ACSM2A | 2.16E-85 | 0.259183573 | 0.569 | 0.355 | 5.51E-81 |
| RARS | 1.29E-292 | 0.259577657 | 0.635 | 0.259 | 3.29E-288 |
| HNRNPK | 1.49E-128 | 0.260291694 | 0.968 | 0.786 | 3.80E-124 |
| PLPP1 | 2.55E-242 | 0.260432572 | 0.5 | 0.194 | 6.49E-238 |
| ISCA1 | 2.43E-260 | 0.260503369 | 0.754 | 0.368 | 6.20E-256 |
| CALU | 8.11E-216 | 0.260547933 | 0.703 | 0.355 | 2.07E-211 |
| FAM32A | 4.84E-295 | 0.260766348 | 0.653 | 0.271 | 1.23E-290 |
| MMP1 | 1.64E-240 | 0.260872256 | 0.237 | 0.036 | 4.18E-236 |
| APH1A | 9.41E-176 | 0.261077636 | 0.817 | 0.498 | 2.40E-171 |
| C9orf16 | 9.35E-203 | 0.261079117 | 0.811 | 0.467 | 2.38E-198 |
| RPL18A | 2.14E-26 | 0.261742569 | 1 | 0.991 | 5.46E-22 |
| PLOD1 | 6.51E-191 | 0.261786238 | 0.705 | 0.367 | 1.66E-186 |
| PPP1R1A | 2.09E-103 | 0.262195047 | 0.39 | 0.212 | 5.32E-99 |
| PSMB8 | 5.43E-213 | 0.262318125 | 0.81 | 0.447 | 1.38E-208 |
| MRPL21 | 1.19E-276 | 0.262325668 | 0.804 | 0.409 | 3.04E-272 |
| SSU72 | 1.92E-163 | 0.26240039 | 0.815 | 0.503 | 4.89E-159 |
| RPL12 | 5.54E-89 | 0.262408229 | 0.999 | 0.97 | 1.41E-84 |
| LMO4 | 5.50E-236 | 0.262613252 | 0.556 | 0.236 | 1.40E-231 |
| RPS6KB2 | 0 | 0.263136021 | 0.63 | 0.226 | 0 |
| HSD3B7 | 3.30E-222 | 0.263306003 | 0.54 | 0.229 | 8.41E-218 |
| PTGES3 | 3.21E-111 | 0.263406803 | 0.987 | 0.833 | 8.17E-107 |
| RTCA | 0 | 0.263658201 | 0.582 | 0.194 | 0 |
| PDLIM3 | 0 | 0.264604887 | 0.333 | 0.054 | 0 |
| AURKAIP1 | 8.86E-103 | 0.264834857 | 0.938 | 0.75 | 2.26E-98 |
| CYP2A6 | 6.22E-54 | 0.265143793 | 0.254 | 0.137 | 1.59E-49 |
| RPL9 | 5.36E-205 | 0.265472908 | 1 | 0.973 | 1.36E-200 |
| NABP2 | 0 | 0.265697613 | 0.63 | 0.235 | 0 |
| PABPC1 | 4.63E-275 | 0.265776809 | 0.991 | 0.777 | 1.18E-270 |
| SSR3 | 5.05E-120 | 0.26580697 | 0.954 | 0.708 | 1.29E-115 |
| ATF5 | 1.09E-166 | 0.266398822 | 0.494 | 0.224 | 2.77E-162 |
| HSD17B14 | 1.95E-218 | 0.267322385 | 0.387 | 0.132 | 4.96E-214 |
| FASTK | 1.04E-210 | 0.26745841 | 0.725 | 0.388 | 2.65E-206 |
| RHOD | 9.16E-299 | 0.268089838 | 0.485 | 0.153 | 2.33E-294 |
| PSMD13 | 5.57E-286 | 0.268172834 | 0.762 | 0.357 | 1.42E-281 |
| RTFDC1 | 2.11E-265 | 0.269328366 | 0.799 | 0.41 | 5.37E-261 |
| CTAG2 | 3.15E-239 | 0.269909738 | 0.466 | 0.162 | 8.03E-235 |
| TPR | 2.33E-227 | 0.269925964 | 0.703 | 0.351 | 5.94E-223 |
| TRUB2 | 0 | 0.270206448 | 0.599 | 0.191 | 0 |
| PRCC | 0 | 0.270295433 | 0.611 | 0.23 | 0 |
| DIO1 | 3.99E-79 | 0.270404104 | 0.65 | 0.453 | 1.02E-74 |
| RAB2A | 5.18E-111 | 0.270876855 | 0.917 | 0.706 | 1.32E-106 |
| DNAJB4 | 1.90E-272 | 0.271444384 | 0.533 | 0.195 | 4.84E-268 |
| MRPL13 | 6.49E-212 | 0.271881908 | 0.925 | 0.585 | 1.65E-207 |
| SSBP1 | 5.82E-143 | 0.271921766 | 0.927 | 0.704 | 1.48E-138 |
| VPS25 | 1.01E-199 | 0.271961499 | 0.803 | 0.45 | 2.57E-195 |
| C17orf58 | 3.79E-299 | 0.272124838 | 0.469 | 0.148 | 9.65E-295 |
| CCT8 | 3.20E-186 | 0.272141862 | 0.877 | 0.545 | 8.15E-182 |
| SKAP1 | 3.42E-168 | 0.272468941 | 0.262 | 0.08 | 8.71E-164 |
| PYCARD | 5.06E-145 | 0.272481446 | 0.571 | 0.314 | 1.29E-140 |
| RRS1 | 1.19E-273 | 0.272612251 | 0.416 | 0.126 | 3.04E-269 |
| SPTSSA | 3.42E-154 | 0.272868187 | 0.829 | 0.541 | 8.72E-150 |
| APRT | 1.46E-159 | 0.272940672 | 0.94 | 0.666 | 3.72E-155 |
| RPL22 | 8.99E-135 | 0.273623336 | 1 | 0.963 | 2.29E-130 |
| HLA-DPA1 | 4.57E-113 | 0.273632336 | 0.707 | 0.476 | 1.17E-108 |
| MRPS12 | 3.95E-206 | 0.273739804 | 0.868 | 0.525 | 1.01E-201 |
| EPHX1 | 5.38E-79 | 0.273972711 | 0.993 | 0.831 | 1.37E-74 |
| RPL13A | 6.45E-135 | 0.274181927 | 1 | 0.996 | 1.64E-130 |
| CCDC34 | 0 | 0.274396371 | 0.556 | 0.197 | 0 |
| TACO1 | 2.34E-231 | 0.274697639 | 0.538 | 0.234 | 5.95E-227 |
| IMP4 | 1.90E-259 | 0.27508108 | 0.753 | 0.38 | 4.85E-255 |
| EPB41L2 | 1.15E-301 | 0.275107504 | 0.42 | 0.117 | 2.92E-297 |
| ATP6V1C1 | 7.49E-262 | 0.275206317 | 0.65 | 0.288 | 1.91E-257 |
| TIMP1 | 7.82E-48 | 0.275283081 | 0.605 | 0.425 | 1.99E-43 |
| PSMD12 | 4.38E-255 | 0.275428715 | 0.755 | 0.371 | 1.12E-250 |
| CLTA | 5.59E-96 | 0.275996492 | 0.817 | 0.639 | 1.42E-91 |
| REPIN1 | 1.71E-173 | 0.276001143 | 0.56 | 0.286 | 4.36E-169 |
| TMEM126A | 0 | 0.276757849 | 0.742 | 0.341 | 0 |
| ASPSCR1 | 2.93E-249 | 0.27690519 | 0.584 | 0.251 | 7.48E-245 |
| ENAH | 1.48E-151 | 0.276934666 | 0.605 | 0.322 | 3.78E-147 |
| DHFR | 1.04E-297 | 0.277457568 | 0.451 | 0.136 | 2.64E-293 |
| GSTA1 | 3.58E-65 | 0.278207533 | 0.937 | 0.769 | 9.13E-61 |
| COMMD5 | 0 | 0.278257579 | 0.629 | 0.214 | 0 |
| METTL23 | 7.16E-201 | 0.278303775 | 0.7 | 0.37 | 1.82E-196 |
| SMYD2 | 0 | 0.278393406 | 0.505 | 0.158 | 0 |
| NRDC | 0 | 0.278803138 | 0.698 | 0.287 | 0 |
| SCAMP3 | 1.17E-286 | 0.279803185 | 0.696 | 0.313 | 2.99E-282 |
| RPS20 | 6.34E-103 | 0.280008888 | 1 | 0.99 | 1.62E-98 |
| TRAPPC3 | 5.52E-235 | 0.280116789 | 0.806 | 0.442 | 1.41E-230 |
| POLR2K | 2.34E-162 | 0.280234595 | 0.979 | 0.74 | 5.95E-158 |
| VMP1 | 1.34E-167 | 0.281019367 | 0.906 | 0.615 | 3.41E-163 |
| PIR | 7.76E-300 | 0.282452669 | 0.626 | 0.255 | 1.98E-295 |
| HSPA5 | 1.62E-86 | 0.282792927 | 0.944 | 0.775 | 4.13E-82 |
| FAU | 7.29E-241 | 0.28328246 | 1 | 0.976 | 1.86E-236 |
| NDUFV1 | 4.46E-256 | 0.283847204 | 0.863 | 0.499 | 1.14E-251 |
| PARK7 | 3.23E-160 | 0.284492574 | 0.99 | 0.854 | 8.23E-156 |
| FAM114A1 | 0 | 0.285113159 | 0.637 | 0.236 | 0 |
| SLC25A5 | 1.82E-95 | 0.285455048 | 0.99 | 0.88 | 4.63E-91 |
| RPL29 | 7.31E-55 | 0.285592772 | 0.998 | 0.975 | 1.86E-50 |
| TK1 | 7.03E-258 | 0.286072651 | 0.38 | 0.105 | 1.79E-253 |
| BCAS2 | 6.15E-307 | 0.286108889 | 0.758 | 0.356 | 1.57E-302 |
| TERF1 | 0 | 0.286239739 | 0.602 | 0.208 | 0 |
| NDUFAF3 | 5.00E-184 | 0.286317816 | 0.899 | 0.612 | 1.28E-179 |
| TMEM126B | 0 | 0.2863767 | 0.689 | 0.291 | 0 |
| SHARPIN | 7.14E-213 | 0.286857272 | 0.683 | 0.364 | 1.82E-208 |
| TXNRD1 | 3.08E-169 | 0.286987344 | 0.646 | 0.349 | 7.85E-165 |
| LACTB2 | 0 | 0.287487788 | 0.774 | 0.347 | 0 |
| EMC2 | 0 | 0.287555444 | 0.734 | 0.327 | 0 |
| IGKC | 1.99E-57 | 0.288304017 | 0.617 | 0.804 | 5.07E-53 |
| SLC22A7 | 2.92E-119 | 0.28839403 | 0.425 | 0.217 | 7.45E-115 |
| MRPS15 | 1.47E-182 | 0.288685977 | 0.926 | 0.632 | 3.75E-178 |
| DYNLRB1 | 7.49E-220 | 0.289076161 | 0.942 | 0.624 | 1.91E-215 |
| IFI35 | 4.45E-282 | 0.289294667 | 0.714 | 0.327 | 1.13E-277 |
| SF3B2 | 2.66E-267 | 0.290038531 | 0.816 | 0.432 | 6.79E-263 |
| RPS3A | 1.09E-118 | 0.290162477 | 0.999 | 0.968 | 2.78E-114 |
| DDX49 | 0 | 0.290586977 | 0.598 | 0.22 | 0 |
| TMEM70 | 9.16E-243 | 0.2906624 | 0.666 | 0.318 | 2.33E-238 |
| RPS16 | 1.17E-180 | 0.290673503 | 1 | 0.985 | 2.99E-176 |
| PIPOX | 1.32E-194 | 0.290806903 | 0.652 | 0.344 | 3.37E-190 |
| NRBP1 | 1.09E-272 | 0.291115205 | 0.741 | 0.352 | 2.78E-268 |
| LAP3 | 6.63E-197 | 0.291310564 | 0.714 | 0.385 | 1.69E-192 |
| CHCHD3 | 1.47E-272 | 0.292556322 | 0.664 | 0.303 | 3.74E-268 |
| AK4 | 3.48E-185 | 0.292876622 | 0.735 | 0.43 | 8.86E-181 |
| ASB9 | 0 | 0.2929789 | 0.421 | 0.093 | 0 |
| TMEM98 | 1.60E-277 | 0.293569935 | 0.608 | 0.254 | 4.07E-273 |
| EPRS | 0 | 0.293728295 | 0.623 | 0.243 | 0 |
| SLC35B1 | 4.89E-251 | 0.294092027 | 0.787 | 0.415 | 1.25E-246 |
| PSMD2 | 4.57E-206 | 0.294279315 | 0.809 | 0.469 | 1.16E-201 |
| MINCR | 2.23E-296 | 0.29459941 | 0.396 | 0.106 | 5.69E-292 |
| FIBP | 0 | 0.294844531 | 0.737 | 0.333 | 0 |
| NQO2 | 6.64E-161 | 0.294945416 | 0.824 | 0.529 | 1.69E-156 |
| SSR2 | 6.23E-116 | 0.295406317 | 0.939 | 0.732 | 1.59E-111 |
| UBXN4 | 4.00E-207 | 0.295484136 | 0.853 | 0.527 | 1.02E-202 |
| CMSS1 | 2.48E-243 | 0.296428507 | 0.663 | 0.311 | 6.32E-239 |
| SSNA1 | 3.36E-288 | 0.296504734 | 0.782 | 0.386 | 8.57E-284 |
| MT-ND1 | 4.49E-69 | 0.296519472 | 0.995 | 0.884 | 1.14E-64 |
| ABCG2 | 0 | 0.296798056 | 0.347 | 0.057 | 0 |
| ATP6V0E1 | 8.61E-116 | 0.296924156 | 0.983 | 0.8 | 2.19E-111 |
| PRSS3 | 4.03E-161 | 0.297622339 | 0.215 | 0.051 | 1.03E-156 |
| PTDSS1 | 0 | 0.297803368 | 0.576 | 0.192 | 0 |
| MRPL11 | 0 | 0.298198481 | 0.788 | 0.362 | 0 |
| UBE2V2 | 1.56E-290 | 0.298954881 | 0.887 | 0.495 | 3.97E-286 |
| ATP5B | 2.18E-118 | 0.299108528 | 0.986 | 0.865 | 5.56E-114 |
| MRPL45 | 0 | 0.299240107 | 0.592 | 0.221 | 0 |
| RPN2 | 9.01E-125 | 0.299916172 | 0.926 | 0.672 | 2.30E-120 |
| TIMM8B | 2.55E-227 | 0.300098267 | 0.925 | 0.628 | 6.49E-223 |
| PSMA5 | 2.06E-191 | 0.30096662 | 0.898 | 0.584 | 5.26E-187 |
| NDUFB5 | 1.04E-149 | 0.303763596 | 0.94 | 0.686 | 2.65E-145 |
| HSPD1 | 1.39E-120 | 0.304246626 | 0.993 | 0.875 | 3.55E-116 |
| C1orf21 | 0 | 0.304323374 | 0.433 | 0.108 | 0 |
| APOA1BP | 2.37E-173 | 0.305245131 | 0.851 | 0.556 | 6.03E-169 |
| CACYBP | 2.93E-241 | 0.305724932 | 0.904 | 0.557 | 7.46E-237 |
| EMP2 | 6.26E-285 | 0.30578546 | 0.697 | 0.326 | 1.59E-280 |
| PSMC3 | 3.36E-234 | 0.306224706 | 0.873 | 0.535 | 8.56E-230 |
| EIF3M | 2.96E-258 | 0.306593933 | 0.858 | 0.481 | 7.54E-254 |
| SDHC | 6.32E-89 | 0.307276069 | 0.928 | 0.751 | 1.61E-84 |
| PPA2 | 1.29E-230 | 0.30746949 | 0.77 | 0.417 | 3.29E-226 |
| TMX2 | 7.41E-305 | 0.307562755 | 0.723 | 0.325 | 1.89E-300 |
| ACSM1 | 0 | 0.307765923 | 0.403 | 0.081 | 0 |
| ASPH | 5.39E-206 | 0.308427433 | 0.828 | 0.47 | 1.37E-201 |
| C19orf12 | 4.26E-232 | 0.30881129 | 0.479 | 0.191 | 1.08E-227 |
| COX5B | 4.33E-196 | 0.308893241 | 0.995 | 0.914 | 1.10E-191 |
| ATP6V0B | 1.12E-145 | 0.309398101 | 0.981 | 0.789 | 2.85E-141 |
| FDFT1 | 5.34E-204 | 0.309547467 | 0.704 | 0.375 | 1.36E-199 |
| TIPRL | 5.18E-230 | 0.309818642 | 0.684 | 0.358 | 1.32E-225 |
| MRPL15 | 0 | 0.309957383 | 0.761 | 0.353 | 0 |
| SLC50A1 | 6.44E-236 | 0.310560788 | 0.769 | 0.403 | 1.64E-231 |
| SVIP | 4.34E-249 | 0.310655042 | 0.746 | 0.392 | 1.11E-244 |
| NSA2 | 1.34E-180 | 0.311026026 | 0.749 | 0.434 | 3.40E-176 |
| NACA | 1.02E-206 | 0.311162575 | 1 | 0.965 | 2.61E-202 |
| NDUFA8 | 0 | 0.311324215 | 0.846 | 0.44 | 0 |
| ZFAND5 | 2.26E-121 | 0.311541228 | 0.808 | 0.531 | 5.77E-117 |
| OPTN | 6.06E-239 | 0.311570562 | 0.749 | 0.392 | 1.54E-234 |
| GALK1 | 1.40E-140 | 0.311837233 | 0.876 | 0.611 | 3.57E-136 |
| RPS8 | 1.19E-120 | 0.311943274 | 1 | 0.992 | 3.04E-116 |
| KXD1 | 3.51E-255 | 0.31205684 | 0.642 | 0.294 | 8.95E-251 |
| MRPL37 | 3.66E-206 | 0.31211228 | 0.822 | 0.479 | 9.33E-202 |
| GNB2L1 | 4.19E-36 | 0.312271689 | 0.997 | 0.951 | 1.07E-31 |
| NDUFS2 | 3.51E-124 | 0.312475336 | 0.903 | 0.668 | 8.94E-120 |
| SEPP1 | 9.44E-27 | 0.312660022 | 0.987 | 0.893 | 2.40E-22 |
| LAMTOR2 | 5.31E-201 | 0.312957275 | 0.949 | 0.72 | 1.35E-196 |
| BIRC5 | 5.87E-155 | 0.313285986 | 0.391 | 0.164 | 1.49E-150 |
| TKFC | 0 | 0.313303248 | 0.55 | 0.196 | 0 |
| RPL35A | 6.68E-108 | 0.314252662 | 1 | 0.988 | 1.70E-103 |
| ZFAS1 | 1.37E-143 | 0.314399748 | 0.835 | 0.523 | 3.49E-139 |
| ADIPOR1 | 3.12E-155 | 0.314472359 | 0.695 | 0.413 | 7.94E-151 |
| ZDHHC12 | 0 | 0.314753894 | 0.676 | 0.259 | 0 |
| PRPSAP1 | 6.74E-254 | 0.314892785 | 0.643 | 0.304 | 1.72E-249 |
| PGLS | 1.33E-76 | 0.31509279 | 0.552 | 0.382 | 3.38E-72 |
| NDUFC2 | 1.49E-179 | 0.31638498 | 0.986 | 0.867 | 3.78E-175 |
| RTN3 | 7.65E-223 | 0.316913487 | 0.842 | 0.487 | 1.95E-218 |
| PPIE | 0 | 0.31722759 | 0.66 | 0.207 | 0 |
| MFSD3 | 1.86E-286 | 0.317399568 | 0.488 | 0.175 | 4.75E-282 |
| LAMTOR1 | 1.65E-258 | 0.317484406 | 0.87 | 0.516 | 4.20E-254 |
| CYB5A | 1.31E-113 | 0.317529214 | 0.993 | 0.884 | 3.33E-109 |
| POLE4 | 1.43E-228 | 0.317829893 | 0.773 | 0.428 | 3.65E-224 |
| SCP2 | 4.04E-156 | 0.318586808 | 0.972 | 0.836 | 1.03E-151 |
| FAM133A | 0 | 0.318624045 | 0.351 | 0.025 | 0 |
| RAB8A | 0 | 0.318682529 | 0.655 | 0.232 | 0 |
| BIRC3 | 4.00E-141 | 0.319573654 | 0.571 | 0.307 | 1.02E-136 |
| ALAS1 | 9.22E-292 | 0.32018064 | 0.717 | 0.332 | 2.35E-287 |
| TOR1AIP2 | 9.63E-168 | 0.320568849 | 0.772 | 0.468 | 2.45E-163 |
| SDCBP | 1.08E-235 | 0.320681558 | 0.952 | 0.67 | 2.75E-231 |
| CNN3 | 1.67E-179 | 0.320895978 | 0.92 | 0.653 | 4.25E-175 |
| UGT2B17 | 0 | 0.321214293 | 0.305 | 0.047 | 0 |
| SNHG9 | 1.69E-205 | 0.321635213 | 0.576 | 0.285 | 4.30E-201 |
| AAMDC | 2.54E-249 | 0.321776639 | 0.707 | 0.351 | 6.48E-245 |
| NDUFA2 | 1.31E-106 | 0.322351391 | 0.968 | 0.807 | 3.33E-102 |
| NDUFB4 | 3.04E-191 | 0.322498037 | 0.988 | 0.838 | 7.75E-187 |
| C6 | 2.36E-120 | 0.323129015 | 0.384 | 0.185 | 6.00E-116 |
| CEBPD | 1.87E-87 | 0.32418295 | 0.753 | 0.536 | 4.76E-83 |
| PTTG1 | 1.01E-147 | 0.324429856 | 0.49 | 0.235 | 2.57E-143 |
| FTL | 1.31E-79 | 0.325613887 | 1 | 1 | 3.34E-75 |
| SURF1 | 1.79E-168 | 0.327032753 | 0.641 | 0.394 | 4.56E-164 |
| UGDH | 5.26E-96 | 0.327688877 | 0.91 | 0.686 | 1.34E-91 |
| CFI | 5.97E-133 | 0.327736805 | 0.698 | 0.432 | 1.52E-128 |
| RPLP2 | 3.59E-261 | 0.327742032 | 1 | 0.99 | 9.15E-257 |
| PRRC2C | 2.32E-232 | 0.327757347 | 0.76 | 0.422 | 5.92E-228 |
| HINT2 | 3.57E-155 | 0.328655949 | 0.817 | 0.598 | 9.09E-151 |
| SPINK1 | 1.50E-12 | 0.329157949 | 0.588 | 0.634 | 3.81E-08 |
| CERS2 | 4.50E-145 | 0.32921148 | 0.803 | 0.54 | 1.15E-140 |
| RPL24 | 9.59E-152 | 0.329940174 | 1 | 0.959 | 2.44E-147 |
| OXLD1 | 2.34E-288 | 0.330363635 | 0.527 | 0.196 | 5.96E-284 |
| USP1 | 0 | 0.330465261 | 0.534 | 0.186 | 0 |
| DUSP23 | 2.18E-99 | 0.330709665 | 0.802 | 0.643 | 5.57E-95 |
| WBP2 | 0 | 0.330843726 | 0.772 | 0.375 | 0 |
| ACAT2 | 9.06E-176 | 0.331554835 | 0.808 | 0.493 | 2.31E-171 |
| CDO1 | 7.93E-108 | 0.33156122 | 0.595 | 0.381 | 2.02E-103 |
| APIP | 0 | 0.331777825 | 0.682 | 0.231 | 0 |
| MAF1 | 3.29E-232 | 0.332009132 | 0.675 | 0.348 | 8.38E-228 |
| DAP | 1.24E-178 | 0.332074989 | 0.648 | 0.361 | 3.16E-174 |
| MDH2 | 5.07E-205 | 0.33210385 | 0.96 | 0.713 | 1.29E-200 |
| HIST1H4C | 3.03E-65 | 0.332665175 | 0.651 | 0.449 | 7.73E-61 |
| SRP72 | 0 | 0.332791642 | 0.766 | 0.357 | 0 |
| VCAM1 | 4.78E-53 | 0.333071424 | 0.265 | 0.148 | 1.22E-48 |
| UGT3A1 | 0 | 0.333615617 | 0.43 | 0.09 | 0 |
| SET | 4.76E-188 | 0.334159645 | 0.883 | 0.596 | 1.21E-183 |
| MRPL50 | 0 | 0.334681481 | 0.765 | 0.339 | 0 |
| PSMD11 | 1.65E-265 | 0.334942613 | 0.84 | 0.457 | 4.20E-261 |
| COX7B2 | 0 | 0.335018504 | 0.562 | 0.195 | 0 |
| CAPN1 | 2.05E-305 | 0.336547927 | 0.594 | 0.229 | 5.23E-301 |
| PRELID1 | 1.07E-67 | 0.33741329 | 0.781 | 0.689 | 2.73E-63 |
| INO80C | 0 | 0.339380319 | 0.573 | 0.199 | 0 |
| NUDT16 | 0 | 0.339481724 | 0.642 | 0.243 | 0 |
| TIMM8A | 6.88E-283 | 0.339700556 | 0.59 | 0.243 | 1.75E-278 |
| PMF1 | 2.03E-281 | 0.340477227 | 0.825 | 0.446 | 5.16E-277 |
| MRPS23 | 8.87E-303 | 0.340631275 | 0.741 | 0.341 | 2.26E-298 |
| SEC61B | 3.44E-171 | 0.341771091 | 0.979 | 0.878 | 8.76E-167 |
| ICT1 | 0 | 0.343383784 | 0.659 | 0.241 | 0 |
| ENSA | 1.01E-248 | 0.343867257 | 0.874 | 0.516 | 2.58E-244 |
| SYBU | 0 | 0.343970986 | 0.493 | 0.142 | 0 |
| PSMB4 | 5.80E-251 | 0.344242965 | 0.859 | 0.519 | 1.48E-246 |
| TMEM106C | 6.76E-217 | 0.344413367 | 0.794 | 0.451 | 1.72E-212 |
| NIT2 | 2.71E-300 | 0.348120467 | 0.817 | 0.433 | 6.89E-296 |
| TUFM | 1.11E-190 | 0.348687561 | 0.929 | 0.663 | 2.83E-186 |
| C17orf89 | 7.66E-85 | 0.348696348 | 0.694 | 0.554 | 1.95E-80 |
| CSDE1 | 1.55E-224 | 0.349487404 | 0.88 | 0.579 | 3.95E-220 |
| TOM1L1 | 0 | 0.349584273 | 0.705 | 0.25 | 0 |
| STIP1 | 0 | 0.350556609 | 0.764 | 0.338 | 0 |
| CDC20 | 1.68E-143 | 0.351109847 | 0.284 | 0.099 | 4.27E-139 |
| PSMD3 | 3.14E-194 | 0.352579505 | 0.696 | 0.399 | 8.00E-190 |
| TPM3 | 2.09E-186 | 0.352826003 | 0.886 | 0.608 | 5.32E-182 |
| COA3 | 2.36E-178 | 0.353235663 | 0.974 | 0.752 | 6.02E-174 |
| CAT | 3.69E-226 | 0.353893154 | 0.826 | 0.511 | 9.40E-222 |
| LDHA | 3.54E-55 | 0.354343952 | 0.988 | 0.906 | 9.03E-51 |
| PEPD | 1.74E-238 | 0.354529044 | 0.828 | 0.485 | 4.44E-234 |
| GAGE13 | 1.31E-290 | 0.354612183 | 0.354 | 0.082 | 3.34E-286 |
| POLR2G | 0 | 0.355219823 | 0.85 | 0.426 | 0 |
| RPL39 | 3.70E-141 | 0.355446869 | 1 | 0.992 | 9.42E-137 |
| HSPB11 | 1.36E-269 | 0.356452888 | 0.878 | 0.531 | 3.47E-265 |
| SCPEP1 | 2.09E-258 | 0.358757138 | 0.65 | 0.307 | 5.32E-254 |
| ATP5H | 7.78E-168 | 0.359276471 | 0.983 | 0.813 | 1.98E-163 |
| DAP3 | 0 | 0.360356705 | 0.778 | 0.386 | 0 |
| TAF9 | 8.97E-257 | 0.361306357 | 0.824 | 0.466 | 2.28E-252 |
| COPZ1 | 2.93E-234 | 0.361380797 | 0.844 | 0.528 | 7.47E-230 |
| BAD | 0 | 0.361580262 | 0.791 | 0.404 | 0 |
| G6PD | 4.99E-223 | 0.362387637 | 0.543 | 0.239 | 1.27E-218 |
| ADRM1 | 7.06E-238 | 0.363885093 | 0.911 | 0.592 | 1.80E-233 |
| FAM162A | 6.28E-162 | 0.363953174 | 0.938 | 0.7 | 1.60E-157 |
| CAP1 | 1.01E-222 | 0.364170044 | 0.834 | 0.491 | 2.56E-218 |
| DRAP1 | 1.89E-244 | 0.364391464 | 0.927 | 0.629 | 4.81E-240 |
| TMEM141 | 2.01E-236 | 0.365017871 | 0.889 | 0.556 | 5.12E-232 |
| SEPT4 | 0 | 0.365308116 | 0.316 | 0.034 | 0 |
| GPS1 | 1.17E-306 | 0.365681016 | 0.765 | 0.37 | 2.99E-302 |
| GAGE1 | 2.11E-173 | 0.366037859 | 0.505 | 0.236 | 5.37E-169 |
| S100A16 | 1.39E-117 | 0.367269479 | 0.881 | 0.689 | 3.55E-113 |
| COX7A2L | 2.45E-184 | 0.368580872 | 0.941 | 0.692 | 6.24E-180 |
| DCAF4L2 | 0 | 0.368764936 | 0.487 | 0.077 | 0 |
| ENY2 | 1.55E-219 | 0.368884853 | 0.986 | 0.774 | 3.94E-215 |
| EIF1 | 3.48E-149 | 0.369111828 | 1 | 0.993 | 8.87E-145 |
| NDUFB10 | 1.38E-264 | 0.36967057 | 0.958 | 0.711 | 3.53E-260 |
| ATP5L | 4.34E-265 | 0.370324445 | 0.998 | 0.941 | 1.11E-260 |
| HAAO | 0 | 0.370477186 | 0.715 | 0.317 | 0 |
| CXCL5 | 5.67E-150 | 0.370761132 | 0.154 | 0.024 | 1.45E-145 |
| BOP1 | 5.37E-246 | 0.372998849 | 0.438 | 0.161 | 1.37E-241 |
| PLEKHF1 | 0 | 0.373361246 | 0.411 | 0.087 | 0 |
| CCDC124 | 7.29E-187 | 0.373939363 | 0.779 | 0.47 | 1.86E-182 |
| EDF1 | 4.39E-278 | 0.373948672 | 0.993 | 0.892 | 1.12E-273 |
| MAGEA6 | 0 | 0.374278543 | 0.571 | 0.165 | 0 |
| AQP9 | 6.64E-108 | 0.374745718 | 0.505 | 0.318 | 1.69E-103 |
| PA2G4 | 1.10E-220 | 0.375211541 | 0.921 | 0.623 | 2.80E-216 |
| HSF1 | 0 | 0.375341916 | 0.671 | 0.305 | 0 |
| FBXW5 | 1.32E-252 | 0.375823249 | 0.713 | 0.386 | 3.36E-248 |
| MGST1 | 1.85E-179 | 0.378497269 | 1 | 0.92 | 4.72E-175 |
| ANGPTL3 | 1.50E-156 | 0.378604303 | 0.897 | 0.595 | 3.83E-152 |
| COPS5 | 0 | 0.378746722 | 0.791 | 0.37 | 0 |
| ETNK2 | 3.11E-223 | 0.379110359 | 0.568 | 0.273 | 7.92E-219 |
| HLF | 1.65E-262 | 0.379111527 | 0.387 | 0.121 | 4.20E-258 |
| S100P | 1.39E-84 | 0.380315572 | 0.247 | 0.11 | 3.55E-80 |
| UQCRH | 3.12E-235 | 0.38094263 | 0.996 | 0.905 | 7.95E-231 |
| TMEM261 | 0 | 0.381047912 | 0.677 | 0.251 | 0 |
| GPC3 | 2.85E-17 | 0.381427933 | 0.546 | 0.556 | 7.25E-13 |
| UQCRQ | 6.26E-120 | 0.382365019 | 0.999 | 0.955 | 1.59E-115 |
| CCT3 | 4.02E-248 | 0.382559627 | 0.947 | 0.634 | 1.02E-243 |
| MIEN1 | 5.10E-233 | 0.383350737 | 0.926 | 0.624 | 1.30E-228 |
| MRPS7 | 1.55E-254 | 0.383794229 | 0.895 | 0.55 | 3.95E-250 |
| C1orf43 | 1.93E-179 | 0.383879894 | 0.897 | 0.638 | 4.90E-175 |
| TUBA1C | 1.49E-281 | 0.384538617 | 0.717 | 0.322 | 3.80E-277 |
| RAC3 | 0 | 0.385491553 | 0.396 | 0.052 | 0 |
| ATP6V0E2 | 0 | 0.385812871 | 0.604 | 0.145 | 0 |
| UGT2B11 | 0 | 0.38599655 | 0.476 | 0.077 | 0 |
| CYP4F11 | 3.14E-292 | 0.386583931 | 0.507 | 0.181 | 8.01E-288 |
| ABRACL | 1.29E-292 | 0.386797047 | 0.668 | 0.305 | 3.28E-288 |
| HAX1 | 7.42E-282 | 0.387386288 | 0.876 | 0.524 | 1.89E-277 |
| NELFE | 0 | 0.388355922 | 0.844 | 0.403 | 0 |
| PAICS | 0 | 0.390083103 | 0.757 | 0.297 | 0 |
| COX6C | 1.65E-224 | 0.390478129 | 1 | 0.964 | 4.21E-220 |
| CNPY2 | 4.04E-271 | 0.390802091 | 0.928 | 0.656 | 1.03E-266 |
| SNF8 | 9.80E-177 | 0.391576226 | 0.764 | 0.501 | 2.50E-172 |
| TATDN1 | 2.55E-268 | 0.391772994 | 0.814 | 0.437 | 6.49E-264 |
| ACSM2B | 1.07E-168 | 0.393822674 | 0.72 | 0.442 | 2.73E-164 |
| GBP1 | 0 | 0.393862841 | 0.534 | 0.184 | 0 |
| BHMT2 | 1.45E-285 | 0.393993753 | 0.74 | 0.378 | 3.69E-281 |
| LINC01419 | 0 | 0.394060873 | 0.451 | 0.118 | 0 |
| RBP4 | 6.88E-56 | 0.394490106 | 0.968 | 0.917 | 1.75E-51 |
| EXOSC4 | 0 | 0.396480032 | 0.756 | 0.365 | 0 |
| GSTA4 | 0 | 0.396538911 | 0.611 | 0.216 | 0 |
| SWI5 | 0 | 0.39774041 | 0.745 | 0.284 | 0 |
| HDHD3 | 0 | 0.399160996 | 0.726 | 0.288 | 0 |
| RPL5 | 3.44E-190 | 0.399572952 | 0.999 | 0.958 | 8.76E-186 |
| GNG5 | 7.15E-165 | 0.400317772 | 0.953 | 0.758 | 1.82E-160 |
| ALG3 | 0 | 0.400465362 | 0.778 | 0.384 | 0 |
| MID1IP1 | 1.99E-191 | 0.400670327 | 0.613 | 0.311 | 5.08E-187 |
| MTTP | 0 | 0.400806627 | 0.693 | 0.295 | 0 |
| TOMM20 | 2.22E-296 | 0.401336141 | 0.908 | 0.559 | 5.67E-292 |
| PSMA1 | 2.78E-298 | 0.401792682 | 0.942 | 0.624 | 7.08E-294 |
| LRRC59 | 9.35E-308 | 0.402670291 | 0.829 | 0.439 | 2.38E-303 |
| RPL35 | 5.33E-281 | 0.405384398 | 1 | 0.982 | 1.36E-276 |
| SUMO2 | 6.33E-247 | 0.406964532 | 0.995 | 0.855 | 1.61E-242 |
| DKK1 | 4.73E-154 | 0.407167895 | 0.135 | 0.015 | 1.21E-149 |
| MYC | 2.00E-168 | 0.40827869 | 0.476 | 0.228 | 5.11E-164 |
| ZMAT2 | 4.56E-289 | 0.409836117 | 0.769 | 0.396 | 1.16E-284 |
| MSMO1 | 4.38E-217 | 0.411636351 | 0.805 | 0.486 | 1.11E-212 |
| CARHSP1 | 1.04E-217 | 0.413164563 | 0.899 | 0.635 | 2.65E-213 |
| HTATIP2 | 0 | 0.413611115 | 0.866 | 0.489 | 0 |
| SUB1 | 1.29E-165 | 0.415081418 | 0.993 | 0.921 | 3.28E-161 |
| HFE2 | 0 | 0.417257302 | 0.527 | 0.127 | 0 |
| TSKU | 0 | 0.417648573 | 0.456 | 0.132 | 0 |
| RPS23 | 1.39E-19 | 0.417769209 | 1 | 0.982 | 3.53E-15 |
| RAD23A | 0 | 0.418623691 | 0.87 | 0.465 | 0 |
| AOX1 | 0 | 0.419029264 | 0.586 | 0.205 | 0 |
| HMGCS1 | 1.10E-81 | 0.419065616 | 0.743 | 0.524 | 2.79E-77 |
| CAV2 | 1.78E-140 | 0.419206964 | 0.591 | 0.372 | 4.54E-136 |
| NASP | 0 | 0.41978318 | 0.69 | 0.301 | 0 |
| EIF3I | 7.24E-270 | 0.420777231 | 0.944 | 0.638 | 1.84E-265 |
| AIMP1 | 9.37E-295 | 0.422010569 | 0.805 | 0.427 | 2.39E-290 |
| HNRNPA1 | 7.11E-231 | 0.422946093 | 0.994 | 0.849 | 1.81E-226 |
| STMN1 | 1.29E-101 | 0.423872613 | 0.863 | 0.619 | 3.29E-97 |
| RARRES3 | 1.61E-289 | 0.426369652 | 0.775 | 0.387 | 4.10E-285 |
| OSGIN1 | 4.22E-148 | 0.426602607 | 0.647 | 0.401 | 1.08E-143 |
| MGST3 | 5.05E-218 | 0.427454483 | 0.929 | 0.719 | 1.29E-213 |
| TUBG1 | 0 | 0.427847557 | 0.672 | 0.211 | 0 |
| MRPL24 | 0 | 0.428203465 | 0.893 | 0.523 | 0 |
| BLVRB | 3.03E-233 | 0.429107144 | 0.963 | 0.737 | 7.71E-229 |
| SOD1 | 3.07E-217 | 0.429826754 | 1 | 0.941 | 7.82E-213 |
| KPNA2 | 5.58E-275 | 0.430534942 | 0.669 | 0.289 | 1.42E-270 |
| SQLE | 9.26E-250 | 0.431012082 | 0.785 | 0.437 | 2.36E-245 |
| CTSA | 1.41E-221 | 0.431568327 | 0.932 | 0.648 | 3.58E-217 |
| BLOC1S1 | 1.69E-283 | 0.433474016 | 0.943 | 0.692 | 4.30E-279 |
| TCEB1 | 9.98E-249 | 0.433831614 | 0.951 | 0.69 | 2.54E-244 |
| TDO2 | 9.97E-15 | 0.433941635 | 0.628 | 0.59 | 2.54E-10 |
| SLC9A3R1 | 4.93E-159 | 0.436894946 | 0.854 | 0.599 | 1.25E-154 |
| MRPL36 | 0 | 0.43794407 | 0.892 | 0.486 | 0 |
| IL13RA2 | 1.49E-161 | 0.438125513 | 0.166 | 0.027 | 3.80E-157 |
| DHCR7 | 7.04E-276 | 0.438405232 | 0.684 | 0.329 | 1.79E-271 |
| UBE2L6 | 0 | 0.440426345 | 0.725 | 0.307 | 0 |
| COA4 | 0 | 0.440788926 | 0.874 | 0.46 | 0 |
| CYP7A1 | 0 | 0.440797056 | 0.301 | 0.046 | 0 |
| RNH1 | 0 | 0.441588496 | 0.88 | 0.473 | 0 |
| INSIG1 | 1.03E-33 | 0.442415041 | 0.724 | 0.641 | 2.63E-29 |
| TRMT112 | 0 | 0.442686615 | 0.987 | 0.762 | 0 |
| PAGE2B | 1.46E-108 | 0.443167794 | 0.207 | 0.068 | 3.71E-104 |
| TECR | 1.00E-296 | 0.443211807 | 0.899 | 0.542 | 2.56E-292 |
| RPL23A | 3.04E-137 | 0.443955354 | 1 | 0.988 | 7.76E-133 |
| ARF1 | 2.92E-201 | 0.444014925 | 0.922 | 0.685 | 7.43E-197 |
| RBCK1 | 9.12E-165 | 0.444680829 | 0.79 | 0.517 | 2.32E-160 |
| RHOC | 0 | 0.444777269 | 0.945 | 0.63 | 0 |
| TUBB4B | 1.06E-281 | 0.44578448 | 0.824 | 0.441 | 2.70E-277 |
| ENO1 | 1.90E-173 | 0.446001867 | 0.998 | 0.894 | 4.85E-169 |
| GLRX | 0 | 0.447409985 | 0.779 | 0.399 | 0 |
| TMEM14A | 9.88E-279 | 0.447435918 | 0.964 | 0.692 | 2.52E-274 |
| CKS1B | 2.31E-200 | 0.44870076 | 0.857 | 0.534 | 5.87E-196 |
| MALAT1 | 5.26E-283 | 0.44931282 | 1 | 0.961 | 1.34E-278 |
| PSMC5 | 0 | 0.45081259 | 0.915 | 0.547 | 0 |
| PSMB3 | 1.06E-241 | 0.451085554 | 0.993 | 0.844 | 2.70E-237 |
| NAPRT | 8.09E-222 | 0.451290397 | 0.716 | 0.441 | 2.06E-217 |
| PSMA7 | 3.88E-263 | 0.451892356 | 0.994 | 0.887 | 9.90E-259 |
| OLA1 | 0 | 0.453002026 | 0.8 | 0.371 | 0 |
| HMGN2 | 2.70E-149 | 0.453272417 | 0.977 | 0.79 | 6.88E-145 |
| NHP2 | 1.44E-184 | 0.456209638 | 0.931 | 0.656 | 3.68E-180 |
| RPS14 | 1.62E-31 | 0.456961918 | 1 | 0.995 | 4.12E-27 |
| FDPS | 3.81E-170 | 0.456987194 | 0.915 | 0.684 | 9.71E-166 |
| RAB5C | 6.77E-306 | 0.457359561 | 0.958 | 0.634 | 1.72E-301 |
| RPL37 | 3.09E-301 | 0.459160911 | 1 | 0.984 | 7.87E-297 |
| CMB9-22P13.1 | 0 | 0.461467948 | 0.512 | 0.063 | 0 |
| NDUFS5 | 4.32E-292 | 0.46336186 | 0.989 | 0.849 | 1.10E-287 |
| SORD | 1.76E-221 | 0.464713316 | 0.818 | 0.555 | 4.47E-217 |
| HIST1H1C | 0 | 0.467680131 | 0.749 | 0.333 | 0 |
| GCLM | 2.30E-301 | 0.46810818 | 0.721 | 0.346 | 5.86E-297 |
| UQCRB | 9.17E-276 | 0.468442764 | 1 | 0.957 | 2.34E-271 |
| UGT2B15 | 3.19E-73 | 0.468632395 | 0.736 | 0.513 | 8.14E-69 |
| LINC01287 | 0 | 0.469410827 | 0.437 | 0.055 | 0 |
| GSTK1 | 2.73E-225 | 0.471062594 | 0.967 | 0.789 | 6.95E-221 |
| CYP2C9 | 2.59E-295 | 0.472053279 | 0.528 | 0.17 | 6.60E-291 |
| C1S | 1.07E-261 | 0.472372633 | 0.92 | 0.637 | 2.74E-257 |
| CFL1 | 1.25E-277 | 0.472450013 | 0.997 | 0.911 | 3.19E-273 |
| MTCH2 | 0 | 0.473282206 | 0.929 | 0.599 | 0 |
| MRPL12 | 1.90E-205 | 0.475320454 | 0.873 | 0.606 | 4.84E-201 |
| TCEA3 | 0 | 0.480159263 | 0.828 | 0.442 | 0 |
| MT-ND3 | 2.91E-198 | 0.482795179 | 0.996 | 0.874 | 7.43E-194 |
| GPX1 | 1.31E-152 | 0.484840653 | 0.959 | 0.842 | 3.33E-148 |
| SELENBP1 | 0 | 0.484954102 | 0.776 | 0.37 | 0 |
| HLA-DRB5 | 4.62E-300 | 0.485787171 | 0.448 | 0.147 | 1.18E-295 |
| ATP5G1 | 3.68E-212 | 0.487059645 | 0.991 | 0.833 | 9.38E-208 |
| FMO3 | 0 | 0.488634967 | 0.734 | 0.276 | 0 |
| PSMB9 | 0 | 0.488852313 | 0.831 | 0.43 | 0 |
| ADIRF | 0 | 0.489019443 | 0.434 | 0.117 | 0 |
| ATP1B1 | 6.30E-136 | 0.489365614 | 0.825 | 0.586 | 1.60E-131 |
| TXNL1 | 0 | 0.492791636 | 0.883 | 0.507 | 0 |
| DEGS1 | 6.41E-299 | 0.4931349 | 0.577 | 0.233 | 1.63E-294 |
| RPL23 | 2.17E-158 | 0.493334156 | 0.998 | 0.946 | 5.52E-154 |
| MRPL55 | 0 | 0.493857064 | 0.941 | 0.643 | 0 |
| PPIA | 4.49E-249 | 0.497894089 | 1 | 0.942 | 1.14E-244 |
| LGALS4 | 1.77E-203 | 0.498147269 | 0.975 | 0.739 | 4.52E-199 |
| CXCL10 | 5.27E-22 | 0.498433721 | 0.275 | 0.191 | 1.34E-17 |
| GSDMD | 0 | 0.499011009 | 0.878 | 0.508 | 0 |
| CCL26 | 5.33E-55 | 0.499116586 | 0.1 | 0.032 | 1.36E-50 |
| SNHG8 | 1.66E-215 | 0.499801195 | 0.809 | 0.518 | 4.22E-211 |
| TFF1 | 4.43E-43 | 0.500344375 | 0.11 | 0.044 | 1.13E-38 |
| YIF1A | 0 | 0.500844889 | 0.916 | 0.542 | 0 |
| GRINA | 9.32E-254 | 0.501516623 | 0.85 | 0.616 | 2.38E-249 |
| HLA-DRB1 | 0 | 0.501542254 | 0.812 | 0.431 | 0 |
| PMVK | 0 | 0.50195152 | 0.868 | 0.474 | 0 |
| RPL14 | 2.20E-178 | 0.50220957 | 0.998 | 0.957 | 5.60E-174 |
| NDUFS8 | 0 | 0.502363987 | 0.938 | 0.609 | 0 |
| NDUFB7 | 7.43E-268 | 0.502746836 | 0.98 | 0.777 | 1.89E-263 |
| PGA5 | 1.58E-37 | 0.504079474 | 0.028 | 0.002 | 4.03E-33 |
| DCXR | 5.63E-105 | 0.504479587 | 0.923 | 0.788 | 1.43E-100 |
| HLA-DRA | 3.96E-158 | 0.505391477 | 0.926 | 0.768 | 1.01E-153 |
| HSPA8 | 4.37E-201 | 0.507620503 | 0.987 | 0.832 | 1.11E-196 |
| ADH5 | 0 | 0.508190653 | 0.916 | 0.567 | 0 |
| MTDH | 1.01E-159 | 0.510720531 | 0.903 | 0.679 | 2.56E-155 |
| RPL7A | 0 | 0.511274894 | 0.999 | 0.932 | 0 |
| PSMB7 | 0 | 0.513088139 | 0.947 | 0.624 | 0 |
| H2AFZ | 6.27E-189 | 0.513541254 | 0.963 | 0.727 | 1.60E-184 |
| EIF3E | 7.61E-126 | 0.515464843 | 0.983 | 0.813 | 1.94E-121 |
| RPL38 | 7.67E-89 | 0.518578533 | 1 | 0.973 | 1.95E-84 |
| HN1 | 0 | 0.521077057 | 0.839 | 0.435 | 0 |
| COX8A | 0 | 0.521999301 | 0.984 | 0.832 | 0 |
| PUF60 | 0 | 0.527671408 | 0.948 | 0.582 | 0 |
| HPD | 8.52E-229 | 0.530668174 | 0.469 | 0.169 | 2.17E-224 |
| BAAT | 5.63E-170 | 0.533969355 | 0.855 | 0.578 | 1.43E-165 |
| ALDH3A2 | 0 | 0.535904899 | 0.752 | 0.347 | 0 |
| PSMD4 | 0 | 0.53763226 | 0.972 | 0.691 | 0 |
| FGG | 5.62E-135 | 0.538375987 | 0.991 | 0.874 | 1.43E-130 |
| CCT5 | 0 | 0.543655448 | 0.901 | 0.515 | 0 |
| SDC2 | 3.97E-201 | 0.545951834 | 0.86 | 0.677 | 1.01E-196 |
| TMEM45B | 0 | 0.551027585 | 0.6 | 0.113 | 0 |
| UBA52 | 8.32E-101 | 0.552495789 | 0.999 | 0.975 | 2.12E-96 |
| FGB | 2.62E-203 | 0.559875228 | 0.989 | 0.799 | 6.67E-199 |
| SERBP1 | 0 | 0.565909906 | 0.965 | 0.725 | 0 |
| FUNDC2 | 7.75E-276 | 0.570358445 | 0.773 | 0.416 | 1.98E-271 |
| GAGE2A | 8.30E-107 | 0.570845391 | 0.365 | 0.19 | 2.11E-102 |
| CYSTM1 | 0 | 0.577557787 | 0.943 | 0.687 | 0 |
| PGD | 0 | 0.577843972 | 0.704 | 0.283 | 0 |
| UGT2B4 | 5.82E-181 | 0.578552762 | 0.872 | 0.598 | 1.48E-176 |
| PRDX6 | 0 | 0.581208739 | 0.994 | 0.877 | 0 |
| PHPT1 | 0 | 0.58704056 | 0.978 | 0.675 | 0 |
| TFF3 | 2.40E-53 | 0.587498422 | 0.247 | 0.141 | 6.13E-49 |
| PSMB2 | 0 | 0.588420031 | 0.967 | 0.673 | 0 |
| C19orf53 | 1.27E-212 | 0.591552597 | 0.977 | 0.755 | 3.24E-208 |
| CMBL | 0 | 0.592377852 | 0.859 | 0.433 | 0 |
| ANAPC11 | 0 | 0.595972617 | 0.993 | 0.842 | 0 |
| POP4 | 0 | 0.596601337 | 0.67 | 0.24 | 0 |
| PEG10 | 3.75E-189 | 0.599212874 | 0.434 | 0.2 | 9.54E-185 |
| C1R | 0 | 0.599634352 | 0.863 | 0.454 | 0 |
| C8orf59 | 0 | 0.602324832 | 0.956 | 0.541 | 0 |
| NDUFB9 | 0 | 0.605557703 | 0.988 | 0.845 | 0 |
| SPON2 | 5.70E-121 | 0.606875075 | 0.679 | 0.475 | 1.45E-116 |
| FADS1 | 0 | 0.610714332 | 0.526 | 0.115 | 0 |
| TALDO1 | 0 | 0.620019916 | 0.952 | 0.648 | 0 |
| VPS28 | 0 | 0.622489771 | 0.972 | 0.688 | 0 |
| PTGR1 | 4.29E-205 | 0.627893379 | 0.911 | 0.633 | 1.09E-200 |
| APOA5 | 0 | 0.632327909 | 0.702 | 0.31 | 0 |
| PAGE1 | 2.17E-194 | 0.635923276 | 0.29 | 0.08 | 5.53E-190 |
| CYC1 | 1.77E-243 | 0.636384008 | 0.82 | 0.714 | 4.52E-239 |
| NDUFS6 | 0 | 0.638546759 | 0.992 | 0.818 | 0 |
| CYP3A5 | 0 | 0.640509645 | 0.862 | 0.538 | 0 |
| HP | 0 | 0.644417632 | 0.843 | 0.318 | 0 |
| TSPAN8 | 0 | 0.645647733 | 0.815 | 0.338 | 0 |
| GAPDH | 0 | 0.648562844 | 1 | 0.987 | 0 |
| EEF1D | 1.87E-253 | 0.651339513 | 0.999 | 0.938 | 4.77E-249 |
| TXN | 1.32E-239 | 0.653396559 | 0.999 | 0.943 | 3.35E-235 |
| TUBB | 2.46E-290 | 0.655044344 | 0.982 | 0.754 | 6.27E-286 |
| HLA-DMA | 0 | 0.658752157 | 0.67 | 0.249 | 0 |
| RPL30 | 0 | 0.66175739 | 1 | 0.986 | 0 |
| TM7SF2 | 7.70E-129 | 0.663428736 | 0.579 | 0.383 | 1.96E-124 |
| RPL27 | 1.86E-229 | 0.663800874 | 1 | 0.973 | 4.74E-225 |
| CBR1 | 9.63E-138 | 0.669845551 | 0.909 | 0.69 | 2.45E-133 |
| RPL19 | 1.24E-171 | 0.674945546 | 1 | 0.993 | 3.15E-167 |
| PRDX1 | 0 | 0.68211579 | 1 | 0.939 | 0 |
| ITM2A | 1.44E-173 | 0.687892665 | 0.3 | 0.1 | 3.66E-169 |
| STRA13 | 7.80E-246 | 0.689058421 | 0.918 | 0.614 | 1.99E-241 |
| CSTA | 0 | 0.695540356 | 0.731 | 0.174 | 0 |
| GRHPR | 0 | 0.695692711 | 0.924 | 0.603 | 0 |
| GC | 1.04E-125 | 0.698131082 | 0.987 | 0.78 | 2.65E-121 |
| CTSE | 1.12E-130 | 0.704173871 | 0.166 | 0.038 | 2.86E-126 |
| SLC25A33 | 0 | 0.706628076 | 0.699 | 0.307 | 0 |
| RPL7 | 2.40E-240 | 0.707191237 | 1 | 0.993 | 6.11E-236 |
| LEAP2 | 4.60E-98 | 0.71005803 | 0.786 | 0.614 | 1.17E-93 |
| PHB | 7.13E-250 | 0.712965615 | 0.958 | 0.671 | 1.82E-245 |
| EIF3H | 0 | 0.713381071 | 0.966 | 0.662 | 0 |
| TM4SF1 | 1.86E-265 | 0.717205782 | 0.728 | 0.398 | 4.74E-261 |
| RPL8 | 0 | 0.723646793 | 1 | 0.993 | 0 |
| NPM1 | 2.73E-128 | 0.727277225 | 0.996 | 0.901 | 6.96E-124 |
| LGALS3BP | 0 | 0.737500643 | 0.652 | 0.268 | 0 |
| TUBA1B | 1.11E-250 | 0.755094292 | 0.965 | 0.721 | 2.83E-246 |
| IGLC2 | 5.91E-41 | 0.770348318 | 0.456 | 0.382 | 1.51E-36 |
| MPC2 | 4.50E-274 | 0.772147445 | 0.994 | 0.904 | 1.15E-269 |
| ADH1A | 0 | 0.77421004 | 0.681 | 0.314 | 0 |
| PPP1R16A | 0 | 0.779013072 | 0.904 | 0.641 | 0 |
| HPR | 0 | 0.786991768 | 0.567 | 0.152 | 0 |
| GSTA2 | 8.03E-258 | 0.789935049 | 0.706 | 0.406 | 2.05E-253 |
| AKR1C3 | 0 | 0.812946619 | 0.987 | 0.686 | 0 |
| TKT | 1.92E-156 | 0.81883917 | 0.893 | 0.647 | 4.90E-152 |
| GAGE12H | 1.65E-117 | 0.822772507 | 0.185 | 0.053 | 4.21E-113 |
| SPP1 | 4.19E-181 | 0.830507761 | 0.663 | 0.378 | 1.07E-176 |
| CFH | 1.00E-194 | 0.83977319 | 0.879 | 0.652 | 2.56E-190 |
| TFF2 | 2.82E-120 | 0.841578438 | 0.423 | 0.21 | 7.20E-116 |
| S100A9 | 1.53E-41 | 0.843231183 | 0.259 | 0.147 | 3.89E-37 |
| CES1 | 3.58E-109 | 0.877627804 | 0.984 | 0.861 | 9.11E-105 |
| NME1 | 0 | 0.88975673 | 0.942 | 0.636 | 0 |
| IFI27 | 0 | 0.928566055 | 0.705 | 0.324 | 0 |
| ADH1C | 1.71E-225 | 0.934693225 | 0.59 | 0.312 | 4.37E-221 |
| CD74 | 1.82E-99 | 0.970218883 | 0.946 | 0.86 | 4.63E-95 |
| ALDH1L1 | 2.98E-290 | 1.018233075 | 0.611 | 0.315 | 7.59E-286 |
| AGR2 | 1.47E-143 | 1.067505108 | 0.256 | 0.085 | 3.74E-139 |
| ADH4 | 1.76E-230 | 1.06918327 | 0.435 | 0.195 | 4.48E-226 |
| ADH1B | 4.57E-277 | 1.07182805 | 0.744 | 0.473 | 1.16E-272 |
| AKR1C4 | 0 | 1.079102841 | 0.859 | 0.425 | 0 |
| ALDH3A1 | 0 | 1.122447352 | 0.334 | 0.011 | 0 |
| AKR1C2 | 2.65E-178 | 1.136342272 | 0.926 | 0.635 | 6.75E-174 |
| IGHG4 | 3.97E-69 | 1.156896793 | 0.434 | 0.313 | 1.01E-64 |
| GPX2 | 0 | 1.227239773 | 0.79 | 0.46 | 0 |
| BHMT | 0 | 1.231426248 | 0.662 | 0.252 | 0 |
| SNHG25 | 8.13E-125 | 1.249265561 | 0.64 | 0.482 | 2.07E-120 |
| CYP2E1 | 4.61E-90 | 1.265779965 | 0.472 | 0.363 | 1.17E-85 |
| ALDH1A1 | 0 | 1.293607712 | 0.993 | 0.649 | 0 |
| AKR1C1 | 0 | 1.319496251 | 0.975 | 0.688 | 0 |
| CHI3L1 | 0 | 1.490472094 | 0.338 | 0.028 | 0 |
| HULC | 0 | 1.502356122 | 0.808 | 0.509 | 0 |
| SQSTM1 | 3.32E-174 | 1.576089664 | 0.925 | 0.794 | 8.45E-170 |
| LYZ | 1.03E-115 | 1.700054969 | 0.626 | 0.401 | 2.63E-111 |
| GGH | 0 | 1.850901107 | 0.948 | 0.691 | 0 |
| GLUL | 0 | 2.028371476 | 0.934 | 0.694 | 0 |

Tab S3. The 289 prognostic TRGs by univariate Cox analysis.

| id | HR | HR.95L | HR.95H | pvalue |
| --- | --- | --- | --- | --- |
| SQSTM1 | 2.961492275 | 1.609236548 | 4.553066291 | 0.00171264 |
| HULC | 0.287953387 | 0.094942031 | 0.780233447 | 0.011985358 |
| BHMT | 0.67570076 | 0.486569685 | 0.918132045 | 0.011237083 |
| ADH1B | 0.615496187 | 0.455535042 | 0.817633572 | 0.000588462 |
| ADH4 | 0.611522344 | 0.480909538 | 0.76920048 | 1.41E-05 |
| AGR2 | 1.406108691 | 1.162480944 | 1.677591499 | 0.000626354 |
| ADH1C | 0.658044227 | 0.490194526 | 0.868274947 | 0.002574671 |
| NME1 | 2.173194771 | 1.220608061 | 3.359129849 | 0.011187762 |
| S100A9 | 2.029378238 | 1.523364322 | 2.601221076 | 9.13E-06 |
| TFF2 | 1.462877941 | 1.187059117 | 1.772512257 | 0.00053626 |
| CFH | 0.526986246 | 0.281086579 | 0.92884942 | 0.025331234 |
| SPP1 | 1.635837488 | 1.328421523 | 1.977990771 | 1.12E-05 |
| TKT | 2.941539482 | 1.851452823 | 4.179309695 | 7.56E-05 |
| AKR1C3 | 3.10220679 | 1.576856274 | 4.908218455 | 0.003075033 |
| HPR | 0.677007892 | 0.49058504 | 0.914844227 | 0.010163312 |
| PPP1R16A | 2.116065398 | 1.120409474 | 3.38869334 | 0.02374322 |
| ADH1A | 0.629639839 | 0.422973409 | 0.909829578 | 0.012620752 |
| TUBA1B | 2.230732783 | 1.270959332 | 3.413358488 | 0.007686436 |
| LGALS3BP | 1.589744442 | 1.025461924 | 2.293082281 | 0.039075856 |
| NPM1 | 4.447429131 | 2.555749375 | 6.488834711 | 4.11E-05 |
| RPL8 | 3.216807963 | 1.47755537 | 5.306466021 | 0.006958247 |
| EIF3H | 3.23068115 | 1.522467964 | 5.269042678 | 0.005432188 |
| PHB | 3.969836165 | 1.870043381 | 6.358132703 | 0.002070666 |
| CTSE | 1.461121183 | 1.098340553 | 1.885085314 | 0.010399449 |
| GRHPR | 0.275070619 | 0.090135115 | 0.752224384 | 0.009618797 |
| PRDX1 | 5.949126675 | 3.335781494 | 8.669407184 | 2.25E-05 |
| RPL19 | 3.634696911 | 1.176415658 | 6.707487527 | 0.030344176 |
| RPL27 | 3.719228225 | 1.436747803 | 6.455596743 | 0.012645388 |
| TUBB | 3.315500849 | 1.536631382 | 5.432481072 | 0.005563463 |
| TXN | 3.839156073 | 1.700902435 | 6.316933994 | 0.004306907 |
| GAPDH | 5.776995896 | 3.081608554 | 8.604343213 | 7.67E-05 |
| CYP3A5 | 0.568416015 | 0.342141234 | 0.904027531 | 0.01562478 |
| NDUFS6 | 2.880416395 | 1.277162747 | 4.879981895 | 0.015423662 |
| PAGE1 | 1.661096761 | 1.377982844 | 1.972575351 | 5.51E-07 |
| APOA5 | 0.726495671 | 0.526076759 | 0.981044302 | 0.036574918 |
| TALDO1 | 4.696992854 | 2.656098102 | 6.885913572 | 4.66E-05 |
| C1R | 0.385111659 | 0.142142279 | 0.929103492 | 0.032165309 |
| POP4 | 2.622274394 | 1.054708286 | 4.683042158 | 0.04005754 |
| C19orf53 | 3.191972212 | 1.112601657 | 5.857609125 | 0.034731413 |
| PSMB2 | 6.251903676 | 3.632678031 | 8.957193459 | 7.16E-06 |
| PRDX6 | 4.992572214 | 1.960978338 | 8.364744304 | 0.004512463 |
| PGD | 4.290276156 | 2.718763852 | 5.971298322 | 1.89E-06 |
| GAGE2A | 1.935926298 | 1.544238059 | 2.369554303 | 1.59E-07 |
| FUNDC2 | 4.073813835 | 2.668730821 | 5.57959352 | 5.86E-07 |
| SERBP1 | 6.377806701 | 3.457107682 | 9.403466334 | 4.14E-05 |
| CCT5 | 4.481647296 | 2.762511034 | 6.316869289 | 4.31E-06 |
| PSMD4 | 3.210240014 | 1.406376258 | 5.401999714 | 0.010162344 |
| BAAT | 0.617970917 | 0.395266868 | 0.931416645 | 0.020269996 |
| HPD | 0.704652259 | 0.52056861 | 0.935611361 | 0.014588541 |
| PUF60 | 3.247364237 | 1.398020205 | 5.496345375 | 0.010926102 |
| EIF3E | 2.358430262 | 1.081116732 | 4.024543932 | 0.033675598 |
| RPL7A | 3.525175831 | 1.263881584 | 6.31946948 | 0.022125572 |
| MTDH | 2.543151089 | 1.048480422 | 4.512466189 | 0.040769179 |
| HSPA8 | 4.38738475 | 2.126278326 | 6.894681233 | 0.000975378 |
| DCXR | 0.462870841 | 0.202830529 | 0.96195652 | 0.038078629 |
| RPL14 | 2.765277745 | 1.165522609 | 4.809882949 | 0.02528898 |
| YIF1A | 3.958700593 | 1.436388773 | 6.966197854 | 0.014337209 |
| TFF1 | 1.276281419 | 1.027557003 | 1.559427937 | 0.028095868 |
| CCL26 | 1.501185065 | 1.086856253 | 1.993352855 | 0.015095467 |
| PPIA | 6.801847073 | 4.059543371 | 9.609247446 | 2.35E-06 |
| RPL23 | 3.021769908 | 1.179322025 | 5.361151471 | 0.025995463 |
| DEGS1 | 3.344044684 | 1.860291337 | 5.038042518 | 0.000500746 |
| TXNL1 | 4.191473435 | 1.889316105 | 6.79814705 | 0.002597523 |
| FMO3 | 0.63578885 | 0.451251219 | 0.875880395 | 0.004808864 |
| CFL1 | 7.634153737 | 4.606259499 | 10.70883091 | 1.22E-06 |
| C1S | 0.258624674 | 0.094668583 | 0.649287504 | 0.002685408 |
| CYP2C9 | 0.619166665 | 0.461789861 | 0.816820494 | 0.000500733 |
| UGT2B15 | 0.708455085 | 0.551619313 | 0.897791793 | 0.003798758 |
| GCLM | 2.581599872 | 1.53563653 | 3.816624742 | 0.001104729 |
| SORD | 0.521527963 | 0.31466748 | 0.83032273 | 0.004995676 |
| NDUFS5 | 4.722959529 | 2.198390906 | 7.498686444 | 0.001195584 |
| RPL37 | 3.166701025 | 1.472382575 | 5.204749719 | 0.006769999 |
| RAB5C | 4.836243066 | 2.364082505 | 7.525019922 | 0.000598547 |
| RPS14 | 3.325193965 | 1.17250965 | 6.042583626 | 0.028904116 |
| NHP2 | 3.165602268 | 1.500210773 | 5.160261891 | 0.005717576 |
| HMGN2 | 2.826706895 | 1.383415299 | 4.590171966 | 0.007820216 |
| OLA1 | 5.160057974 | 3.251379224 | 7.157089891 | 7.50E-07 |
| PSMA7 | 5.339115169 | 2.781783714 | 8.056399276 | 0.00015534 |
| PSMC5 | 3.720051042 | 1.461032708 | 6.419476018 | 0.011484267 |
| CKS1B | 2.85727665 | 1.625797306 | 4.300394323 | 0.001060133 |
| ENO1 | 6.274374192 | 4.161275631 | 8.437094939 | 1.79E-08 |
| RHOC | 3.837785681 | 2.058638424 | 5.829339313 | 0.000409647 |
| RBCK1 | 2.965171426 | 1.419525743 | 4.842022179 | 0.007344743 |
| TRMT112 | 5.112984229 | 2.22636883 | 8.266528193 | 0.001843538 |
| RNH1 | 4.104257215 | 1.828818718 | 6.697751644 | 0.003138349 |
| CYP7A1 | 0.727991384 | 0.587574541 | 0.892883261 | 0.0019661 |
| MRPL36 | 4.577407824 | 2.31868387 | 7.046368312 | 0.000443913 |
| SLC9A3R1 | 4.449385376 | 2.143820526 | 7.001183634 | 0.000991619 |
| CTSA | 4.317149371 | 2.327095675 | 6.495416763 | 0.000200508 |
| KPNA2 | 3.962511162 | 2.766781099 | 5.235044938 | 1.19E-08 |
| TUBG1 | 4.650726366 | 3.009148864 | 6.383811189 | 3.92E-07 |
| STMN1 | 2.926018386 | 2.0049974 | 3.949868159 | 2.38E-06 |
| HNRNPA1 | 2.720667694 | 1.155427842 | 4.726440059 | 0.026086528 |
| EIF3I | 5.567764672 | 2.941611032 | 8.338082509 | 0.000104214 |
| NASP | 3.691357043 | 2.286410166 | 5.23363874 | 1.52E-05 |
| AOX1 | 0.70413648 | 0.505374408 | 0.958769503 | 0.025065241 |
| SUB1 | 4.567473995 | 2.772712794 | 6.482378041 | 6.69E-06 |
| HTATIP2 | 2.950091548 | 1.521694409 | 4.654762284 | 0.003503416 |
| SUMO2 | 4.611545575 | 2.250029187 | 7.20285338 | 0.000733601 |
| LRRC59 | 3.025766705 | 1.476746044 | 4.888604127 | 0.005489809 |
| PSMA1 | 2.902121454 | 1.476847814 | 4.615335652 | 0.004521186 |
| MID1IP1 | 2.496153505 | 1.475020754 | 3.712096769 | 0.001696265 |
| ALG3 | 5.619588684 | 3.042113429 | 8.328993559 | 6.16E-05 |
| RPL5 | 5.106811137 | 2.431717108 | 7.998323796 | 0.000699971 |
| EXOSC4 | 2.236813743 | 1.279774781 | 3.413743421 | 0.007131748 |
| RBP4 | 0.429533845 | 0.230672156 | 0.760150535 | 0.002752973 |
| TATDN1 | 2.30601036 | 1.161057156 | 3.764489838 | 0.020442848 |
| SNF8 | 2.765442781 | 1.239934905 | 4.683554938 | 0.017417752 |
| CNPY2 | 2.556908311 | 1.22708838 | 4.232038948 | 0.01618255 |
| PAICS | 3.093266394 | 1.285653845 | 5.339033715 | 0.016859178 |
| HAX1 | 4.510349635 | 2.112321741 | 7.168433677 | 0.001318343 |
| RAC3 | 1.785775471 | 1.115268328 | 2.617802114 | 0.017944425 |
| TUBA1C | 2.964873128 | 1.836804729 | 4.249218057 | 0.000112242 |
| C1orf43 | 5.878968047 | 2.671942332 | 9.285107572 | 0.000817991 |
| MRPS7 | 4.910710144 | 2.332953072 | 7.716969401 | 0.000820638 |
| CCT3 | 5.778253922 | 3.323827143 | 8.335885304 | 1.24E-05 |
| UQCRH | 6.471801136 | 3.985032444 | 9.022765811 | 8.07E-07 |
| S100P | 1.277866295 | 1.045570675 | 1.539839384 | 0.017418102 |
| HLF | 0.67643442 | 0.45183314 | 0.980347568 | 0.038405772 |
| COPS5 | 3.136830676 | 1.282840673 | 5.439839869 | 0.017480887 |
| FBXW5 | 3.482203288 | 1.346802027 | 6.089080148 | 0.015906745 |
| HSF1 | 3.24353212 | 1.755168026 | 4.963760775 | 0.000988196 |
| PA2G4 | 6.069765165 | 3.603876322 | 8.619870663 | 3.91E-06 |
| AQP9 | 0.654734867 | 0.481444709 | 0.873939623 | 0.003430127 |
| MAGEA6 | 1.432542517 | 1.196966453 | 1.693231507 | 0.000145389 |
| CCDC124 | 4.066536298 | 2.13945137 | 6.207116683 | 0.000418401 |
| BOP1 | 2.79503783 | 1.673575679 | 4.09707018 | 0.000465784 |
| CXCL5 | 1.520007306 | 1.221283525 | 1.856141623 | 0.000296963 |
| EIF1 | 3.726269603 | 1.042924848 | 7.151422824 | 0.044753483 |
| ENY2 | 2.789832145 | 1.183984916 | 4.833371595 | 0.02340211 |
| COX7A2L | 4.546478588 | 2.010721108 | 7.376232939 | 0.002313281 |
| S100A16 | 3.068866448 | 1.776254187 | 4.556066197 | 0.000434281 |
| GAGE1 | 2.031633125 | 1.280848769 | 2.939623387 | 0.004106803 |
| GPS1 | 3.892611389 | 1.653353227 | 6.498735675 | 0.005740098 |
| TMEM141 | 2.562354481 | 1.367360064 | 4.021304896 | 0.005939493 |
| DRAP1 | 6.184629132 | 3.080245755 | 9.432695197 | 0.000215991 |
| CAP1 | 6.407928262 | 3.617102004 | 9.289524213 | 1.53E-05 |
| ADRM1 | 3.809919781 | 1.314199188 | 6.846932869 | 0.020446707 |
| G6PD | 2.74080872 | 2.037586251 | 3.511570043 | 1.76E-08 |
| BAD | 2.340393413 | 1.046008718 | 4.045613661 | 0.04018431 |
| COPZ1 | 4.980944161 | 2.228070945 | 7.993024637 | 0.001534704 |
| TAF9 | 4.389420697 | 2.509231132 | 6.424110492 | 5.05E-05 |
| DAP3 | 4.408258294 | 2.130698809 | 6.932460159 | 0.000989477 |
| SCPEP1 | 1.878132779 | 1.231566316 | 2.657959523 | 0.004889968 |
| POLR2G | 6.649183214 | 3.830854132 | 9.54567556 | 7.81E-06 |
| LDHA | 6.675810392 | 4.119054096 | 9.292020357 | 6.76E-07 |
| TPM3 | 4.312285534 | 2.455171279 | 6.328894491 | 6.16E-05 |
| PSMD3 | 2.89557043 | 1.10244559 | 5.215990975 | 0.03435009 |
| CDC20 | 2.438734948 | 1.81689047 | 3.129035322 | 2.06E-07 |
| STIP1 | 6.299925983 | 3.955057146 | 8.707549854 | 3.74E-07 |
| CSDE1 | 3.743850901 | 1.616801822 | 6.235640069 | 0.005880725 |
| TMEM106C | 4.108636627 | 2.607407901 | 5.723714538 | 2.47E-06 |
| PSMB4 | 4.011647066 | 1.571678887 | 6.870971663 | 0.008962958 |
| ENSA | 2.825584702 | 1.231189514 | 4.833526392 | 0.018891521 |
| MRPS23 | 3.610293044 | 2.033126241 | 5.380184764 | 0.00023484 |
| INO80C | 2.108913993 | 1.230752962 | 3.194144307 | 0.009098911 |
| PRELID1 | 3.796086637 | 1.694570121 | 6.234452307 | 0.004225259 |
| CAPN1 | 2.441277964 | 1.072495816 | 4.232740581 | 0.0358852 |
| PSMD11 | 4.694742074 | 2.539793685 | 7.019470085 | 0.000125612 |
| MRPL50 | 2.736553023 | 1.30487738 | 4.512245106 | 0.011718542 |
| SET | 5.211684446 | 2.754766663 | 7.827381752 | 0.000129633 |
| SRP72 | 4.29126259 | 2.232822276 | 6.558912849 | 0.000386213 |
| DAP | 3.949295487 | 1.907056719 | 6.264711345 | 0.001602554 |
| MAF1 | 3.436466043 | 1.783438768 | 5.340998296 | 0.001294114 |
| APIP | 2.313943984 | 1.140614108 | 3.817436458 | 0.023453832 |
| CDO1 | 0.695719991 | 0.498556705 | 0.948809724 | 0.020964464 |
| WBP2 | 3.417811305 | 1.083619814 | 6.406607233 | 0.039173458 |
| DUSP23 | 3.02413846 | 1.377616613 | 5.037801394 | 0.010041233 |
| USP1 | 2.826288045 | 1.702604528 | 4.126086219 | 0.00036623 |
| RPL24 | 3.759738996 | 1.415142746 | 6.576255044 | 0.014045041 |
| SPINK1 | 1.300427678 | 1.052959186 | 1.580867935 | 0.015592803 |
| RPLP2 | 3.894097657 | 1.619339393 | 6.551488149 | 0.006702084 |
| CFI | 0.506281978 | 0.239397546 | 0.98482978 | 0.044539554 |
| UGDH | 2.671194573 | 1.514277727 | 4.047008822 | 0.001923348 |
| FTL | 3.871684382 | 1.427308187 | 6.795697191 | 0.014202816 |
| PTTG1 | 2.40244063 | 1.695545654 | 3.202918315 | 1.12E-05 |
| C6 | 0.555594618 | 0.364107852 | 0.822704382 | 0.002640265 |
| NDUFB4 | 4.952649748 | 1.820462653 | 8.474401124 | 0.007001057 |
| CNN3 | 2.875508286 | 1.293291595 | 4.842600544 | 0.014102 |
| ALAS1 | 0.338818835 | 0.13515916 | 0.776958149 | 0.00860884 |
| FAM133A | 1.397002854 | 1.095648097 | 1.742754109 | 0.007930355 |
| POLE4 | 3.623684039 | 2.081191349 | 5.346902719 | 0.000148825 |
| PPIE | 3.755729351 | 2.091127975 | 5.614060884 | 0.000230364 |
| RTN3 | 6.607997588 | 4.221677858 | 9.046789941 | 1.35E-07 |
| PGLS | 2.33076471 | 1.092870764 | 3.939206525 | 0.031335758 |
| RPL35A | 3.397905162 | 1.407894933 | 5.808655478 | 0.011657956 |
| BIRC5 | 2.213138699 | 1.624817963 | 2.877077546 | 5.50E-06 |
| NDUFS2 | 2.981733312 | 1.115943517 | 5.38461496 | 0.033115557 |
| MRPL37 | 6.164788582 | 3.296320989 | 9.150173492 | 6.07E-05 |
| RPS8 | 5.188980053 | 2.710713623 | 7.832535055 | 0.000163701 |
| OPTN | 4.19819732 | 2.093623953 | 6.541838694 | 0.000807949 |
| NACA | 5.133789759 | 2.286537694 | 8.233380959 | 0.001462512 |
| SVIP | 2.160665886 | 1.118926715 | 3.495985291 | 0.024691472 |
| TIPRL | 3.931035672 | 1.958322651 | 6.157649306 | 0.001111357 |
| ATP6V0B | 6.549563817 | 3.973575058 | 9.191941493 | 1.42E-06 |
| ASPH | 1.795398663 | 1.161276321 | 2.570037896 | 0.010435811 |
| EIF3M | 4.847446819 | 2.526800005 | 7.349910721 | 0.000220373 |
| PSMC3 | 4.643220009 | 1.638309348 | 8.094588858 | 0.010362345 |
| CACYBP | 3.715882219 | 2.182835593 | 5.413683977 | 7.16E-05 |
| HSPD1 | 6.609623089 | 3.557775842 | 9.761462406 | 4.43E-05 |
| NDUFB5 | 4.273112269 | 1.669514631 | 7.277282812 | 0.00740905 |
| PSMA5 | 5.287572187 | 2.751153206 | 7.9868303 | 0.000164611 |
| TIMM8B | 3.662665948 | 1.578856537 | 6.11808183 | 0.006516627 |
| RPN2 | 5.700949579 | 2.686656109 | 8.90577379 | 0.000576284 |
| UBE2V2 | 2.978937664 | 1.258245928 | 5.131076065 | 0.018003116 |
| MRPL11 | 3.919998867 | 1.897152179 | 6.216191043 | 0.001614612 |
| PTDSS1 | 3.257721457 | 1.624980876 | 5.17761338 | 0.002895527 |
| ATP6V0E1 | 4.781903892 | 1.787967856 | 8.167284947 | 0.006955374 |
| SSNA1 | 3.737336804 | 1.823572712 | 5.92907648 | 0.001835371 |
| SSR2 | 3.208655742 | 1.789157185 | 4.84181969 | 0.000639487 |
| FIBP | 5.002446775 | 2.566796572 | 7.620691439 | 0.000255284 |
| PSMD2 | 5.316751851 | 2.991792214 | 7.768845285 | 3.05E-05 |
| SLC35B1 | 3.278579583 | 1.230973343 | 5.838766768 | 0.02302082 |
| TMEM98 | 1.670875828 | 1.147077741 | 2.300775399 | 0.00906674 |
| CHCHD3 | 3.572871539 | 1.801654149 | 5.608411047 | 0.001536435 |
| NRBP1 | 4.566826454 | 2.401769715 | 6.922080016 | 0.000242265 |
| PIPOX | 0.662841651 | 0.431509684 | 0.98225456 | 0.039949934 |
| DDX49 | 3.724488593 | 1.891305029 | 5.809809001 | 0.001125203 |
| SF3B2 | 4.753616332 | 2.13989381 | 7.636878682 | 0.001673101 |
| MRPS15 | 5.977670839 | 3.149182973 | 8.935492564 | 8.89E-05 |
| SLC22A7 | 0.665584633 | 0.508493057 | 0.858565589 | 0.001384784 |
| TXNRD1 | 2.516759398 | 1.571081064 | 3.622423159 | 0.000497043 |
| SHARPIN | 2.970772234 | 1.403667072 | 4.878958832 | 0.008159506 |
| TMEM126B | 4.038244503 | 1.859615874 | 6.516567824 | 0.002440992 |
| BCAS2 | 4.188096531 | 1.859149108 | 6.83217944 | 0.003006488 |
| TK1 | 2.204693653 | 1.42140695 | 3.131472195 | 0.001018108 |
| RPL29 | 3.656275087 | 1.594917603 | 6.080679568 | 0.005984141 |
| SLC25A5 | 3.031513318 | 1.109595512 | 5.507252247 | 0.034223197 |
| PARK7 | 3.53247555 | 1.064781417 | 6.69140281 | 0.041767506 |
| HSPA5 | 3.277004825 | 1.264858637 | 5.77783058 | 0.020132988 |
| POLR2K | 3.188379203 | 1.356842046 | 5.432594229 | 0.012786422 |
| TRAPPC3 | 7.179457151 | 4.223941818 | 10.19557564 | 3.39E-06 |
| SCAMP3 | 4.061705317 | 2.148636598 | 6.185271443 | 0.00038381 |
| COMMD5 | 3.082233439 | 1.56249312 | 4.885417475 | 0.003295987 |
| ENAH | 2.027322382 | 1.297477748 | 2.905277653 | 0.003181438 |
| TMEM126A | 6.487369457 | 3.619483048 | 9.447416749 | 2.00E-05 |
| CLTA | 5.968423304 | 3.484438218 | 8.544849238 | 7.16E-06 |
| PSMD12 | 4.532797211 | 2.108562668 | 7.219910455 | 0.001395865 |
| ATP6V1C1 | 2.687457833 | 1.377780664 | 4.287125435 | 0.006714447 |
| EPB41L2 | 2.074772663 | 1.334954868 | 2.959552674 | 0.002186368 |
| IMP4 | 4.516759295 | 1.864284314 | 7.51155121 | 0.004237921 |
| CCDC34 | 2.706721311 | 1.769515547 | 3.774507329 | 5.45E-05 |
| RPL22 | 2.661788057 | 1.051734512 | 4.779125272 | 0.0407405 |
| RRS1 | 1.925493293 | 1.072377489 | 3.018241014 | 0.030303292 |
| CCT8 | 5.590006829 | 2.914683201 | 8.414288222 | 0.000131811 |
| C17orf58 | 3.13316038 | 1.663044376 | 4.852379753 | 0.001653108 |
| VPS25 | 3.827722112 | 1.71677426 | 6.270078902 | 0.003915049 |
| SSBP1 | 5.843665668 | 3.129012717 | 8.686259936 | 6.86E-05 |
| MRPL13 | 2.487014389 | 1.117566961 | 4.258290621 | 0.028943762 |
| DNAJB4 | 2.545636231 | 1.603531362 | 3.641911577 | 0.000349491 |
| PRCC | 3.669053882 | 1.898741121 | 5.681932586 | 0.000932938 |
| CTAG2 | 1.350023409 | 1.119070825 | 1.60766289 | 0.002101094 |
| PSMD13 | 5.555391004 | 2.980153103 | 8.268797897 | 7.75E-05 |
| SSR3 | 5.477350084 | 3.081633757 | 7.994579926 | 2.74E-05 |
| PABPC1 | 3.364511781 | 1.645869653 | 5.378996111 | 0.003028039 |
| CYP2A6 | 0.783240836 | 0.631204718 | 0.961336412 | 0.018754709 |
| PDLIM3 | 1.741716683 | 1.179960814 | 2.417418586 | 0.006743873 |
| PTGES3 | 5.325796327 | 2.395220777 | 8.49170399 | 0.001189525 |
| RPS6KB2 | 2.564911361 | 1.022434652 | 4.611402274 | 0.045641555 |
| LMO4 | 3.459143385 | 2.134526077 | 4.929672073 | 3.00E-05 |
| SSU72 | 4.915152338 | 2.230528464 | 7.854484534 | 0.001379493 |
| PPP1R1A | 0.746317263 | 0.556263106 | 0.982348256 | 0.036413325 |
| PLOD1 | 4.201112602 | 2.210567109 | 6.398727466 | 0.000354597 |
| C9orf16 | 2.24498423 | 1.199530268 | 3.559411361 | 0.014663439 |
| APH1A | 4.198703287 | 1.80484121 | 6.92931067 | 0.003939144 |
| MMP1 | 2.166774749 | 1.677273709 | 2.709611118 | 1.16E-07 |
| CALU | 3.299028859 | 1.878101139 | 4.917435818 | 0.000351636 |
| HNRNPK | 4.950268449 | 2.102631864 | 8.090720807 | 0.002533103 |
| ACSM2A | 0.664979499 | 0.481049982 | 0.900215468 | 0.007410931 |
| COMMD9 | 4.291563664 | 2.535434001 | 6.189511596 | 2.34E-05 |
| MAGOH | 4.887102698 | 2.464649376 | 7.506667066 | 0.000366924 |
| SNRPC | 4.445425462 | 2.171654985 | 6.957359118 | 0.000834628 |
| PCNA | 3.352689956 | 1.854722823 | 5.064096059 | 0.000545021 |
| CCDC47 | 2.747677854 | 1.206316627 | 4.700004287 | 0.02054974 |
| DNAJC7 | 4.670041546 | 2.524870532 | 6.986369062 | 0.000130232 |
| MEAF6 | 2.261535741 | 1.106395517 | 3.753846222 | 0.028213899 |
| RPL27A | 2.944794971 | 1.179672224 | 5.188855096 | 0.025311231 |
| AMT | 6.678420027 | 1.840416824 | 11.96088725 | 0.013654902 |
| RSL1D1 | 4.772683469 | 2.334800028 | 7.430149067 | 0.000621815 |
| SLC25A39 | 6.040142373 | 3.293215764 | 8.901135055 | 4.21E-05 |
| SPARCL1 | 0.594417288 | 0.361698985 | 0.935337367 | 0.023268492 |
| SNX17 | 6.032143368 | 2.856450548 | 9.379941818 | 0.000469538 |
| BTF3 | 3.546264557 | 1.247730794 | 6.392511163 | 0.023564904 |
| TNIP1 | 2.993483232 | 1.253035682 | 5.172144684 | 0.01859444 |
| SNAPIN | 3.722136254 | 1.698000606 | 6.072108425 | 0.003778269 |
| OAZ1 | 4.471924323 | 1.973399135 | 7.270792648 | 0.00250083 |
| C8orf33 | 3.176782485 | 1.737322658 | 4.843662369 | 0.000956983 |
| HNRNPA3 | 4.362701802 | 2.282371079 | 6.645729975 | 0.000326536 |
| ARMC1 | 2.714501772 | 1.159111344 | 4.706027204 | 0.025572529 |

Tab S4. The details of GO enrichment analysis.

| ONTOLOGY | ID | Description | pvalue | Count |
| --- | --- | --- | --- | --- |
| BP | GO:0000280 | nuclear division | 3.14E-26 | 81 |
| BP | GO:0048285 | organelle fission | 2.23E-24 | 83 |
| BP | GO:0140014 | mitotic nuclear division | 2.72E-23 | 61 |
| BP | GO:0000070 | mitotic sister chromatid segregation | 4.96E-20 | 43 |
| BP | GO:0000819 | sister chromatid segregation | 6.43E-20 | 47 |
| BP | GO:0007059 | chromosome segregation | 1.27E-19 | 62 |
| BP | GO:0098813 | nuclear chromosome segregation | 1.20E-18 | 54 |
| BP | GO:1903046 | meiotic cell cycle process | 6.85E-14 | 39 |
| BP | GO:0051783 | regulation of nuclear division | 7.62E-14 | 32 |
| BP | GO:0051321 | meiotic cell cycle | 2.60E-13 | 44 |
| BP | GO:0140013 | meiotic nuclear division | 4.94E-13 | 36 |
| BP | GO:0051983 | regulation of chromosome segregation | 5.77E-13 | 25 |
| BP | GO:0006260 | DNA replication | 9.56E-13 | 43 |
| BP | GO:0010965 | regulation of mitotic sister chromatid separation | 1.26E-12 | 21 |
| BP | GO:0007088 | regulation of mitotic nuclear division | 1.32E-12 | 27 |
| BP | GO:0033045 | regulation of sister chromatid segregation | 1.32E-12 | 22 |
| BP | GO:0044772 | mitotic cell cycle phase transition | 1.63E-12 | 57 |
| BP | GO:0051306 | mitotic sister chromatid separation | 2.46E-12 | 21 |
| BP | GO:0007091 | metaphase/anaphase transition of mitotic cell cycle | 4.49E-12 | 20 |
| BP | GO:1905818 | regulation of chromosome separation | 1.17E-11 | 21 |
| BP | GO:0044784 | metaphase/anaphase transition of cell cycle | 1.20E-11 | 20 |
| BP | GO:1902850 | microtubule cytoskeleton organization involved in mitosis | 1.23E-11 | 30 |
| BP | GO:0030071 | regulation of mitotic metaphase/anaphase transition | 2.23E-11 | 19 |
| BP | GO:0051784 | negative regulation of nuclear division | 5.66E-11 | 18 |
| BP | GO:1902099 | regulation of metaphase/anaphase transition of cell cycle | 5.83E-11 | 19 |
| BP | GO:0007052 | mitotic spindle organization | 7.10E-11 | 26 |
| BP | GO:0007346 | regulation of mitotic cell cycle | 1.03E-10 | 56 |
| BP | GO:0051304 | chromosome separation | 1.03E-10 | 23 |
| BP | GO:0045839 | negative regulation of mitotic nuclear division | 3.59E-10 | 16 |
| BP | GO:1901990 | regulation of mitotic cell cycle phase transition | 3.74E-10 | 42 |
| BP | GO:0033046 | negative regulation of sister chromatid segregation | 6.16E-10 | 15 |
| BP | GO:0033048 | negative regulation of mitotic sister chromatid segregation | 6.16E-10 | 15 |
| BP | GO:2000816 | negative regulation of mitotic sister chromatid separation | 6.16E-10 | 15 |
| BP | GO:0006261 | DNA-dependent DNA replication | 6.19E-10 | 28 |
| BP | GO:0090068 | positive regulation of cell cycle process | 6.99E-10 | 36 |
| BP | GO:0007051 | spindle organization | 9.03E-10 | 31 |
| BP | GO:0051985 | negative regulation of chromosome segregation | 1.28E-09 | 15 |
| BP | GO:1905819 | negative regulation of chromosome separation | 1.28E-09 | 15 |
| BP | GO:0033044 | regulation of chromosome organization | 1.36E-09 | 31 |
| BP | GO:0033047 | regulation of mitotic sister chromatid segregation | 1.82E-09 | 15 |
| BP | GO:0007093 | mitotic cell cycle checkpoint signaling | 1.97E-09 | 25 |
| BP | GO:1901987 | regulation of cell cycle phase transition | 1.99E-09 | 48 |
| BP | GO:0045841 | negative regulation of mitotic metaphase/anaphase transition | 2.20E-09 | 14 |
| BP | GO:0051383 | kinetochore organization | 2.29E-09 | 11 |
| BP | GO:1902100 | negative regulation of metaphase/anaphase transition of cell cycle | 4.57E-09 | 14 |
| BP | GO:0010948 | negative regulation of cell cycle process | 8.25E-09 | 39 |
| BP | GO:0044839 | cell cycle G2/M phase transition | 8.29E-09 | 26 |
| BP | GO:0007094 | mitotic spindle assembly checkpoint signaling | 1.14E-08 | 13 |
| BP | GO:0071173 | spindle assembly checkpoint signaling | 1.14E-08 | 13 |
| BP | GO:0071174 | mitotic spindle checkpoint signaling | 1.14E-08 | 13 |
| BP | GO:0045787 | positive regulation of cell cycle | 1.55E-08 | 40 |
| BP | GO:0031577 | spindle checkpoint signaling | 1.64E-08 | 13 |
| BP | GO:2001251 | negative regulation of chromosome organization | 1.83E-08 | 19 |
| BP | GO:0044786 | cell cycle DNA replication | 3.25E-08 | 13 |
| BP | GO:0000086 | G2/M transition of mitotic cell cycle | 3.27E-08 | 24 |
| BP | GO:0000075 | cell cycle checkpoint signaling | 3.49E-08 | 27 |
| BP | GO:1902749 | regulation of cell cycle G2/M phase transition | 6.57E-08 | 20 |
| BP | GO:0034508 | centromere complex assembly | 6.78E-08 | 11 |
| BP | GO:1902751 | positive regulation of cell cycle G2/M phase transition | 6.78E-08 | 11 |
| BP | GO:0010389 | regulation of G2/M transition of mitotic cell cycle | 8.39E-08 | 19 |
| BP | GO:0045930 | negative regulation of mitotic cell cycle | 9.73E-08 | 32 |
| BP | GO:0061982 | meiosis I cell cycle process | 1.07E-07 | 23 |
| BP | GO:1901991 | negative regulation of mitotic cell cycle phase transition | 1.19E-07 | 27 |
| BP | GO:0010971 | positive regulation of G2/M transition of mitotic cell cycle | 2.43E-07 | 10 |
| BP | GO:0006270 | DNA replication initiation | 2.98E-07 | 11 |
| BP | GO:0051445 | regulation of meiotic cell cycle | 3.39E-07 | 13 |
| BP | GO:0045132 | meiotic chromosome segregation | 3.64E-07 | 18 |
| BP | GO:1901992 | positive regulation of mitotic cell cycle phase transition | 3.64E-07 | 18 |
| BP | GO:0008608 | attachment of spindle microtubules to kinetochore | 4.15E-07 | 11 |
| BP | GO:0030198 | extracellular matrix organization | 4.24E-07 | 36 |
| BP | GO:0007080 | mitotic metaphase plate congression | 4.38E-07 | 13 |
| BP | GO:0043062 | extracellular structure organization | 4.60E-07 | 36 |
| BP | GO:1901989 | positive regulation of cell cycle phase transition | 5.10E-07 | 20 |
| BP | GO:0045229 | external encapsulating structure organization | 5.40E-07 | 36 |
| BP | GO:0045786 | negative regulation of cell cycle | 5.93E-07 | 42 |
| BP | GO:1904029 | regulation of cyclin-dependent protein kinase activity | 8.20E-07 | 18 |
| BP | GO:0007127 | meiosis I | 8.30E-07 | 21 |
| BP | GO:1901988 | negative regulation of cell cycle phase transition | 1.13E-06 | 31 |
| BP | GO:0051302 | regulation of cell division | 1.22E-06 | 25 |
| BP | GO:0071459 | protein localization to chromosome, centromeric region | 1.30E-06 | 9 |
| BP | GO:0006302 | double-strand break repair | 1.34E-06 | 31 |
| BP | GO:1902969 | mitotic DNA replication | 1.44E-06 | 7 |
| BP | GO:0051310 | metaphase plate congression | 1.89E-06 | 14 |
| BP | GO:0090307 | mitotic spindle assembly | 1.89E-06 | 14 |
| BP | GO:0000079 | regulation of cyclin-dependent protein serine/threonine kinase activity | 2.06E-06 | 17 |
| BP | GO:0006268 | DNA unwinding involved in DNA replication | 2.60E-06 | 7 |
| BP | GO:0045931 | positive regulation of mitotic cell cycle | 4.75E-06 | 19 |
| BP | GO:0051303 | establishment of chromosome localization | 5.13E-06 | 15 |
| BP | GO:0009410 | response to xenobiotic stimulus | 5.23E-06 | 45 |
| BP | GO:0007143 | female meiotic nuclear division | 5.37E-06 | 9 |
| BP | GO:0033260 | nuclear DNA replication | 6.47E-06 | 10 |
| BP | GO:0140694 | non-membrane-bounded organelle assembly | 6.99E-06 | 38 |
| BP | GO:0050000 | chromosome localization | 7.04E-06 | 15 |
| BP | GO:0015711 | organic anion transport | 7.44E-06 | 37 |
| BP | GO:0000727 | double-strand break repair via break-induced replication | 8.57E-06 | 6 |
| BP | GO:0000724 | double-strand break repair via homologous recombination | 9.39E-06 | 20 |
| BP | GO:0051382 | kinetochore assembly | 1.13E-05 | 7 |
| BP | GO:0000725 | recombinational repair | 1.17E-05 | 20 |
| BP | GO:0033674 | positive regulation of kinase activity | 1.53E-05 | 44 |
| BP | GO:0001655 | urogenital system development | 1.59E-05 | 35 |
| BP | GO:0034501 | protein localization to kinetochore | 1.72E-05 | 7 |
| BP | GO:1903083 | protein localization to condensed chromosome | 1.72E-05 | 7 |
| BP | GO:0070192 | chromosome organization involved in meiotic cell cycle | 2.05E-05 | 13 |
| BP | GO:0032392 | DNA geometric change | 2.26E-05 | 15 |
| BP | GO:0010639 | negative regulation of organelle organization | 2.95E-05 | 35 |
| BP | GO:0022617 | extracellular matrix disassembly | 3.79E-05 | 12 |
| BP | GO:0051315 | attachment of mitotic spindle microtubules to kinetochore | 4.10E-05 | 6 |
| BP | GO:0051225 | spindle assembly | 4.17E-05 | 17 |
| BP | GO:0032508 | DNA duplex unwinding | 4.21E-05 | 14 |
| BP | GO:0045860 | positive regulation of protein kinase activity | 4.99E-05 | 37 |
| BP | GO:0046942 | carboxylic acid transport | 5.25E-05 | 29 |
| BP | GO:0006310 | DNA recombination | 6.80E-05 | 31 |
| BP | GO:0030261 | chromosome condensation | 7.53E-05 | 10 |
| BP | GO:0001822 | kidney development | 7.75E-05 | 30 |
| BP | GO:0044771 | meiotic cell cycle phase transition | 9.05E-05 | 5 |
| BP | GO:0007076 | mitotic chromosome condensation | 9.33E-05 | 6 |
| BP | GO:2000027 | regulation of animal organ morphogenesis | 9.73E-05 | 17 |
| BP | GO:2001242 | regulation of intrinsic apoptotic signaling pathway | 0.000116473 | 20 |
| BP | GO:0034502 | protein localization to chromosome | 0.000117273 | 14 |
| BP | GO:0040020 | regulation of meiotic nuclear division | 0.00012811 | 8 |
| BP | GO:0007292 | female gamete generation | 0.000128138 | 18 |
| BP | GO:0072001 | renal system development | 0.000133588 | 30 |
| BP | GO:0051782 | negative regulation of cell division | 0.000134335 | 6 |
| BP | GO:0015849 | organic acid transport | 0.000141663 | 30 |
| BP | GO:0007565 | female pregnancy | 0.000148233 | 22 |
| BP | GO:0071103 | DNA conformation change | 0.000153073 | 29 |
| BP | GO:0006275 | regulation of DNA replication | 0.000174785 | 15 |
| BP | GO:0045143 | homologous chromosome segregation | 0.000179636 | 11 |
| BP | GO:0001701 | in utero embryonic development | 0.000191063 | 34 |
| BP | GO:0044774 | mitotic DNA integrity checkpoint signaling | 0.000193265 | 13 |
| BP | GO:2000779 | regulation of double-strand break repair | 0.000193265 | 13 |
| BP | GO:0034446 | substrate adhesion-dependent cell spreading | 0.000194155 | 15 |
| BP | GO:0001558 | regulation of cell growth | 0.000209208 | 37 |
| BP | GO:0048736 | appendage development | 0.000223319 | 20 |
| BP | GO:0060173 | limb development | 0.000223319 | 20 |
| BP | GO:0001833 | inner cell mass cell proliferation | 0.000232723 | 5 |
| BP | GO:0045741 | positive regulation of epidermal growth factor-activated receptor activity | 0.000232723 | 5 |
| BP | GO:0051988 | regulation of attachment of spindle microtubules to kinetochore | 0.000232723 | 5 |
| BP | GO:0060707 | trophoblast giant cell differentiation | 0.000232723 | 5 |
| BP | GO:0016331 | morphogenesis of embryonic epithelium | 0.000238493 | 18 |
| BP | GO:0045933 | positive regulation of muscle contraction | 0.000238568 | 9 |
| BP | GO:0098657 | import into cell | 0.000241764 | 24 |
| BP | GO:0045742 | positive regulation of epidermal growth factor receptor signaling pathway | 0.000245787 | 8 |
| BP | GO:0061098 | positive regulation of protein tyrosine kinase activity | 0.000248704 | 10 |
| BP | GO:0031570 | DNA integrity checkpoint signaling | 0.000260449 | 16 |
| BP | GO:0060249 | anatomical structure homeostasis | 0.000264004 | 30 |
| BP | GO:0016049 | cell growth | 0.000278383 | 41 |
| BP | GO:0051656 | establishment of organelle localization | 0.000284056 | 35 |
| BP | GO:0018108 | peptidyl-tyrosine phosphorylation | 0.000285779 | 34 |
| BP | GO:0042063 | gliogenesis | 0.000288502 | 29 |
| BP | GO:0090175 | regulation of establishment of planar polarity | 0.00028995 | 10 |
| BP | GO:0018212 | peptidyl-tyrosine modification | 0.000330904 | 34 |
| BP | GO:0045989 | positive regulation of striated muscle contraction | 0.000347771 | 5 |
| BP | GO:1901186 | positive regulation of ERBB signaling pathway | 0.000364894 | 8 |
| BP | GO:0044706 | multi-multicellular organism process | 0.000379457 | 23 |
| BP | GO:0060560 | developmental growth involved in morphogenesis | 0.000379652 | 24 |
| BP | GO:0051960 | regulation of nervous system development | 0.000390193 | 38 |
| BP | GO:0050731 | positive regulation of peptidyl-tyrosine phosphorylation | 0.000393106 | 21 |
| BP | GO:0002673 | regulation of acute inflammatory response | 0.000397 | 9 |
| BP | GO:0010810 | regulation of cell-substrate adhesion | 0.000404837 | 23 |
| BP | GO:0062197 | cellular response to chemical stress | 0.000406782 | 31 |
| BP | GO:0014706 | striated muscle tissue development | 0.000440597 | 34 |
| BP | GO:0042692 | muscle cell differentiation | 0.000440597 | 34 |
| BP | GO:2001021 | negative regulation of response to DNA damage stimulus | 0.000455464 | 12 |
| BP | GO:0060326 | cell chemotaxis | 0.000469508 | 29 |
| BP | GO:0060993 | kidney morphogenesis | 0.000477446 | 13 |
| BP | GO:0035051 | cardiocyte differentiation | 0.00049591 | 18 |
| BP | GO:0071474 | cellular hyperosmotic response | 0.00050116 | 5 |
| BP | GO:0000281 | mitotic cytokinesis | 0.00052723 | 11 |
| BP | GO:0051146 | striated muscle cell differentiation | 0.000536071 | 27 |
| BP | GO:0043268 | positive regulation of potassium ion transport | 0.000544402 | 9 |
| BP | GO:1904666 | regulation of ubiquitin protein ligase activity | 0.000594701 | 6 |
| BP | GO:0001736 | establishment of planar polarity | 0.000595837 | 11 |
| BP | GO:0007164 | establishment of tissue polarity | 0.000595837 | 11 |
| BP | GO:0035567 | non-canonical Wnt signaling pathway | 0.000595837 | 11 |
| BP | GO:0042770 | signal transduction in response to DNA damage | 0.000605463 | 19 |
| BP | GO:0071470 | cellular response to osmotic stress | 0.000627844 | 8 |
| BP | GO:0000910 | cytokinesis | 0.000650379 | 19 |
| BP | GO:0048608 | reproductive structure development | 0.000662373 | 36 |
| BP | GO:0003338 | metanephros morphogenesis | 0.000664889 | 7 |
| BP | GO:0001838 | embryonic epithelial tube formation | 0.000672669 | 15 |
| BP | GO:0007062 | sister chromatid cohesion | 0.000675652 | 10 |
| BP | GO:0097193 | intrinsic apoptotic signaling pathway | 0.000701642 | 27 |
| BP | GO:0060071 | Wnt signaling pathway, planar cell polarity pathway | 0.000733878 | 9 |
| BP | GO:0061458 | reproductive system development | 0.000752784 | 36 |
| BP | GO:2001243 | negative regulation of intrinsic apoptotic signaling pathway | 0.000793574 | 13 |
| BP | GO:0007566 | embryo implantation | 0.000847007 | 9 |
| BP | GO:0098739 | import across plasma membrane | 0.000859963 | 19 |
| BP | GO:1903825 | organic acid transmembrane transport | 0.000861482 | 17 |
| BP | GO:2001020 | regulation of response to DNA damage stimulus | 0.000862483 | 22 |
| BP | GO:0036293 | response to decreased oxygen levels | 0.000862633 | 29 |
| BP | GO:0001666 | response to hypoxia | 0.000866037 | 28 |
| BP | GO:0045835 | negative regulation of meiotic nuclear division | 0.000871252 | 4 |
| BP | GO:1901993 | regulation of meiotic cell cycle phase transition | 0.000871252 | 4 |
| BP | GO:1904668 | positive regulation of ubiquitin protein ligase activity | 0.000871252 | 4 |
| BP | GO:0051153 | regulation of striated muscle cell differentiation | 0.000874314 | 13 |
| BP | GO:0016266 | O-glycan processing | 0.00087556 | 8 |
| BP | GO:0150104 | transport across blood-brain barrier | 0.00087879 | 12 |
| BP | GO:0031346 | positive regulation of cell projection organization | 0.000884184 | 31 |
| BP | GO:0003018 | vascular process in circulatory system | 0.000892628 | 25 |
| BP | GO:0097529 | myeloid leukocyte migration | 0.000916153 | 22 |
| BP | GO:0035107 | appendage morphogenesis | 0.000945793 | 16 |
| BP | GO:0035108 | limb morphogenesis | 0.000945793 | 16 |
| BP | GO:0000076 | DNA replication checkpoint signaling | 0.000953281 | 5 |
| BP | GO:0010715 | regulation of extracellular matrix disassembly | 0.000953281 | 5 |
| BP | GO:0060706 | cell differentiation involved in embryonic placenta development | 0.000961218 | 6 |
| BP | GO:0061640 | cytoskeleton-dependent cytokinesis | 0.00096183 | 13 |
| BP | GO:0001843 | neural tube closure | 0.000974094 | 12 |
| BP | GO:0010232 | vascular transport | 0.000974094 | 12 |
| BP | GO:2000780 | negative regulation of double-strand break repair | 0.000977627 | 7 |
| BP | GO:0060537 | muscle tissue development | 0.001029636 | 34 |
| BP | GO:0051781 | positive regulation of cell division | 0.001077841 | 12 |
| BP | GO:0060606 | tube closure | 0.001077841 | 12 |
| BP | GO:0010951 | negative regulation of endopeptidase activity | 0.001091182 | 24 |
| BP | GO:0001841 | neural tube formation | 0.001158957 | 13 |
| BP | GO:0071900 | regulation of protein serine/threonine kinase activity | 0.00116184 | 31 |
| BP | GO:0001894 | tissue homeostasis | 0.001166546 | 25 |
| BP | GO:0040001 | establishment of mitotic spindle localization | 0.001172168 | 7 |
| BP | GO:0043154 | negative regulation of cysteine-type endopeptidase activity involved in apoptotic process | 0.001181997 | 11 |
| BP | GO:2000241 | regulation of reproductive process | 0.001188159 | 18 |
| BP | GO:0061097 | regulation of protein tyrosine kinase activity | 0.001190597 | 12 |
| BP | GO:0032355 | response to estradiol | 0.001191618 | 16 |
| BP | GO:0001556 | oocyte maturation | 0.001199474 | 6 |
| BP | GO:0046851 | negative regulation of bone remodeling | 0.00126826 | 5 |
| BP | GO:0048535 | lymph node development | 0.00126826 | 5 |
| BP | GO:0051447 | negative regulation of meiotic cell cycle | 0.00126826 | 5 |
| BP | GO:0060749 | mammary gland alveolus development | 0.00126826 | 5 |
| BP | GO:0061377 | mammary gland lobule development | 0.00126826 | 5 |
| BP | GO:0001503 | ossification | 0.001269675 | 34 |
| BP | GO:0051653 | spindle localization | 0.001273941 | 9 |
| BP | GO:0030326 | embryonic limb morphogenesis | 0.001305602 | 14 |
| BP | GO:0035113 | embryonic appendage morphogenesis | 0.001305602 | 14 |
| BP | GO:0007144 | female meiosis I | 0.001317374 | 4 |
| BP | GO:0032329 | serine transport | 0.001317374 | 4 |
| BP | GO:0070601 | centromeric sister chromatid cohesion | 0.001317374 | 4 |
| BP | GO:0090092 | regulation of transmembrane receptor protein serine/threonine kinase signaling pathway | 0.001354042 | 24 |
| BP | GO:0070482 | response to oxygen levels | 0.001358101 | 30 |
| BP | GO:0031589 | cell-substrate adhesion | 0.001386639 | 31 |
| BP | GO:0044818 | mitotic G2/M transition checkpoint | 0.001388068 | 8 |
| BP | GO:0045738 | negative regulation of DNA repair | 0.001395777 | 7 |
| BP | GO:0048568 | embryonic organ development | 0.00144188 | 35 |
| BP | GO:0032465 | regulation of cytokinesis | 0.001445519 | 12 |
| BP | GO:0061005 | cell differentiation involved in kidney development | 0.001449663 | 9 |
| BP | GO:1900024 | regulation of substrate adhesion-dependent cell spreading | 0.001449663 | 9 |
| BP | GO:0001890 | placenta development | 0.001489369 | 16 |
| BP | GO:0001649 | osteoblast differentiation | 0.001543435 | 22 |
| BP | GO:0045927 | positive regulation of growth | 0.001585344 | 24 |
| BP | GO:0035850 | epithelial cell differentiation involved in kidney development | 0.001603735 | 8 |
| BP | GO:0044773 | mitotic DNA damage checkpoint signaling | 0.001617842 | 11 |
| BP | GO:0072175 | epithelial tube formation | 0.001650108 | 15 |
| BP | GO:0032570 | response to progesterone | 0.001651329 | 7 |
| BP | GO:2000042 | negative regulation of double-strand break repair via homologous recombination | 0.00165392 | 5 |
| BP | GO:0001738 | morphogenesis of a polarized epithelium | 0.001743867 | 12 |
| BP | GO:0014020 | primary neural tube formation | 0.001743867 | 12 |
| BP | GO:0001892 | embryonic placenta development | 0.001789369 | 11 |
| BP | GO:0050900 | leukocyte migration | 0.001794393 | 31 |
| BP | GO:0010569 | regulation of double-strand break repair via homologous recombination | 0.001845082 | 8 |
| BP | GO:0015804 | neutral amino acid transport | 0.001845082 | 8 |
| BP | GO:0010466 | negative regulation of peptidase activity | 0.001849721 | 24 |
| BP | GO:0051255 | spindle midzone assembly | 0.001901518 | 4 |
| BP | GO:0061307 | cardiac neural crest cell differentiation involved in heart development | 0.001901518 | 4 |
| BP | GO:0061308 | cardiac neural crest cell development involved in heart development | 0.001901518 | 4 |
| BP | GO:0090177 | establishment of planar polarity involved in neural tube closure | 0.001901518 | 4 |
| BP | GO:2000105 | positive regulation of DNA-dependent DNA replication | 0.001901518 | 4 |
| BP | GO:0051216 | cartilage development | 0.001985502 | 19 |
| BP | GO:0050730 | regulation of peptidyl-tyrosine phosphorylation | 0.00204617 | 24 |
| BP | GO:0010972 | negative regulation of G2/M transition of mitotic cell cycle | 0.002096435 | 9 |
| BP | GO:1990573 | potassium ion import across plasma membrane | 0.002114184 | 8 |
| BP | GO:0001832 | blastocyst growth | 0.002119127 | 5 |
| BP | GO:0003215 | cardiac right ventricle morphogenesis | 0.002119127 | 5 |
| BP | GO:0034104 | negative regulation of tissue remodeling | 0.002119127 | 5 |
| BP | GO:1905039 | carboxylic acid transmembrane transport | 0.002123905 | 16 |
| BP | GO:0071466 | cellular response to xenobiotic stimulus | 0.002139553 | 18 |
| BP | GO:0030330 | DNA damage response, signal transduction by p53 class mediator | 0.002191522 | 10 |
| BP | GO:0042060 | wound healing | 0.00221865 | 34 |
| BP | GO:0032506 | cytokinetic process | 0.002270335 | 7 |
| BP | GO:0051154 | negative regulation of striated muscle cell differentiation | 0.002270335 | 7 |
| BP | GO:0043954 | cellular component maintenance | 0.002356877 | 9 |
| BP | GO:0007586 | digestion | 0.002385371 | 15 |
| BP | GO:0015850 | organic hydroxy compound transport | 0.002583234 | 25 |
| BP | GO:0030307 | positive regulation of cell growth | 0.002604118 | 17 |
| BP | GO:0000132 | establishment of mitotic spindle orientation | 0.002627471 | 6 |
| BP | GO:0007176 | regulation of epidermal growth factor-activated receptor activity | 0.002627471 | 6 |
| BP | GO:0019674 | NAD metabolic process | 0.002627471 | 6 |
| BP | GO:2000117 | negative regulation of cysteine-type endopeptidase activity | 0.002629956 | 11 |
| BP | GO:0045740 | positive regulation of DNA replication | 0.002640093 | 7 |
| BP | GO:0060135 | maternal process involved in female pregnancy | 0.002642368 | 9 |
| BP | GO:0060393 | regulation of pathway-restricted SMAD protein phosphorylation | 0.002642368 | 9 |
| BP | GO:1902750 | negative regulation of cell cycle G2/M phase transition | 0.002642368 | 9 |
| BP | GO:0006000 | fructose metabolic process | 0.002643205 | 4 |
| BP | GO:0097062 | dendritic spine maintenance | 0.002672902 | 5 |
| BP | GO:0051293 | establishment of spindle localization | 0.002744319 | 8 |
| BP | GO:0061138 | morphogenesis of a branching epithelium | 0.002901161 | 18 |
| BP | GO:0051962 | positive regulation of nervous system development | 0.003019638 | 24 |
| BP | GO:0007162 | negative regulation of cell adhesion | 0.00304005 | 26 |
| BP | GO:0051147 | regulation of muscle cell differentiation | 0.003168333 | 16 |
| BP | GO:0019751 | polyol metabolic process | 0.003177313 | 13 |
| BP | GO:0006040 | amino sugar metabolic process | 0.003516566 | 7 |
| BP | GO:0030574 | collagen catabolic process | 0.003516566 | 7 |
| BP | GO:0032467 | positive regulation of cytokinesis | 0.003516566 | 7 |
| BP | GO:0033574 | response to testosterone | 0.003516566 | 7 |
| BP | GO:0000212 | meiotic spindle organization | 0.003561378 | 4 |
| BP | GO:0010771 | negative regulation of cell morphogenesis involved in differentiation | 0.003561378 | 4 |
| BP | GO:0036151 | phosphatidylcholine acyl-chain remodeling | 0.003561378 | 4 |
| BP | GO:0051984 | positive regulation of chromosome segregation | 0.003561378 | 4 |
| BP | GO:1900025 | negative regulation of substrate adhesion-dependent cell spreading | 0.003561378 | 4 |
| BP | GO:0030595 | leukocyte chemotaxis | 0.003585678 | 21 |
| BP | GO:0006865 | amino acid transport | 0.003606598 | 15 |
| BP | GO:0060389 | pathway-restricted SMAD protein phosphorylation | 0.003665846 | 9 |
| BP | GO:1902475 | L-alpha-amino acid transmembrane transport | 0.003665846 | 9 |
| BP | GO:0048732 | gland development | 0.003725515 | 34 |
| BP | GO:0065004 | protein-DNA complex assembly | 0.003734687 | 19 |
| BP | GO:0046849 | bone remodeling | 0.003763772 | 11 |
| BP | GO:0045785 | positive regulation of cell adhesion | 0.003860424 | 34 |
| BP | GO:0060562 | epithelial tube morphogenesis | 0.003987694 | 27 |
| BP | GO:1901381 | positive regulation of potassium ion transmembrane transport | 0.004030095 | 7 |
| BP | GO:0071300 | cellular response to retinoic acid | 0.004068466 | 9 |
| BP | GO:0051446 | positive regulation of meiotic cell cycle | 0.004082584 | 5 |
| BP | GO:0022600 | digestive system process | 0.004088481 | 12 |
| BP | GO:0072080 | nephron tubule development | 0.00410076 | 11 |
| BP | GO:0030308 | negative regulation of cell growth | 0.004100762 | 18 |
| BP | GO:0042445 | hormone metabolic process | 0.004181247 | 20 |
| BP | GO:0022612 | gland morphogenesis | 0.004284426 | 13 |
| BP | GO:0051052 | regulation of DNA metabolic process | 0.004341303 | 29 |
| BP | GO:0048771 | tissue remodeling | 0.004487107 | 17 |
| BP | GO:0006271 | DNA strand elongation involved in DNA replication | 0.004674185 | 4 |
| BP | GO:0018146 | keratan sulfate biosynthetic process | 0.004674185 | 4 |
| BP | GO:0032328 | alanine transport | 0.004674185 | 4 |
| BP | GO:0042249 | establishment of planar polarity of embryonic epithelium | 0.004674185 | 4 |
| BP | GO:0048738 | cardiac muscle tissue development | 0.004827637 | 21 |
| BP | GO:0032528 | microvillus organization | 0.004956666 | 5 |
| BP | GO:0010812 | negative regulation of cell-substrate adhesion | 0.00497674 | 9 |
| BP | GO:0051148 | negative regulation of muscle cell differentiation | 0.00497674 | 9 |
| BP | GO:0035148 | tube formation | 0.004978702 | 15 |
| BP | GO:0097530 | granulocyte migration | 0.004978702 | 15 |
| BP | GO:0061448 | connective tissue development | 0.004985263 | 22 |
| BP | GO:0052547 | regulation of peptidase activity | 0.004998227 | 35 |
| BP | GO:0051346 | negative regulation of hydrolase activity | 0.00502515 | 30 |
| BP | GO:0007095 | mitotic G2 DNA damage checkpoint signaling | 0.005058186 | 6 |
| BP | GO:0010975 | regulation of neuron projection development | 0.005097024 | 34 |
| BP | GO:0001523 | retinoid metabolic process | 0.005222823 | 10 |
| BP | GO:0051180 | vitamin transport | 0.00522522 | 7 |
| BP | GO:2000273 | positive regulation of signaling receptor activity | 0.00522522 | 7 |
| BP | GO:0061326 | renal tubule development | 0.005257886 | 11 |
| BP | GO:0015807 | L-amino acid transport | 0.005486131 | 9 |
| BP | GO:0051785 | positive regulation of nuclear division | 0.005537423 | 8 |
| BP | GO:0007173 | epidermal growth factor receptor signaling pathway | 0.005546736 | 12 |
| BP | GO:1901654 | response to ketone | 0.005682973 | 18 |
| BP | GO:0031109 | microtubule polymerization or depolymerization | 0.005683517 | 13 |
| BP | GO:1990266 | neutrophil migration | 0.005683517 | 13 |
| BP | GO:0010765 | positive regulation of sodium ion transport | 0.00586219 | 6 |
| BP | GO:0007019 | microtubule depolymerization | 0.00591394 | 7 |
| BP | GO:0090329 | regulation of DNA-dependent DNA replication | 0.00591394 | 7 |
| BP | GO:0071897 | DNA biosynthetic process | 0.005942745 | 17 |
| BP | GO:0061436 | establishment of skin barrier | 0.005955528 | 5 |
| BP | GO:0072009 | nephron epithelium development | 0.005969092 | 12 |
| BP | GO:0045779 | negative regulation of bone resorption | 0.005998795 | 4 |
| BP | GO:0048385 | regulation of retinoic acid receptor signaling pathway | 0.005998795 | 4 |
| BP | GO:0072283 | metanephric renal vesicle morphogenesis | 0.005998795 | 4 |
| BP | GO:0090231 | regulation of spindle checkpoint | 0.005998795 | 4 |
| BP | GO:0090266 | regulation of mitotic cell cycle spindle assembly checkpoint | 0.005998795 | 4 |
| BP | GO:1903504 | regulation of mitotic spindle checkpoint | 0.005998795 | 4 |
| BP | GO:2000095 | regulation of Wnt signaling pathway, planar cell polarity pathway | 0.005998795 | 4 |
| BP | GO:0034754 | cellular hormone metabolic process | 0.006080644 | 14 |
| BP | GO:0071824 | protein-DNA complex subunit organization | 0.006117118 | 21 |
| BP | GO:0007131 | reciprocal meiotic recombination | 0.006160315 | 8 |
| BP | GO:0140527 | reciprocal homologous recombination | 0.006160315 | 8 |
| BP | GO:0010769 | regulation of cell morphogenesis involved in differentiation | 0.006162717 | 11 |
| BP | GO:0001763 | morphogenesis of a branching structure | 0.006309645 | 18 |
| BP | GO:0021915 | neural tube development | 0.006357713 | 15 |
| BP | GO:0003208 | cardiac ventricle morphogenesis | 0.006624979 | 9 |
| BP | GO:0015718 | monocarboxylic acid transport | 0.006890025 | 12 |
| BP | GO:0043270 | positive regulation of ion transport | 0.006927895 | 23 |
| BP | GO:0043583 | ear development | 0.006939095 | 19 |
| BP | GO:0055007 | cardiac muscle cell differentiation | 0.006954975 | 13 |
| BP | GO:0071621 | granulocyte chemotaxis | 0.006954975 | 13 |
| BP | GO:0006706 | steroid catabolic process | 0.007087921 | 5 |
| BP | GO:2000050 | regulation of non-canonical Wnt signaling pathway | 0.007087921 | 5 |
| BP | GO:0003333 | amino acid transmembrane transport | 0.007185476 | 11 |
| BP | GO:0033627 | cell adhesion mediated by integrin | 0.00725833 | 9 |
| BP | GO:0055001 | muscle cell development | 0.00736516 | 17 |
| BP | GO:0002526 | acute inflammatory response | 0.007390624 | 12 |
| BP | GO:0033628 | regulation of cell adhesion mediated by integrin | 0.007491689 | 7 |
| BP | GO:0048146 | positive regulation of fibroblast proliferation | 0.007491689 | 7 |
| BP | GO:0007096 | regulation of exit from mitosis | 0.007551262 | 4 |
| BP | GO:0042339 | keratan sulfate metabolic process | 0.007551262 | 4 |
| BP | GO:2000696 | regulation of epithelial cell differentiation involved in kidney development | 0.007551262 | 4 |
| BP | GO:0035825 | homologous recombination | 0.007563966 | 8 |
| BP | GO:0022604 | regulation of cell morphogenesis | 0.007572579 | 25 |
| BP | GO:0045216 | cell-cell junction organization | 0.007731629 | 18 |
| BP | GO:0045730 | respiratory burst | 0.007743371 | 6 |
| BP | GO:0051294 | establishment of spindle orientation | 0.007743371 | 6 |
| BP | GO:0042058 | regulation of epidermal growth factor receptor signaling pathway | 0.007936926 | 9 |
| BP | GO:0007517 | muscle organ development | 0.008139964 | 26 |
| BP | GO:0048469 | cell maturation | 0.008146671 | 16 |
| BP | GO:0022411 | cellular component disassembly | 0.00827193 | 33 |
| BP | GO:0046546 | development of primary male sexual characteristics | 0.008279731 | 14 |
| BP | GO:0072006 | nephron development | 0.008279731 | 14 |
| BP | GO:0030199 | collagen fibril organization | 0.008349941 | 8 |
| BP | GO:0090342 | regulation of cell aging | 0.008349941 | 8 |
| BP | GO:0003401 | axis elongation | 0.008362358 | 5 |
| BP | GO:0033561 | regulation of water loss via skin | 0.008362358 | 5 |
| BP | GO:0016101 | diterpenoid metabolic process | 0.008614142 | 10 |
| BP | GO:0006801 | superoxide metabolic process | 0.008662754 | 9 |
| BP | GO:0031023 | microtubule organizing center organization | 0.008786892 | 14 |
| BP | GO:0000077 | DNA damage checkpoint signaling | 0.009065334 | 12 |
| BP | GO:0042471 | ear morphogenesis | 0.009065334 | 12 |
| BP | GO:0051098 | regulation of binding | 0.009188968 | 28 |
| BP | GO:0010720 | positive regulation of cell development | 0.009238059 | 24 |
| BP | GO:0006936 | muscle contraction | 0.009330009 | 27 |
| BP | GO:0002544 | chronic inflammatory response | 0.009346405 | 4 |
| BP | GO:0009404 | toxin metabolic process | 0.009346405 | 4 |
| BP | GO:0030728 | ovulation | 0.009346405 | 4 |
| BP | GO:0061484 | hematopoietic stem cell homeostasis | 0.009346405 | 4 |
| BP | GO:0050767 | regulation of neurogenesis | 0.009516543 | 28 |
| BP | GO:0006282 | regulation of DNA repair | 0.00956476 | 13 |
| BP | GO:0007098 | centrosome cycle | 0.00956476 | 13 |
| BP | GO:0002675 | positive regulation of acute inflammatory response | 0.009787056 | 5 |
| BP | GO:0006972 | hyperosmotic response | 0.009787056 | 5 |
| BP | GO:0061351 | neural precursor cell proliferation | 0.009874593 | 14 |
| BP | GO:0052548 | regulation of endopeptidase activity | 0.009951074 | 32 |
| BP | GO:0016572 | histone phosphorylation | 0.010024041 | 6 |
| BP | GO:0045823 | positive regulation of heart contraction | 0.010024041 | 6 |
| BP | GO:0006735 | NADH regeneration | 0.010209383 | 3 |
| BP | GO:0016081 | synaptic vesicle docking | 0.010209383 | 3 |
| BP | GO:0031055 | chromatin remodeling at centromere | 0.010209383 | 3 |
| BP | GO:0033314 | mitotic DNA replication checkpoint signaling | 0.010209383 | 3 |
| BP | GO:0045843 | negative regulation of striated muscle tissue development | 0.010209383 | 3 |
| BP | GO:0061621 | canonical glycolysis | 0.010209383 | 3 |
| BP | GO:0061718 | glucose catabolic process to pyruvate | 0.010209383 | 3 |
| BP | GO:0090179 | planar cell polarity pathway involved in neural tube closure | 0.010209383 | 3 |
| BP | GO:0030593 | neutrophil chemotaxis | 0.010323719 | 11 |
| BP | GO:0014009 | glial cell proliferation | 0.010413159 | 7 |
| BP | GO:0046850 | regulation of bone remodeling | 0.010413159 | 7 |
| BP | GO:0016311 | dephosphorylation | 0.010541399 | 31 |
| BP | GO:0002028 | regulation of sodium ion transport | 0.010842795 | 10 |
| BP | GO:0048477 | oogenesis | 0.010842795 | 10 |
| BP | GO:0032963 | collagen metabolic process | 0.01106122 | 11 |
| BP | GO:0002274 | myeloid leukocyte activation | 0.011066689 | 19 |
| BP | GO:0030858 | positive regulation of epithelial cell differentiation | 0.011075875 | 8 |
| BP | GO:0046888 | negative regulation of hormone secretion | 0.011075875 | 8 |
| BP | GO:0072593 | reactive oxygen species metabolic process | 0.011215379 | 20 |
| BP | GO:0010863 | positive regulation of phospholipase C activity | 0.01132709 | 6 |
| BP | GO:0031297 | replication fork processing | 0.01132709 | 6 |
| BP | GO:0010458 | exit from mitosis | 0.011369898 | 5 |
| BP | GO:0006007 | glucose catabolic process | 0.011397734 | 4 |
| BP | GO:0006044 | N-acetylglucosamine metabolic process | 0.011397734 | 4 |
| BP | GO:0019373 | epoxygenase P450 pathway | 0.011397734 | 4 |
| BP | GO:0042474 | middle ear morphogenesis | 0.011397734 | 4 |
| BP | GO:0072077 | renal vesicle morphogenesis | 0.011397734 | 4 |
| BP | GO:0010718 | positive regulation of epithelial to mesenchymal transition | 0.011549229 | 7 |
| BP | GO:0043090 | amino acid import | 0.011549229 | 7 |
| BP | GO:0090287 | regulation of cellular response to growth factor stimulus | 0.011619038 | 24 |
| BP | GO:0055006 | cardiac cell development | 0.011675145 | 10 |
| BP | GO:0034599 | cellular response to oxidative stress | 0.011715073 | 23 |
| BP | GO:1903522 | regulation of blood circulation | 0.011766531 | 21 |
| BP | GO:0010976 | positive regulation of neuron projection development | 0.011776267 | 15 |
| BP | GO:0010001 | glial cell differentiation | 0.012089275 | 19 |
| BP | GO:0050769 | positive regulation of neurogenesis | 0.012089275 | 19 |
| BP | GO:0007178 | transmembrane receptor protein serine/threonine kinase signaling pathway | 0.012344865 | 27 |
| BP | GO:0071241 | cellular response to inorganic substance | 0.012627952 | 19 |
| BP | GO:0002062 | chondrocyte differentiation | 0.01265641 | 11 |
| BP | GO:0050892 | intestinal absorption | 0.012745269 | 6 |
| BP | GO:1903524 | positive regulation of blood circulation | 0.012745269 | 6 |
| BP | GO:0008347 | glial cell migration | 0.012772265 | 7 |
| BP | GO:0051438 | regulation of ubiquitin-protein transferase activity | 0.012772265 | 7 |
| BP | GO:2000772 | regulation of cellular senescence | 0.012772265 | 7 |
| BP | GO:1901184 | regulation of ERBB signaling pathway | 0.013070334 | 9 |
| BP | GO:0010669 | epithelial structure maintenance | 0.013118383 | 5 |
| BP | GO:0017145 | stem cell division | 0.013118383 | 5 |
| BP | GO:0001704 | formation of primary germ layer | 0.013288247 | 12 |
| BP | GO:0038127 | ERBB signaling pathway | 0.013288247 | 12 |
| BP | GO:0000018 | regulation of DNA recombination | 0.013516696 | 11 |
| BP | GO:0032526 | response to retinoic acid | 0.013516696 | 11 |
| BP | GO:0006002 | fructose 6-phosphate metabolic process | 0.013543886 | 3 |
| BP | GO:0006971 | hypotonic response | 0.013543886 | 3 |
| BP | GO:0010248 | establishment or maintenance of transmembrane electrochemical gradient | 0.013543886 | 3 |
| BP | GO:0032308 | positive regulation of prostaglandin secretion | 0.013543886 | 3 |
| BP | GO:0048635 | negative regulation of muscle organ development | 0.013543886 | 3 |
| BP | GO:0072697 | protein localization to cell cortex | 0.013543886 | 3 |
| BP | GO:0090178 | regulation of establishment of planar polarity involved in neural tube closure | 0.013543886 | 3 |
| BP | GO:0106049 | regulation of cellular response to osmotic stress | 0.013543886 | 3 |
| BP | GO:1901862 | negative regulation of muscle tissue development | 0.013543886 | 3 |
| BP | GO:0072073 | kidney epithelium development | 0.013642852 | 13 |
| BP | GO:0060231 | mesenchymal to epithelial transition | 0.013717386 | 4 |
| BP | GO:0072087 | renal vesicle development | 0.013717386 | 4 |
| BP | GO:0097150 | neuronal stem cell population maintenance | 0.013717386 | 4 |
| BP | GO:0071456 | cellular response to hypoxia | 0.013780663 | 14 |
| BP | GO:0050808 | synapse organization | 0.013956905 | 31 |
| BP | GO:0019730 | antimicrobial humoral response | 0.014117854 | 12 |
| BP | GO:1900274 | regulation of phospholipase C activity | 0.014283387 | 6 |
| BP | GO:0071695 | anatomical structure maturation | 0.014358735 | 19 |
| BP | GO:0030879 | mammary gland development | 0.014435664 | 13 |
| BP | GO:0045185 | maintenance of protein location | 0.014462825 | 10 |
| BP | GO:0048704 | embryonic skeletal system morphogenesis | 0.014462825 | 10 |
| BP | GO:0034767 | positive regulation of ion transmembrane transport | 0.014463038 | 15 |
| BP | GO:0072330 | monocarboxylic acid biosynthetic process | 0.014854627 | 18 |
| BP | GO:0003231 | cardiac ventricle development | 0.014986291 | 12 |
| BP | GO:0002063 | chondrocyte development | 0.015039595 | 5 |
| BP | GO:0035115 | embryonic forelimb morphogenesis | 0.015039595 | 5 |
| BP | GO:0071168 | protein localization to chromatin | 0.015039595 | 5 |
| BP | GO:0048708 | astrocyte differentiation | 0.01523384 | 9 |
| BP | GO:0006979 | response to oxidative stress | 0.015239887 | 32 |
| BP | GO:0006323 | DNA packaging | 0.015303427 | 17 |
| BP | GO:0042698 | ovulation cycle | 0.015665935 | 8 |
| BP | GO:1904888 | cranial skeletal system development | 0.015665935 | 8 |
| BP | GO:0050878 | regulation of body fluid levels | 0.015683892 | 28 |
| BP | GO:0014002 | astrocyte development | 0.015946082 | 6 |
| BP | GO:0045840 | positive regulation of mitotic nuclear division | 0.015946082 | 6 |
| BP | GO:0006937 | regulation of muscle contraction | 0.015972978 | 15 |
| BP | GO:0021700 | developmental maturation | 0.016000876 | 22 |
| BP | GO:0007063 | regulation of sister chromatid cohesion | 0.016316088 | 4 |
| BP | GO:0031145 | anaphase-promoting complex-dependent catabolic process | 0.016316088 | 4 |
| BP | GO:0033630 | positive regulation of cell adhesion mediated by integrin | 0.016316088 | 4 |
| BP | GO:0070269 | pyroptosis | 0.016316088 | 4 |
| BP | GO:0072311 | glomerular epithelial cell differentiation | 0.016316088 | 4 |
| BP | GO:1902307 | positive regulation of sodium ion transmembrane transport | 0.016316088 | 4 |
| BP | GO:2000774 | positive regulation of cellular senescence | 0.016316088 | 4 |
| BP | GO:0006939 | smooth muscle contraction | 0.016363868 | 11 |
| BP | GO:1904375 | regulation of protein localization to cell periphery | 0.016843988 | 12 |
| BP | GO:0007435 | salivary gland morphogenesis | 0.017140167 | 5 |
| BP | GO:0008156 | negative regulation of DNA replication | 0.017140167 | 5 |
| BP | GO:0006805 | xenobiotic metabolic process | 0.017405991 | 11 |
| BP | GO:0007501 | mesodermal cell fate specification | 0.017424676 | 3 |
| BP | GO:0010454 | negative regulation of cell fate commitment | 0.017424676 | 3 |
| BP | GO:0032306 | regulation of prostaglandin secretion | 0.017424676 | 3 |
| BP | GO:0032490 | detection of molecule of bacterial origin | 0.017424676 | 3 |
| BP | GO:0032536 | regulation of cell projection size | 0.017424676 | 3 |
| BP | GO:0038063 | collagen-activated tyrosine kinase receptor signaling pathway | 0.017424676 | 3 |
| BP | GO:0051639 | actin filament network formation | 0.017424676 | 3 |
| BP | GO:0060394 | negative regulation of pathway-restricted SMAD protein phosphorylation | 0.017424676 | 3 |
| BP | GO:0061620 | glycolytic process through glucose-6-phosphate | 0.017424676 | 3 |
| BP | GO:0072182 | regulation of nephron tubule epithelial cell differentiation | 0.017424676 | 3 |
| BP | GO:0072711 | cellular response to hydroxyurea | 0.017424676 | 3 |
| BP | GO:0048588 | developmental cell growth | 0.017652337 | 19 |
| BP | GO:0006721 | terpenoid metabolic process | 0.01771954 | 10 |
| BP | GO:1990868 | response to chemokine | 0.01771954 | 10 |
| BP | GO:1990869 | cellular response to chemokine | 0.01771954 | 10 |
| BP | GO:0042554 | superoxide anion generation | 0.017737811 | 6 |
| BP | GO:0045124 | regulation of bone resorption | 0.017737811 | 6 |
| BP | GO:0089718 | amino acid import across plasma membrane | 0.017737811 | 6 |
| BP | GO:0008584 | male gonad development | 0.01796395 | 13 |
| BP | GO:0045861 | negative regulation of proteolysis | 0.018802242 | 26 |
| BP | GO:0006970 | response to osmotic stress | 0.018957141 | 9 |
| BP | GO:0022616 | DNA strand elongation | 0.019203141 | 4 |
| BP | GO:0030277 | maintenance of gastrointestinal epithelium | 0.019203141 | 4 |
| BP | GO:0051220 | cytoplasmic sequestering of protein | 0.019203141 | 4 |
| BP | GO:0060444 | branching involved in mammary gland duct morphogenesis | 0.019203141 | 4 |
| BP | GO:2000193 | positive regulation of fatty acid transport | 0.019203141 | 4 |
| BP | GO:2000725 | regulation of cardiac muscle cell differentiation | 0.019203141 | 4 |
| BP | GO:0010469 | regulation of signaling receptor activity | 0.019353833 | 15 |
| BP | GO:0048333 | mesodermal cell differentiation | 0.019426253 | 5 |
| BP | GO:1901976 | regulation of cell cycle checkpoint | 0.019426253 | 5 |
| BP | GO:1904031 | positive regulation of cyclin-dependent protein kinase activity | 0.019426253 | 5 |
| BP | GO:0050727 | regulation of inflammatory response | 0.019491064 | 28 |
| BP | GO:0035272 | exocrine system development | 0.019662825 | 6 |
| BP | GO:0045910 | negative regulation of DNA recombination | 0.019662825 | 6 |
| BP | GO:0048599 | oocyte development | 0.019662825 | 6 |
| BP | GO:0097305 | response to alcohol | 0.019735016 | 20 |
| BP | GO:0001952 | regulation of cell-matrix adhesion | 0.019948708 | 12 |
| BP | GO:0017015 | regulation of transforming growth factor beta receptor signaling pathway | 0.019948708 | 12 |
| BP | GO:0006066 | alcohol metabolic process | 0.020041031 | 26 |
| BP | GO:0007584 | response to nutrient | 0.020278306 | 15 |
| BP | GO:0001656 | metanephros development | 0.020333305 | 9 |
| BP | GO:0055013 | cardiac muscle cell development | 0.020333305 | 9 |
| BP | GO:0060021 | roof of mouth development | 0.020333305 | 9 |
| BP | GO:0007265 | Ras protein signal transduction | 0.020592896 | 25 |
| BP | GO:0008016 | regulation of heart contraction | 0.020768057 | 17 |
| BP | GO:0048863 | stem cell differentiation | 0.020768057 | 17 |
| BP | GO:0090100 | positive regulation of transmembrane receptor protein serine/threonine kinase signaling pathway | 0.020829347 | 11 |
| BP | GO:0034103 | regulation of tissue remodeling | 0.021780069 | 9 |
| BP | GO:0001973 | G protein-coupled adenosine receptor signaling pathway | 0.021859117 | 3 |
| BP | GO:0003337 | mesenchymal to epithelial transition involved in metanephros morphogenesis | 0.021859117 | 3 |
| BP | GO:0010755 | regulation of plasminogen activation | 0.021859117 | 3 |
| BP | GO:0015801 | aromatic amino acid transport | 0.021859117 | 3 |
| BP | GO:0015816 | glycine transport | 0.021859117 | 3 |
| BP | GO:0032530 | regulation of microvillus organization | 0.021859117 | 3 |
| BP | GO:0035588 | G protein-coupled purinergic receptor signaling pathway | 0.021859117 | 3 |
| BP | GO:0036005 | response to macrophage colony-stimulating factor | 0.021859117 | 3 |
| BP | GO:0036006 | cellular response to macrophage colony-stimulating factor stimulus | 0.021859117 | 3 |
| BP | GO:0042659 | regulation of cell fate specification | 0.021859117 | 3 |
| BP | GO:0061615 | glycolytic process through fructose-6-phosphate | 0.021859117 | 3 |
| BP | GO:0070254 | mucus secretion | 0.021859117 | 3 |
| BP | GO:0070486 | leukocyte aggregation | 0.021859117 | 3 |
| BP | GO:0072710 | response to hydroxyurea | 0.021859117 | 3 |
| BP | GO:0007431 | salivary gland development | 0.021903505 | 5 |
| BP | GO:0035137 | hindlimb morphogenesis | 0.021903505 | 5 |
| BP | GO:0042573 | retinoic acid metabolic process | 0.021903505 | 5 |
| BP | GO:0044319 | wound healing, spreading of cells | 0.021903505 | 5 |
| BP | GO:0051973 | positive regulation of telomerase activity | 0.021903505 | 5 |
| BP | GO:0090505 | epiboly involved in wound healing | 0.021903505 | 5 |
| BP | GO:0010038 | response to metal ion | 0.022021094 | 27 |
| BP | GO:0006767 | water-soluble vitamin metabolic process | 0.022122752 | 7 |
| BP | GO:0042476 | odontogenesis | 0.022243013 | 12 |
| BP | GO:0006833 | water transport | 0.022386417 | 4 |
| BP | GO:0032740 | positive regulation of interleukin-17 production | 0.022386417 | 4 |
| BP | GO:0045932 | negative regulation of muscle contraction | 0.022386417 | 4 |
| BP | GO:0036294 | cellular response to decreased oxygen levels | 0.022813975 | 14 |
| BP | GO:0098869 | cellular oxidant detoxification | 0.022863348 | 10 |
| BP | GO:0005996 | monosaccharide metabolic process | 0.02291469 | 20 |
| BP | GO:0044843 | cell cycle G1/S phase transition | 0.023215661 | 19 |
| BP | GO:0030324 | lung development | 0.023252984 | 15 |
| BP | GO:0008544 | epidermis development | 0.023302994 | 24 |
| BP | GO:0021782 | glial cell development | 0.023371504 | 11 |
| BP | GO:0033559 | unsaturated fatty acid metabolic process | 0.023371504 | 11 |
| BP | GO:1990748 | cellular detoxification | 0.023371504 | 11 |
| BP | GO:1903844 | regulation of cellular response to transforming growth factor beta stimulus | 0.023460639 | 12 |
| BP | GO:0043618 | regulation of transcription from RNA polymerase II promoter in response to stress | 0.023928612 | 6 |
| BP | GO:0051972 | regulation of telomerase activity | 0.023928612 | 6 |
| BP | GO:0031638 | zymogen activation | 0.02404561 | 7 |
| BP | GO:0043410 | positive regulation of MAPK cascade | 0.024173244 | 33 |
| BP | GO:0060236 | regulation of mitotic spindle organization | 0.024577058 | 5 |
| BP | GO:0090504 | epiboly | 0.024577058 | 5 |
| BP | GO:1905207 | regulation of cardiocyte differentiation | 0.024577058 | 5 |
| BP | GO:0070098 | chemokine-mediated signaling pathway | 0.02489248 | 9 |
| BP | GO:0046470 | phosphatidylcholine metabolic process | 0.024921505 | 8 |
| BP | GO:0072331 | signal transduction by p53 class mediator | 0.025056033 | 14 |
| BP | GO:0045137 | development of primary sexual characteristics | 0.025322149 | 18 |
| BP | GO:0051235 | maintenance of location | 0.025648775 | 24 |
| BP | GO:0070507 | regulation of microtubule cytoskeleton organization | 0.025653584 | 13 |
| BP | GO:0014013 | regulation of gliogenesis | 0.025805426 | 10 |
| BP | GO:0031639 | plasminogen activation | 0.025872367 | 4 |
| BP | GO:0072010 | glomerular epithelium development | 0.025872367 | 4 |
| BP | GO:0090343 | positive regulation of cell aging | 0.025872367 | 4 |
| BP | GO:0019748 | secondary metabolic process | 0.026080234 | 7 |
| BP | GO:0007613 | memory | 0.026132883 | 11 |
| BP | GO:0051101 | regulation of DNA binding | 0.026132883 | 11 |
| BP | GO:0045005 | DNA-dependent DNA replication maintenance of fidelity | 0.026276744 | 6 |
| BP | GO:0015698 | inorganic anion transport | 0.026543804 | 15 |
| BP | GO:0043627 | response to estrogen | 0.026770305 | 8 |
| BP | GO:0002091 | negative regulation of receptor internalization | 0.026849409 | 3 |
| BP | GO:0007100 | mitotic centrosome separation | 0.026849409 | 3 |
| BP | GO:0033605 | positive regulation of catecholamine secretion | 0.026849409 | 3 |
| BP | GO:0035563 | positive regulation of chromatin binding | 0.026849409 | 3 |
| BP | GO:0035672 | oligopeptide transmembrane transport | 0.026849409 | 3 |
| BP | GO:0043951 | negative regulation of cAMP-mediated signaling | 0.026849409 | 3 |
| BP | GO:0047484 | regulation of response to osmotic stress | 0.026849409 | 3 |
| BP | GO:0051764 | actin crosslink formation | 0.026849409 | 3 |
| BP | GO:0072160 | nephron tubule epithelial cell differentiation | 0.026849409 | 3 |
| BP | GO:0051592 | response to calcium ion | 0.026923798 | 13 |
| BP | GO:0032147 | activation of protein kinase activity | 0.027407562 | 12 |
| BP | GO:0098727 | maintenance of cell number | 0.027407562 | 12 |
| BP | GO:0006699 | bile acid biosynthetic process | 0.027451507 | 5 |
| BP | GO:0043949 | regulation of cAMP-mediated signaling | 0.027451507 | 5 |
| BP | GO:0046661 | male sex differentiation | 0.027457619 | 14 |
| BP | GO:0030323 | respiratory tube development | 0.027714112 | 15 |
| BP | GO:0048638 | regulation of developmental growth | 0.028172031 | 24 |
| BP | GO:0046173 | polyol biosynthetic process | 0.028229199 | 7 |
| BP | GO:0060113 | inner ear receptor cell differentiation | 0.028229199 | 7 |
| BP | GO:0070265 | necrotic cell death | 0.028229199 | 7 |
| BP | GO:0000082 | G1/S transition of mitotic cell cycle | 0.028711483 | 17 |
| BP | GO:0006692 | prostanoid metabolic process | 0.028772856 | 6 |
| BP | GO:0006693 | prostaglandin metabolic process | 0.028772856 | 6 |
| BP | GO:0009994 | oocyte differentiation | 0.028772856 | 6 |
| BP | GO:0010862 | positive regulation of pathway-restricted SMAD protein phosphorylation | 0.028772856 | 6 |
| BP | GO:0048754 | branching morphogenesis of an epithelial tube | 0.029601588 | 13 |
| BP | GO:1904064 | positive regulation of cation transmembrane transport | 0.029601588 | 13 |
| BP | GO:0015874 | norepinephrine transport | 0.02966605 | 4 |
| BP | GO:0051307 | meiotic chromosome separation | 0.02966605 | 4 |
| BP | GO:0060740 | prostate gland epithelium morphogenesis | 0.02966605 | 4 |
| BP | GO:0086013 | membrane repolarization during cardiac muscle cell action potential | 0.02966605 | 4 |
| BP | GO:1903055 | positive regulation of extracellular matrix organization | 0.02966605 | 4 |
| BP | GO:0018105 | peptidyl-serine phosphorylation | 0.029964627 | 23 |
| BP | GO:0022412 | cellular process involved in reproduction in multicellular organism | 0.030365436 | 27 |
| BP | GO:0016202 | regulation of striated muscle tissue development | 0.030530904 | 5 |
| BP | GO:0042178 | xenobiotic catabolic process | 0.030530904 | 5 |
| BP | GO:0048706 | embryonic skeletal system development | 0.030706246 | 11 |
| BP | GO:0001824 | blastocyst development | 0.03071271 | 10 |
| BP | GO:0006766 | vitamin metabolic process | 0.03071271 | 10 |
| BP | GO:0045926 | negative regulation of growth | 0.031111404 | 19 |
| BP | GO:0042572 | retinol metabolic process | 0.031419985 | 6 |
| BP | GO:0090102 | cochlea development | 0.031419985 | 6 |
| BP | GO:0007188 | adenylate cyclase-modulating G protein-coupled receptor signaling pathway | 0.031710841 | 18 |
| BP | GO:0007266 | Rho protein signal transduction | 0.031816069 | 12 |
| BP | GO:0045471 | response to ethanol | 0.031816069 | 12 |
| BP | GO:0002070 | epithelial cell maturation | 0.032393215 | 3 |
| BP | GO:0010940 | positive regulation of necrotic cell death | 0.032393215 | 3 |
| BP | GO:0032310 | prostaglandin secretion | 0.032393215 | 3 |
| BP | GO:0036376 | sodium ion export across plasma membrane | 0.032393215 | 3 |
| BP | GO:0043252 | sodium-independent organic anion transport | 0.032393215 | 3 |
| BP | GO:0045842 | positive regulation of mitotic metaphase/anaphase transition | 0.032393215 | 3 |
| BP | GO:0045986 | negative regulation of smooth muscle contraction | 0.032393215 | 3 |
| BP | GO:0051299 | centrosome separation | 0.032393215 | 3 |
| BP | GO:1901970 | positive regulation of mitotic sister chromatid separation | 0.032393215 | 3 |
| BP | GO:1903799 | negative regulation of production of miRNAs involved in gene silencing by miRNA | 0.032393215 | 3 |
| BP | GO:2000052 | positive regulation of non-canonical Wnt signaling pathway | 0.032393215 | 3 |
| BP | GO:0006940 | regulation of smooth muscle contraction | 0.032879736 | 7 |
| BP | GO:0030104 | water homeostasis | 0.032879736 | 7 |
| BP | GO:0002026 | regulation of the force of heart contraction | 0.033771165 | 4 |
| BP | GO:0034643 | establishment of mitochondrion localization, microtubule-mediated | 0.033771165 | 4 |
| BP | GO:0042634 | regulation of hair cycle | 0.033771165 | 4 |
| BP | GO:0046697 | decidualization | 0.033771165 | 4 |
| BP | GO:0047497 | mitochondrion transport along microtubule | 0.033771165 | 4 |
| BP | GO:0072273 | metanephric nephron morphogenesis | 0.033771165 | 4 |
| BP | GO:1901071 | glucosamine-containing compound metabolic process | 0.033771165 | 4 |
| BP | GO:0035136 | forelimb morphogenesis | 0.033818743 | 5 |
| BP | GO:0071392 | cellular response to estradiol stimulus | 0.033818743 | 5 |
| BP | GO:0048562 | embryonic organ morphogenesis | 0.033872901 | 21 |
| BP | GO:0008202 | steroid metabolic process | 0.033903748 | 23 |
| BP | GO:0051056 | regulation of small GTPase mediated signal transduction | 0.033938945 | 22 |
| BP | GO:0050770 | regulation of axonogenesis | 0.03397393 | 13 |
| BP | GO:0033273 | response to vitamin | 0.034027951 | 9 |
| BP | GO:0006690 | icosanoid metabolic process | 0.034055519 | 11 |
| BP | GO:2000116 | regulation of cysteine-type endopeptidase activity | 0.034085644 | 18 |
| BP | GO:0006636 | unsaturated fatty acid biosynthetic process | 0.034220895 | 6 |
| BP | GO:0048013 | ephrin receptor signaling pathway | 0.034220895 | 6 |
| BP | GO:0006023 | aminoglycan biosynthetic process | 0.035115362 | 8 |
| BP | GO:0060541 | respiratory system development | 0.035212735 | 16 |
| BP | GO:0048762 | mesenchymal cell differentiation | 0.035321068 | 18 |
| BP | GO:0007519 | skeletal muscle tissue development | 0.035529372 | 13 |
| BP | GO:0097237 | cellular response to toxic substance | 0.035823392 | 11 |
| BP | GO:0032414 | positive regulation of ion transmembrane transporter activity | 0.036243986 | 10 |
| BP | GO:0043266 | regulation of potassium ion transport | 0.036243986 | 10 |
| BP | GO:0019318 | hexose metabolic process | 0.036589092 | 18 |
| BP | GO:1901617 | organic hydroxy compound biosynthetic process | 0.036589092 | 18 |
| BP | GO:0016055 | Wnt signaling pathway | 0.037107188 | 30 |
| BP | GO:0007129 | homologous chromosome pairing at meiosis | 0.037178069 | 6 |
| BP | GO:1990138 | neuron projection extension | 0.037203435 | 14 |
| BP | GO:0036297 | interstrand cross-link repair | 0.037317964 | 5 |
| BP | GO:0090224 | regulation of spindle organization | 0.037317964 | 5 |
| BP | GO:1901861 | regulation of muscle tissue development | 0.037317964 | 5 |
| BP | GO:0051053 | negative regulation of DNA metabolic process | 0.037654738 | 11 |
| BP | GO:0014015 | positive regulation of gliogenesis | 0.038014853 | 7 |
| BP | GO:0002825 | regulation of T-helper 1 type immune response | 0.038190088 | 4 |
| BP | GO:0032703 | negative regulation of interleukin-2 production | 0.038190088 | 4 |
| BP | GO:0035116 | embryonic hindlimb morphogenesis | 0.038190088 | 4 |
| BP | GO:0060512 | prostate gland morphogenesis | 0.038190088 | 4 |
| BP | GO:1903203 | regulation of oxidative stress-induced neuron death | 0.038190088 | 4 |
| BP | GO:1903319 | positive regulation of protein maturation | 0.038190088 | 4 |
| BP | GO:1901379 | regulation of potassium ion transmembrane transport | 0.038255927 | 9 |
| BP | GO:0002827 | positive regulation of T-helper 1 type immune response | 0.038484235 | 3 |
| BP | GO:0002921 | negative regulation of humoral immune response | 0.038484235 | 3 |
| BP | GO:0006828 | manganese ion transport | 0.038484235 | 3 |
| BP | GO:0006857 | oligopeptide transport | 0.038484235 | 3 |
| BP | GO:0030033 | microvillus assembly | 0.038484235 | 3 |
| BP | GO:0042481 | regulation of odontogenesis | 0.038484235 | 3 |
| BP | GO:0045651 | positive regulation of macrophage differentiation | 0.038484235 | 3 |
| BP | GO:0060004 | reflex | 0.038484235 | 3 |
| BP | GO:1902101 | positive regulation of metaphase/anaphase transition of cell cycle | 0.038484235 | 3 |
| BP | GO:1903236 | regulation of leukocyte tethering or rolling | 0.038484235 | 3 |
| BP | GO:2000651 | positive regulation of sodium ion transmembrane transporter activity | 0.038484235 | 3 |
| BP | GO:2001224 | positive regulation of neuron migration | 0.038484235 | 3 |
| BP | GO:0046578 | regulation of Ras protein signal transduction | 0.038489128 | 15 |
| BP | GO:0043254 | regulation of protein-containing complex assembly | 0.038683545 | 29 |
| BP | GO:0006694 | steroid biosynthetic process | 0.038775098 | 14 |
| BP | GO:0198738 | cell-cell signaling by wnt | 0.039005879 | 30 |
| BP | GO:0048144 | fibroblast proliferation | 0.039882443 | 8 |
| BP | GO:2000243 | positive regulation of reproductive process | 0.039882443 | 8 |
| BP | GO:0001933 | negative regulation of protein phosphorylation | 0.040193211 | 24 |
| BP | GO:0043620 | regulation of DNA-templated transcription in response to stress | 0.040293704 | 6 |
| BP | GO:0045744 | negative regulation of G protein-coupled receptor signaling pathway | 0.040293704 | 6 |
| BP | GO:0014066 | regulation of phosphatidylinositol 3-kinase signaling | 0.040293978 | 10 |
| BP | GO:0048639 | positive regulation of developmental growth | 0.040393568 | 14 |
| BP | GO:0001823 | mesonephros development | 0.040498342 | 9 |
| BP | GO:0030510 | regulation of BMP signaling pathway | 0.040498342 | 9 |
| BP | GO:0010517 | regulation of phospholipase activity | 0.040768942 | 7 |
| BP | GO:0032890 | regulation of organic acid transport | 0.040768942 | 7 |
| BP | GO:0042130 | negative regulation of T cell proliferation | 0.040768942 | 7 |
| BP | GO:0042490 | mechanoreceptor differentiation | 0.040768942 | 7 |
| BP | GO:0061180 | mammary gland epithelium development | 0.040768942 | 7 |
| BP | GO:1903317 | regulation of protein maturation | 0.040768942 | 7 |
| BP | GO:0010876 | lipid localization | 0.040976664 | 30 |
| BP | GO:0006730 | one-carbon metabolic process | 0.041030946 | 5 |
| BP | GO:0014037 | Schwann cell differentiation | 0.041030946 | 5 |
| BP | GO:0030866 | cortical actin cytoskeleton organization | 0.041030946 | 5 |
| BP | GO:0046365 | monosaccharide catabolic process | 0.041030946 | 5 |
| BP | GO:0071526 | semaphorin-plexin signaling pathway | 0.041030946 | 5 |
| BP | GO:0150077 | regulation of neuroinflammatory response | 0.041030946 | 5 |
| BP | GO:0060395 | SMAD protein signal transduction | 0.042420082 | 8 |
| BP | GO:0035335 | peptidyl-tyrosine dephosphorylation | 0.042827992 | 9 |
| BP | GO:0070252 | actin-mediated cell contraction | 0.042827992 | 9 |
| BP | GO:0086011 | membrane repolarization during action potential | 0.042923926 | 4 |
| BP | GO:0090344 | negative regulation of cell aging | 0.042923926 | 4 |
| BP | GO:0032970 | regulation of actin filament-based process | 0.04311722 | 27 |
| BP | GO:0051047 | positive regulation of secretion | 0.043259873 | 22 |
| BP | GO:0001706 | endoderm formation | 0.043569706 | 6 |
| BP | GO:0070839 | metal ion export | 0.043569706 | 6 |
| BP | GO:0086002 | cardiac muscle cell action potential involved in contraction | 0.043569706 | 6 |
| BP | GO:0007548 | sex differentiation | 0.04361895 | 20 |
| BP | GO:1902305 | regulation of sodium ion transmembrane transport | 0.043649615 | 7 |
| BP | GO:0033135 | regulation of peptidyl-serine phosphorylation | 0.044034912 | 12 |
| BP | GO:0043281 | regulation of cysteine-type endopeptidase activity involved in apoptotic process | 0.044040933 | 16 |
| BP | GO:0030111 | regulation of Wnt signaling pathway | 0.044169988 | 23 |
| BP | GO:0006869 | lipid transport | 0.044240921 | 27 |
| BP | GO:0001953 | negative regulation of cell-matrix adhesion | 0.044959513 | 5 |
| BP | GO:0048634 | regulation of muscle organ development | 0.044959513 | 5 |
| BP | GO:0048678 | response to axon injury | 0.04506225 | 8 |
| BP | GO:0001710 | mesodermal cell fate commitment | 0.04511273 | 3 |
| BP | GO:0010831 | positive regulation of myotube differentiation | 0.04511273 | 3 |
| BP | GO:0038065 | collagen-activated signaling pathway | 0.04511273 | 3 |
| BP | GO:0055003 | cardiac myofibril assembly | 0.04511273 | 3 |
| BP | GO:0055119 | relaxation of cardiac muscle | 0.04511273 | 3 |
| BP | GO:0072673 | lamellipodium morphogenesis | 0.04511273 | 3 |
| BP | GO:1904355 | positive regulation of telomere capping | 0.04511273 | 3 |
| BP | GO:0071453 | cellular response to oxygen levels | 0.045535839 | 14 |
| BP | GO:0060193 | positive regulation of lipase activity | 0.04665833 | 7 |
| BP | GO:0070371 | ERK1 and ERK2 cascade | 0.046730666 | 23 |
| BP | GO:0003229 | ventricular cardiac muscle tissue development | 0.047007689 | 6 |
| BP | GO:0042220 | response to cocaine | 0.047007689 | 6 |
| BP | GO:0032535 | regulation of cellular component size | 0.047329794 | 26 |
| BP | GO:0048015 | phosphatidylinositol-mediated signaling | 0.047347468 | 14 |
| BP | GO:0010717 | regulation of epithelial to mesenchymal transition | 0.047753406 | 9 |
| BP | GO:0015837 | amine transport | 0.047753406 | 9 |
| BP | GO:0002088 | lens development in camera-type eye | 0.047810156 | 8 |
| BP | GO:0097061 | dendritic spine organization | 0.047810156 | 8 |
| BP | GO:0006814 | sodium ion transport | 0.047956985 | 18 |
| BP | GO:0006656 | phosphatidylcholine biosynthetic process | 0.047972571 | 4 |
| BP | GO:0006734 | NADH metabolic process | 0.047972571 | 4 |
| BP | GO:0022011 | myelination in peripheral nervous system | 0.047972571 | 4 |
| BP | GO:0032292 | peripheral nervous system axon ensheathment | 0.047972571 | 4 |
| BP | GO:0045737 | positive regulation of cyclin-dependent protein serine/threonine kinase activity | 0.047972571 | 4 |
| BP | GO:0051654 | establishment of mitochondrion localization | 0.047972571 | 4 |
| BP | GO:1902230 | negative regulation of intrinsic apoptotic signaling pathway in response to DNA damage | 0.047972571 | 4 |
| BP | GO:0009636 | response to toxic substance | 0.04797793 | 19 |
| BP | GO:0007043 | cell-cell junction assembly | 0.048052164 | 12 |
| BP | GO:0050673 | epithelial cell proliferation | 0.048299479 | 29 |
| BP | GO:0010939 | regulation of necrotic cell death | 0.049104935 | 5 |
| BP | GO:0045687 | positive regulation of glial cell differentiation | 0.049104935 | 5 |
| BP | GO:0071312 | cellular response to alkaloid | 0.049104935 | 5 |
| BP | GO:0006941 | striated muscle contraction | 0.049208776 | 14 |
| BP | GO:0002548 | monocyte chemotaxis | 0.04979637 | 7 |
| BP | GO:0006633 | fatty acid biosynthetic process | 0.049840723 | 13 |
| CC | GO:0000775 | chromosome, centromeric region | 3.00E-16 | 42 |
| CC | GO:0098687 | chromosomal region | 6.49E-15 | 55 |
| CC | GO:0000779 | condensed chromosome, centromeric region | 7.23E-15 | 34 |
| CC | GO:0000793 | condensed chromosome | 2.98E-14 | 44 |
| CC | GO:0000776 | kinetochore | 3.20E-14 | 32 |
| CC | GO:0009925 | basal plasma membrane | 5.41E-11 | 40 |
| CC | GO:0045178 | basal part of cell | 1.37E-10 | 41 |
| CC | GO:0005819 | spindle | 3.20E-10 | 50 |
| CC | GO:0016323 | basolateral plasma membrane | 1.80E-09 | 35 |
| CC | GO:0005874 | microtubule | 5.53E-08 | 48 |
| CC | GO:0045177 | apical part of cell | 2.16E-07 | 47 |
| CC | GO:0000940 | outer kinetochore | 3.83E-07 | 7 |
| CC | GO:0016324 | apical plasma membrane | 5.36E-07 | 41 |
| CC | GO:0000922 | spindle pole | 6.58E-07 | 25 |
| CC | GO:0005876 | spindle microtubule | 1.41E-06 | 15 |
| CC | GO:0005871 | kinesin complex | 3.32E-06 | 12 |
| CC | GO:0031261 | DNA replication preinitiation complex | 8.97E-06 | 6 |
| CC | GO:0072686 | mitotic spindle | 1.03E-05 | 22 |
| CC | GO:0062023 | collagen-containing extracellular matrix | 2.07E-05 | 41 |
| CC | GO:0005657 | replication fork | 2.45E-05 | 12 |
| CC | GO:0051233 | spindle midzone | 3.97E-05 | 9 |
| CC | GO:0071162 | CMG complex | 5.34E-05 | 5 |
| CC | GO:0005788 | endoplasmic reticulum lumen | 0.000127241 | 31 |
| CC | GO:0005604 | basement membrane | 0.00020273 | 14 |
| CC | GO:0030496 | midbody | 0.000207752 | 22 |
| CC | GO:0005902 | microvillus | 0.00041568 | 13 |
| CC | GO:0005796 | Golgi lumen | 0.000471542 | 14 |
| CC | GO:0000792 | heterochromatin | 0.000717661 | 11 |
| CC | GO:0005875 | microtubule associated complex | 0.000793607 | 18 |
| CC | GO:0005581 | collagen trimer | 0.000942011 | 12 |
| CC | GO:0000228 | nuclear chromosome | 0.001527149 | 22 |
| CC | GO:0031528 | microvillus membrane | 0.001541927 | 6 |
| CC | GO:0045120 | pronucleus | 0.002720609 | 4 |
| CC | GO:0097038 | perinuclear endoplasmic reticulum | 0.002766708 | 5 |
| CC | GO:0005911 | cell-cell junction | 0.003258367 | 38 |
| CC | GO:0031225 | anchored component of membrane | 0.003619451 | 17 |
| CC | GO:1990023 | mitotic spindle midzone | 0.003664533 | 4 |
| CC | GO:0000307 | cyclin-dependent protein kinase holoenzyme complex | 0.003674372 | 7 |
| CC | GO:0032154 | cleavage furrow | 0.003686109 | 8 |
| CC | GO:1904724 | tertiary granule lumen | 0.004653201 | 8 |
| CC | GO:0030666 | endocytic vesicle membrane | 0.005843842 | 18 |
| CC | GO:0070820 | tertiary granule | 0.00592851 | 16 |
| CC | GO:0032153 | cell division site | 0.006967202 | 9 |
| CC | GO:0031045 | dense core granule | 0.008639301 | 5 |
| CC | GO:0005881 | cytoplasmic microtubule | 0.009911922 | 9 |
| CC | GO:0045171 | intercellular bridge | 0.009911922 | 9 |
| CC | GO:0042581 | specific granule | 0.010738578 | 15 |
| CC | GO:0035579 | specific granule membrane | 0.012294063 | 10 |
| CC | GO:0000235 | astral microtubule | 0.013836283 | 3 |
| CC | GO:0005818 | aster | 0.013836283 | 3 |
| CC | GO:0000152 | nuclear ubiquitin ligase complex | 0.014804458 | 6 |
| CC | GO:0016342 | catenin complex | 0.015518221 | 5 |
| CC | GO:0030667 | secretory granule membrane | 0.016369548 | 24 |
| CC | GO:0005680 | anaphase-promoting complex | 0.016752883 | 4 |
| CC | GO:0005890 | sodium:potassium-exchanging ATPase complex | 0.017795774 | 3 |
| CC | GO:0043256 | laminin complex | 0.017795774 | 3 |
| CC | GO:0031674 | I band | 0.019049235 | 13 |
| CC | GO:0005721 | pericentric heterochromatin | 0.019711321 | 4 |
| CC | GO:0097386 | glial cell projection | 0.020032069 | 5 |
| CC | GO:1990752 | microtubule end | 0.020032069 | 5 |
| CC | GO:0001741 | XY body | 0.022318324 | 3 |
| CC | GO:0030669 | clathrin-coated endocytic vesicle membrane | 0.022417821 | 8 |
| CC | GO:0005814 | centriole | 0.023411208 | 13 |
| CC | GO:0070821 | tertiary granule membrane | 0.02415032 | 8 |
| CC | GO:0031233 | intrinsic component of external side of plasma membrane | 0.026541246 | 4 |
| CC | GO:0010369 | chromocenter | 0.027405721 | 3 |
| CC | GO:0043020 | NADPH oxidase complex | 0.027405721 | 3 |
| CC | GO:0005635 | nuclear envelope | 0.030710384 | 32 |
| CC | GO:0031091 | platelet alpha granule | 0.031480477 | 9 |
| CC | GO:0046658 | anchored component of plasma membrane | 0.03164623 | 7 |
| CC | GO:0030673 | axolemma | 0.03305513 | 3 |
| CC | GO:0031616 | spindle pole centrosome | 0.03305513 | 3 |
| CC | GO:0001917 | photoreceptor inner segment | 0.034110297 | 7 |
| CC | GO:0042383 | sarcolemma | 0.035166679 | 12 |
| CC | GO:0098858 | actin-based cell projection | 0.039783107 | 17 |
| CC | GO:0034774 | secretory granule lumen | 0.040018666 | 23 |
| CC | GO:0060205 | cytoplasmic vesicle lumen | 0.043661336 | 23 |
| CC | GO:0043596 | nuclear replication fork | 0.043982057 | 4 |
| CC | GO:0031983 | vesicle lumen | 0.046220922 | 23 |
| CC | GO:0031253 | cell projection membrane | 0.048450723 | 24 |
| CC | GO:0005901 | caveola | 0.04967178 | 8 |
| MF | GO:0008017 | microtubule binding | 1.06E-07 | 36 |
| MF | GO:0015631 | tubulin binding | 1.10E-06 | 42 |
| MF | GO:0003688 | DNA replication origin binding | 6.20E-06 | 7 |
| MF | GO:0003777 | microtubule motor activity | 7.18E-06 | 14 |
| MF | GO:0000217 | DNA secondary structure binding | 7.74E-06 | 10 |
| MF | GO:0005201 | extracellular matrix structural constituent | 5.55E-05 | 22 |
| MF | GO:0017116 | single-stranded DNA helicase activity | 9.74E-05 | 7 |
| MF | GO:0030546 | signaling receptor activator activity | 9.86E-05 | 45 |
| MF | GO:0048018 | receptor ligand activity | 0.000135047 | 44 |
| MF | GO:0008094 | ATP-dependent activity, acting on DNA | 0.000138339 | 16 |
| MF | GO:0043178 | alcohol binding | 0.000138864 | 14 |
| MF | GO:0003697 | single-stranded DNA binding | 0.000146681 | 17 |
| MF | GO:0008514 | organic anion transmembrane transporter activity | 0.00016448 | 22 |
| MF | GO:0005342 | organic acid transmembrane transporter activity | 0.000228727 | 20 |
| MF | GO:0003678 | DNA helicase activity | 0.000235304 | 12 |
| MF | GO:0005125 | cytokine activity | 0.00035419 | 25 |
| MF | GO:0048306 | calcium-dependent protein binding | 0.000447958 | 13 |
| MF | GO:0015175 | neutral amino acid transmembrane transporter activity | 0.000512824 | 8 |
| MF | GO:0046943 | carboxylic acid transmembrane transporter activity | 0.000574712 | 19 |
| MF | GO:0003774 | cytoskeletal motor activity | 0.000602211 | 15 |
| MF | GO:0015485 | cholesterol binding | 0.000784685 | 9 |
| MF | GO:0008401 | retinoic acid 4-hydroxylase activity | 0.001055327 | 4 |
| MF | GO:0050786 | RAGE receptor binding | 0.001055327 | 4 |
| MF | GO:0008574 | plus-end-directed microtubule motor activity | 0.00119916 | 5 |
| MF | GO:0005496 | steroid binding | 0.001523096 | 13 |
| MF | GO:0045236 | CXCR chemokine receptor binding | 0.00159203 | 5 |
| MF | GO:0022889 | serine transmembrane transporter activity | 0.001592456 | 4 |
| MF | GO:0005518 | collagen binding | 0.002299717 | 10 |
| MF | GO:0008509 | anion transmembrane transporter activity | 0.002549487 | 28 |
| MF | GO:0008395 | steroid hydroxylase activity | 0.002590868 | 7 |
| MF | GO:0008009 | chemokine activity | 0.00289993 | 8 |
| MF | GO:0032934 | sterol binding | 0.002955217 | 9 |
| MF | GO:0016538 | cyclin-dependent protein serine/threonine kinase regulator activity | 0.003302834 | 8 |
| MF | GO:0004222 | metalloendopeptidase activity | 0.003876859 | 13 |
| MF | GO:0005540 | hyaluronic acid binding | 0.004138313 | 5 |
| MF | GO:0008146 | sulfotransferase activity | 0.00423781 | 8 |
| MF | GO:0015291 | secondary active transmembrane transporter activity | 0.005064392 | 22 |
| MF | GO:0005212 | structural constituent of eye lens | 0.005071686 | 5 |
| MF | GO:0043138 | 3'-5' DNA helicase activity | 0.005604779 | 4 |
| MF | GO:0140097 | catalytic activity, acting on DNA | 0.006053052 | 21 |
| MF | GO:0050840 | extracellular matrix binding | 0.006705662 | 8 |
| MF | GO:0016782 | transferase activity, transferring sulphur-containing groups | 0.00689337 | 9 |
| MF | GO:0019887 | protein kinase regulator activity | 0.007455496 | 19 |
| MF | GO:0001664 | G protein-coupled receptor binding | 0.007943292 | 25 |
| MF | GO:0008028 | monocarboxylic acid transmembrane transporter activity | 0.008286431 | 8 |
| MF | GO:0015248 | sterol transporter activity | 0.008598463 | 6 |
| MF | GO:0031420 | alkali metal ion binding | 0.009018627 | 4 |
| MF | GO:0048029 | monosaccharide binding | 0.009116771 | 9 |
| MF | GO:0015171 | amino acid transmembrane transporter activity | 0.00950672 | 10 |
| MF | GO:0016209 | antioxidant activity | 0.011147757 | 10 |
| MF | GO:0015179 | L-amino acid transmembrane transporter activity | 0.01115978 | 8 |
| MF | GO:0016175 | superoxide-generating NAD(P)H oxidase activity | 0.011738553 | 3 |
| MF | GO:0051880 | G-quadruplex DNA binding | 0.011738553 | 3 |
| MF | GO:0015101 | organic cation transmembrane transporter activity | 0.012034149 | 5 |
| MF | GO:0061134 | peptidase regulator activity | 0.012653346 | 20 |
| MF | GO:0005504 | fatty acid binding | 0.012679282 | 6 |
| MF | GO:0004725 | protein tyrosine phosphatase activity | 0.013699296 | 11 |
| MF | GO:0005178 | integrin binding | 0.014066004 | 14 |
| MF | GO:0016712 | oxidoreductase activity, acting on paired donors, with incorporation or reduction of molecular oxygen, reduced flavin or flavoprotein as one donor, and incorporation of one atom of oxygen | 0.014297858 | 6 |
| MF | GO:0019207 | kinase regulator activity | 0.014415128 | 20 |
| MF | GO:0019864 | IgG binding | 0.015543339 | 3 |
| MF | GO:0017147 | Wnt-protein binding | 0.016065093 | 5 |
| MF | GO:0030506 | ankyrin binding | 0.016286297 | 4 |
| MF | GO:1901618 | organic hydroxy compound transmembrane transporter activity | 0.01650561 | 7 |
| MF | GO:0008083 | growth factor activity | 0.017037894 | 15 |
| MF | GO:0016835 | carbon-oxygen lyase activity | 0.017679727 | 9 |
| MF | GO:0042910 | xenobiotic transmembrane transporter activity | 0.018380763 | 5 |
| MF | GO:0046906 | tetrapyrrole binding | 0.018460946 | 14 |
| MF | GO:0061135 | endopeptidase regulator activity | 0.0190677 | 17 |
| MF | GO:0019200 | carbohydrate kinase activity | 0.019333831 | 4 |
| MF | GO:0016887 | ATP hydrolysis activity | 0.019940327 | 22 |
| MF | GO:0004866 | endopeptidase inhibitor activity | 0.019950438 | 16 |
| MF | GO:0004032 | alditol:NADP+ 1-oxidoreductase activity | 0.019959814 | 3 |
| MF | GO:0022858 | alanine transmembrane transporter activity | 0.019959814 | 3 |
| MF | GO:0030955 | potassium ion binding | 0.019959814 | 3 |
| MF | GO:0035325 | Toll-like receptor binding | 0.019959814 | 3 |
| MF | GO:0004714 | transmembrane receptor protein tyrosine kinase activity | 0.022677431 | 12 |
| MF | GO:0070182 | DNA polymerase binding | 0.022710612 | 4 |
| MF | GO:0020037 | heme binding | 0.023410065 | 13 |
| MF | GO:0005319 | lipid transporter activity | 0.023826364 | 14 |
| MF | GO:0032036 | myosin heavy chain binding | 0.024993094 | 3 |
| MF | GO:0140103 | catalytic activity, acting on a glycoprotein | 0.026424071 | 4 |
| MF | GO:0030291 | protein serine/threonine kinase inhibitor activity | 0.026609307 | 5 |
| MF | GO:0030414 | peptidase inhibitor activity | 0.027344161 | 16 |
| MF | GO:0042379 | chemokine receptor binding | 0.02806934 | 8 |
| MF | GO:0042562 | hormone binding | 0.029027833 | 9 |
| MF | GO:0045296 | cadherin binding | 0.030052012 | 25 |
| MF | GO:0015293 | symporter activity | 0.03017235 | 13 |
| MF | GO:0033293 | monocarboxylic acid binding | 0.030185562 | 8 |
| MF | GO:0004065 | arylsulfatase activity | 0.030642394 | 3 |
| MF | GO:0008499 | UDP-galactose:beta-N-acetylglucosamine beta-1,3-galactosyltransferase activity | 0.030642394 | 3 |
| MF | GO:0036041 | long-chain fatty acid binding | 0.030642394 | 3 |
| MF | GO:0044548 | S100 protein binding | 0.030642394 | 3 |
| MF | GO:0140666 | annealing activity | 0.030642394 | 3 |
| MF | GO:0030246 | carbohydrate binding | 0.033899587 | 21 |
| MF | GO:0070330 | aromatase activity | 0.034882197 | 4 |
| MF | GO:0019212 | phosphatase inhibitor activity | 0.035658675 | 6 |
| MF | GO:0005109 | frizzled binding | 0.036870718 | 5 |
| MF | GO:0042813 | Wnt-activated receptor activity | 0.036901798 | 3 |
| MF | GO:0045125 | bioactive lipid receptor activity | 0.036901798 | 3 |
| MF | GO:0050664 | oxidoreductase activity, acting on NAD(P)H, oxygen as acceptor | 0.036901798 | 3 |
| MF | GO:0017171 | serine hydrolase activity | 0.038098304 | 16 |
| MF | GO:0016791 | phosphatase activity | 0.038750658 | 21 |
| MF | GO:0005501 | retinoid binding | 0.040761184 | 5 |
| MF | GO:0019840 | isoprenoid binding | 0.040761184 | 5 |
| MF | GO:0016836 | hydro-lyase activity | 0.041538408 | 7 |
| MF | GO:0005506 | iron ion binding | 0.041877201 | 13 |
| MF | GO:0004713 | protein tyrosine kinase activity | 0.042126153 | 12 |
| MF | GO:0005372 | water transmembrane transporter activity | 0.043760962 | 3 |
| MF | GO:0005523 | tropomyosin binding | 0.043760962 | 3 |
| MF | GO:0019841 | retinol binding | 0.043760962 | 3 |
| MF | GO:0035173 | histone kinase activity | 0.043760962 | 3 |
| MF | GO:0072341 | modified amino acid binding | 0.044694978 | 9 |
| MF | GO:0048027 | mRNA 5'-UTR binding | 0.044734207 | 4 |
| MF | GO:0017046 | peptide hormone binding | 0.045799816 | 6 |

Tab S5. The details of KEGG enrichment analysis.

| ID | Description | pvalue | Count |
| --- | --- | --- | --- |
| hsa04110 | Cell cycle | 8.92E-16 | 34 |
| hsa04512 | ECM-receptor interaction | 2.55E-06 | 17 |
| hsa04114 | Oocyte meiosis | 0.000160547 | 18 |
| hsa04974 | Protein digestion and absorption | 0.000294207 | 15 |
| hsa04976 | Bile secretion | 0.000714863 | 13 |
| hsa05323 | Rheumatoid arthritis | 0.00109009 | 13 |
| hsa04115 | p53 signaling pathway | 0.001394464 | 11 |
| hsa04914 | Progesterone-mediated oocyte maturation | 0.002557419 | 13 |
| hsa00533 | Glycosaminoglycan biosynthesis - keratan sulfate | 0.00496195 | 4 |
| hsa04151 | PI3K-Akt signaling pathway | 0.006419049 | 30 |
| hsa00051 | Fructose and mannose metabolism | 0.006755564 | 6 |
| hsa03460 | Fanconi anemia pathway | 0.006794136 | 8 |
| hsa04218 | Cellular senescence | 0.007830846 | 16 |
| hsa04657 | IL-17 signaling pathway | 0.010081976 | 11 |
| hsa04911 | Insulin secretion | 0.014426346 | 10 |
| hsa04640 | Hematopoietic cell lineage | 0.014574081 | 11 |
| hsa04061 | Viral protein interaction with cytokine and cytokine receptor | 0.015629549 | 11 |
| hsa01230 | Biosynthesis of amino acids | 0.016358648 | 9 |
| hsa04972 | Pancreatic secretion | 0.017911395 | 11 |
| hsa04390 | Hippo signaling pathway | 0.018051419 | 15 |
| hsa05140 | Leishmaniasis | 0.019172483 | 9 |
| hsa03440 | Homologous recombination | 0.0192726 | 6 |
| hsa05219 | Bladder cancer | 0.0192726 | 6 |
| hsa04060 | Cytokine-cytokine receptor interaction | 0.021902616 | 24 |
| hsa05205 | Proteoglycans in cancer | 0.022486549 | 18 |
| hsa00010 | Glycolysis / Gluconeogenesis | 0.023592628 | 8 |
| hsa05165 | Human papillomavirus infection | 0.025966693 | 26 |
| hsa04066 | HIF-1 signaling pathway | 0.027869112 | 11 |
| hsa05230 | Central carbon metabolism in cancer | 0.029824754 | 8 |
| hsa05215 | Prostate cancer | 0.030848795 | 10 |
| hsa04977 | Vitamin digestion and absorption | 0.034796374 | 4 |
| hsa04973 | Carbohydrate digestion and absorption | 0.035407046 | 6 |
| hsa00512 | Mucin type O-glycan biosynthesis | 0.038690876 | 5 |
| hsa03030 | DNA replication | 0.038690876 | 5 |
| hsa05146 | Amoebiasis | 0.041540533 | 10 |
| hsa04918 | Thyroid hormone synthesis | 0.04257263 | 8 |
| hsa05166 | Human T-cell leukemia virus 1 infection | 0.044519793 | 18 |
| hsa00565 | Ether lipid metabolism | 0.04599652 | 6 |

Tab S6. The details of immune cell landscape.

| immune | cor | pvalue |
| --- | --- | --- |
| B cell_TIMER | 0.194640981 | 0.000319905 |
| T cell CD4+_TIMER | 0.142253942 | 0.008766608 |
| Neutrophil_TIMER | 0.310864349 | 6.01E-09 |
| Macrophage_TIMER | 0.278477566 | 1.87E-07 |
| Myeloid dendritic cell_TIMER | 0.273789539 | 3.40E-07 |
| B cell naive_CIBERSORT | -0.109945424 | 0.043076281 |
| T cell CD8+_CIBERSORT | -0.116485184 | 0.032024715 |
| T cell CD4+ memory resting_CIBERSORT | -0.162952327 | 0.002618265 |
| T cell CD4+ memory activated_CIBERSORT | 0.140815748 | 0.00942984 |
| T cell follicular helper_CIBERSORT | 0.118601816 | 0.029011006 |
| T cell regulatory (Tregs)_CIBERSORT | 0.121588088 | 0.025174302 |
| Macrophage M0_CIBERSORT | 0.270825903 | 4.13E-07 |
| Mast cell activated_CIBERSORT | -0.120386631 | 0.026661878 |
| Neutrophil_CIBERSORT | 0.174253554 | 0.001276656 |
| T cell CD4+ memory activated_CIBERSORT-ABS | 0.142278361 | 0.008708123 |
| T cell follicular helper_CIBERSORT-ABS | 0.209967425 | 9.81E-05 |
| T cell regulatory (Tregs)_CIBERSORT-ABS | 0.206320756 | 0.000130252 |
| NK cell activated_CIBERSORT-ABS | 0.114301054 | 0.035410619 |
| Macrophage M0_CIBERSORT-ABS | 0.317174184 | 2.33E-09 |
| Macrophage M2_CIBERSORT-ABS | 0.183796038 | 0.000685338 |
| Mast cell resting_CIBERSORT-ABS | 0.113468922 | 0.036778313 |
| Neutrophil_CIBERSORT-ABS | 0.197325331 | 0.000256448 |
| B cell_QUANTISEQ | 0.171129733 | 0.001563909 |
| Macrophage M1_QUANTISEQ | 0.277943649 | 1.98E-07 |
| Macrophage M2_QUANTISEQ | 0.10942002 | 0.044129957 |
| Monocyte_QUANTISEQ | 0.277314187 | 2.11E-07 |
| T cell CD4+ (non-regulatory)_QUANTISEQ | 0.170871677 | 0.001590109 |
| T cell CD8+_QUANTISEQ | 0.11639867 | 0.032153377 |
| T cell regulatory (Tregs)_QUANTISEQ | 0.1773647 | 0.001039513 |
| uncharacterized cell_QUANTISEQ | -0.21676888 | 5.95E-05 |
| T cell_MCPCOUNTER | 0.169962329 | 0.001707932 |
| B cell_MCPCOUNTER | 0.156802578 | 0.003833842 |
| Monocyte_MCPCOUNTER | 0.338693173 | 1.98E-10 |
| Macrophage/Monocyte_MCPCOUNTER | 0.338693173 | 1.98E-10 |
| Myeloid dendritic cell_MCPCOUNTER | 0.150241954 | 0.00561501 |
| B cell_XCELL | 0.164545392 | 0.002372441 |
| T cell CD4+ memory_XCELL | 0.228123933 | 2.23E-05 |
| T cell CD4+ effector memory_XCELL | -0.140995248 | 0.009338497 |
| T cell CD8+ naive_XCELL | -0.218436834 | 4.99E-05 |
| Class-switched memory B cell_XCELL | 0.159970906 | 0.003141506 |
| Common lymphoid progenitor_XCELL | 0.387742583 | 1.32E-13 |
| Endothelial cell_XCELL | -0.435804957 | 3.80E-17 |
| Eosinophil_XCELL | 0.11632309 | 0.03226614 |
| Granulocyte-monocyte progenitor_XCELL | -0.174221422 | 0.001279346 |
| Hematopoietic stem cell_XCELL | -0.350154583 | 3.26E-11 |
| Macrophage M2_XCELL | -0.256990869 | 1.63E-06 |
| Mast cell_XCELL | 0.150429354 | 0.005515467 |
| T cell CD4+ Th2_XCELL | 0.436842485 | 3.14E-17 |
| stroma score_XCELL | -0.412554174 | 0 |
| microenvironment score_XCELL | -0.219865455 | 4.63E-05 |
| Cancer associated fibroblast_EPIC | 0.156401837 | 0.003925851 |
| Macrophage_EPIC | -0.43813842 | 0 |
| uncharacterized cell_EPIC | 0.450627601 | 0 |
